# Microenvironment Modulates Tumorigenicity of Breast Cancer Cells Depending on Hormone Receptor Status

**DOI:** 10.3390/ijms27021129

**Published:** 2026-01-22

**Authors:** Priscila Pagnotta, Tomás González-Garello, María Luján Crosbie, Natalia Santiso, Anabela Ursino, Celeste Frascarolli, Alicia Amato, Rubén Dreszman, Juan Carlos Calvo, Judith Toneatto

**Affiliations:** 1 Instituto de Biología y Medicina Experimental (IBYME), Consejo Nacional de Investigaciones Científicas y Técnicas (CONICET), Vuelta de Obligado 2490, Buenos Aires 1428, Argentina; priscila.pagnotta@gmail.com (P.P.); juancalvo@ibyme.conicet.gov.ar (J.C.C.); 2 Departamento de Química Biológica, Facultad de Ciencias Exactas y Naturales, Universidad de Buenos Aires, Av. Int. Cantilo, Buenos Aires 1428, Argentina; 3 Instituto de Cálculo (IC), Facultad de Ciencias Exactas y Naturales, Universidad de Buenos Aires, Av. Int. Cantilo, Buenos Aires 1428, Argentina; tomas21.gg@gmail.com; 4 Complejo Médico Policial Churruca-Visca, Uspallata 3400, Buenos Aires 1437, Argentina; crosbieml@hotmail.com (M.L.C.); natalia_santiso@hotmail.com (N.S.); anabelaursino@yahoo.com.ar (A.U.); frascarolliceleste@gmail.com (C.F.); aliciaamato35@gmail.com (A.A.); 5 Clínica de Microcirugía, Bulnes 1813, Buenos Aires 1425, Argentina; rdreszman@fibertel.com

**Keywords:** human breast adipose tissue, tumorigenesis, hormone receptors, tumor microenvironment

## Abstract

Adipose tissue plays a crucial role in breast cancer (BC) progression by actively modulating the tumor microenvironment. We investigated how tumor proximity modifies adipose tissue by analyzing selected adipose-related and prognosis-associated markers in explants from BC patients and healthy donors. Explants were categorized by proximity to the tumor as *adjacent* (less than 2 cm), *distant* (over 2 cm), alongside *normal* explants (controls). FABP4 and vimentin expression was increased in proximity to the tumor, while caveolin-1, CD44, MMP9, and adiponectin showed minimal or no changes. Conditioned media (CM) from *adjacent* and *normal* explants were then assessed for their effects on tumorigenic traits in hormone-receptor-positive breast cancer (HR+ BC) and triple-negative breast cancer (TNBC) cell lines. *Adjacent*-CM enhanced migration, induced cytoskeletal remodeling, reduced adhesion, and promoted an elongated, motile phenotype in T47D cells. Poor-prognosis markers (caveolin-1, vimentin, CD44) were upregulated in at least one HR+ BC model, whereas Nanog and KLF4 showed modest variation. In TNBC cells, both *normal*- and *adjacent*-CM partially shifted MDA-MB-231 morphology toward a more epithelial-like state, decreasing caveolin-1 levels, while *adjacent*-CM increased MMP9 expression. Overall, these results reveal that adipose tissue-derived soluble factors exert significant and subtype-dependent effects on BC tumorigenicity.

## 1. Introduction

Breast cancer (BC)—the second most common type of cancer overall—is the most prevalent and lethal female malignancy, representing 36% of all cancer cases in women worldwide. Around 2.3 million new cases of BC and 670,000 BC-related deaths were reported globally in 2022 [1]. Estimates show that one in twenty women will be diagnosed with BC in their lifetime, and that one in seventy will die from the disease. However, incidence and mortality display considerable geographic disparities. Although incidence continues rising internationally, it is highest in industrialized countries [2]. These factors stress the critical need for further research into the biological mechanisms involved in BC and for development of improved diagnostic, therapeutic, and prognostic tools.

Several hallmarks have been proposed to describe the functional and morphological traits acquired by neoplastic cells during cancer development and progression [3,4], including key processes such as increased cellular proliferation, enhanced migratory capacity, and altered cell–cell or cell–matrix adhesion—all fundamental prerequisites for invasion and metastasis. Cells undergoing these changes often exhibit features consistent with epithelial–mesenchymal transition (EMT), including reorganization of the cytoskeleton and upregulated expression of vimentin, a mesenchymal marker found in diverse carcinomas [5]. Concurrent with EMT, remodeling of the extracellular matrix (ECM) occurs—frequently mediated by matrix metalloproteinases such as MMP9—a process that correlates with adverse prognosis and more invasive tumor phenotypes. [6,7]. Tumor cells that undergo EMT may colonize distant anatomical sites, necessitating a partial mesenchymal-epithelial transition to allow adhesion within secondary microenvironments [8].

Intratumoral genetic and phenotypic heterogeneity further complicates efficacious therapeutic interventions [9,10]. Accordingly, there is growing emphasis on cancer stem cells and the expression of pluripotency-associated transcription factors, given their putative roles in tumor aggressiveness and resistance to therapy [11]. The transcription factors SOX2, OCT4, KLF4, and c-Myc, known collectively as Yamanaka factors, can reprogram somatic cells into pluripotent stem cells, thereby inducing and maintaining this state [12]. These transcription factors are closely associated with Nanog, whose elevated expression in BC is associated with poor prognosis, whereas KLF4 expression is generally considered favorable [13,14]. Moreover, CD44—widely used as a stem cell marker in BC—functions as a co-receptor for numerous ECM ligands (e.g., hyaluronic acid), orchestrating critical oncogenic processes such as adhesion, migration, invasion, and EMT, autonomously and via cooperation with other cell-surface receptors [15].

The tumor microenvironment has emerged recently as a critical regulator of cancer hallmarks [16]. Adipose tissue (AT), the primary stromal component of the breast, plays an active role in cancer progression [17,18,19,20]. Several studies, including our own, suggest that adipocytes in the breast tumor microenvironment undergo a process of dedifferentiation that enables them to acquire tumor-promoting functions [21,22,23]. The phenotypic and functional alterations experienced by AT in the tumor microenvironment likely affect both adipocyte morphology and the repertoire of soluble mediators secreted into the peritumoral microenvironment.

This molecular and functional reprogramming of adipocytes is often accompanied by dysregulated expression of canonical adipocyte markers. Proteins such as fatty acid–binding protein 4 (FABP4) and caveolin-1—which are indicative of adipocyte maturation and function and possess prognostic relevance in various malignancies [24,25]—may also be modulated in peritumoral AT, thus contributing to disease progression.

Breast cancer comprises heterogeneous molecular subtypes that differ in hormone receptor expression, aggressiveness, and therapeutic response. For instance, triple-negative breast cancer (TNBC) exhibits greater aggressiveness and requires more complex treatments compared to hormone-receptor-positive breast cancer (HR+ BC) [26,27]. The mechanism by which soluble factors derived from tumor-associated AT modulate cancer cell behavior in relation to hormone receptor status is poorly understood. From a clinical standpoint, it is therefore pertinent to investigate the involvement of AT in BC pathobiology—explicitly, to analyze the soluble mediators released by adipose cells that may influence diverse aspects of tumor cell behavior.

Our previous research with the MCF7 cell line, an HR+ BC model, demonstrated that soluble factors secreted by the tumor-associated adipose microenvironment can modulate oncogenic traits. Conditioned media (CM) derived from tumoral AT significantly increased proliferation and migration, while decreasing cell adhesion in MCF7 cells, compared to CM from normal breast AT [28,29]. To complement these findings, we performed a proteomic characterization of CM obtained from healthy and tumoral (HR+ BC) breast AT [30]. This analysis revealed that tumor-associated CM display a markedly enriched and diversified secretome: proteins linked to immune regulation, lipid metabolism, complement system activity, and signaling pathways, as well as factors associated with EMT—all processes known to support invasion and tumor progression. Moreover, multivariate analysis identified IL-6, GRO, and MCP-2 as key cytokines that were sufficient to discriminate tumor-associated CM from CM from normal AT, highlighting the emergence of a distinct inflammatory and pro-tumorigenic profile in the adipose-derived secretome [30]. These observations raise the question of whether adipose-derived soluble mediators exert comparable effects across different HR+ BC models and whether similar influences are observed in TNBC.

We postulate that breast AT undergoes tumor-induced phenotypic alterations and secretes soluble factors that differentially regulate tumorigenic processes in a manner dependent on molecular subtype. In this study, we aimed to assess how AT adjacent to neoplastic lesions influences BC cell behavior according to hormone receptor status. We used CM obtained from AT explants from patients with HR+ BC and TNBC, and from healthy donors, and evaluated their effects on key tumor-associated features—including proliferation, migration, adhesion, morphological changes, and the expression of markers linked to pluripotency and poor prognosis—in cell line models representative of HR+ BC (MCF7 and T47D) and TNBC (MDA-MB-231).

To complement these functional studies and better define how the adipose microenvironment adapts in proximity to BC, we also quantified the expression of selected markers in AT explants. These included canonical adipocyte markers, structural or cytoskeletal components, and factors linked to ECM interaction or remodeling. Integrating these datasets allowed us to contextualize the influence of adipose-derived soluble mediators and to characterize tumor-proximity-associated features within the surrounding AT.

## 2. Results

### 2.1. Expression of Cancer Prognostic Markers in the Breast Adipose Microenvironment

**Figure 1:**
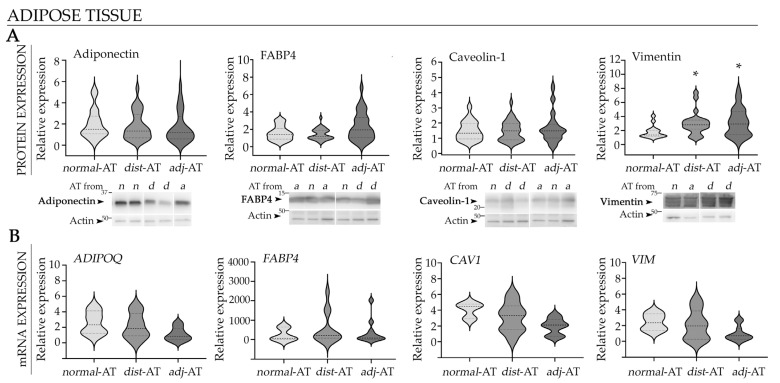
Expression levels of cancer prognostic markers in the breast adipose microenvironment. (**A**) Representative WB images and quantification of adiponectin, caveolin-1, FABP4, and vimentin in *normal*, *adjacent* and *distant* breast AT explants. Images were analyzed by densitometry. Arrows indicate the specific protein bands quantified: adiponectin band (~30 kDa), caveolin-1 doublet (~21–24 kDa), FABP4 (~16 kDa), vimentin doublet (~58 kDa), and actin (~43 kDa). Quantification values were normalized to the corresponding loading control and represented as violin plots, where the dashed (bold) line indicates the median and dotted lines represent the first and the third quartiles (*N* = 2). (**B**) The mRNA levels of *ADIPOQ*, *FABP4*, *CAV1*, and *VIM* from different AT explants were analyzed by RT-qPCR. Values were normalized to reference genes and represented as violin plots (*N* = 2). Adipose explants used in WB assay: n_*normal*-__AT_ = 9, n*_adacentj_*_-AT_ = 20 and n*_distant_*_-AT_ = 17; Adipose explants used in qPCR assay: n_*normal*-__AT_ = 4, n*_adacentj_*_-AT_ = 13 and n*_distant_*_-AT_ = 15. Statistical analysis was performed by one-way ANOVA followed by Tukey’s post hoc test; * *p* < 0.05 compared to *normal* AT. *Adjacent* breast explants (AT < 2 cm from the tumor); *distant* breast explants (AT > 2 cm from the tumor); *n*, *a* and *d* in blots represent lanes in which protein lysates from *normal*, *adjacent* and *distant* explants were seeded, respectively.

Adipocytes in the breast tumor microenvironment may undergo dedifferentiation, acquiring functions that facilitate disease progression, such as secretion of matrix metalloproteinases that promote ECM remodeling, and adoption of a migratory phenotype that enables their localization within the neoplastic lesion [21,23,29]. To further investigate whether peritumoral AT exhibits phenotypic remodeling associated with these functions, we quantified expression levels of markers indicative of mature, functional adipocytes in AT from BC patients. Samples were collected from two regions: adjacent to the tumor (≤2 cm, “*adjacent*”) and distant from the tumor (>2 cm, “*distant*”); breast AT from healthy patients was used as a control (“*normal*”). Expression levels of adiponectin and caveolin-1 remained unchanged across all explant categories (Figure 1A). FABP4—a marker of mature/functional adipocytes, with prognostic relevance in cancer [27]—showed a trend toward increased expression in *adjacent* explants, whereas vimentin displayed an increase in both *adjacent* and *distant* explants, compared to *normal* explants (Figure 1A).

In addition, mRNA expression levels of *ADIPOQ*, *FABP4*, *CAV1*, and *VIM* were quantified by qPCR (Figure 1B). *FABP4* exhibited considerable inter-sample variability, with a subset of *adjacent* and *distant* explants showing markedly increased transcript levels. In contrast, *ADIPOQ*, *CAV1*, and *VIM* showed only a modest decrease in *adjacent* explants, without statistically significant differences among the explant categories. The protein-level changes were not accompanied by significant differences in the corresponding transcripts, suggesting that post-transcriptional or post-translational mechanisms may contribute to the observed alterations in the peritumoral adipose compartment.

We also analyzed the expression of CD44 (a transmembrane glycoprotein involved in cell–cell interactions, broadly used as a stem cells marker across multiple cell types, including adipocytes, recognized as a prognostic predictor in cancer) and MMP9 (a matrix metalloproteinase implicated in ECM remodeling and basement membrane degradation). Both proteins showed only minimal, non-significant variation across categories (Supplementary Materials Figure S1).

### 2.2. Effect of CM from Normal and Adjacent AT Explants on Proliferation, Adhesion, Migration and Morphology, in the HR+ BC Models

We initially sought to functionally characterize the influence of soluble factors secreted by the tumor-associated adipose microenvironment on oncogenic hallmarks in HR+ BC models. Fundamental traits of tumor cells include proliferative signaling, evasion of growth suppressors, and acquisition of replicative immortality [16]. These processes frequently coincide with morphological alterations such as the adoption of a mesenchymal phenotype and the disruption of cell–cell and cell-basement membrane adhesion, which represent tumoral modifications favoring invasion and metastasis [8].

**Figure 2:**
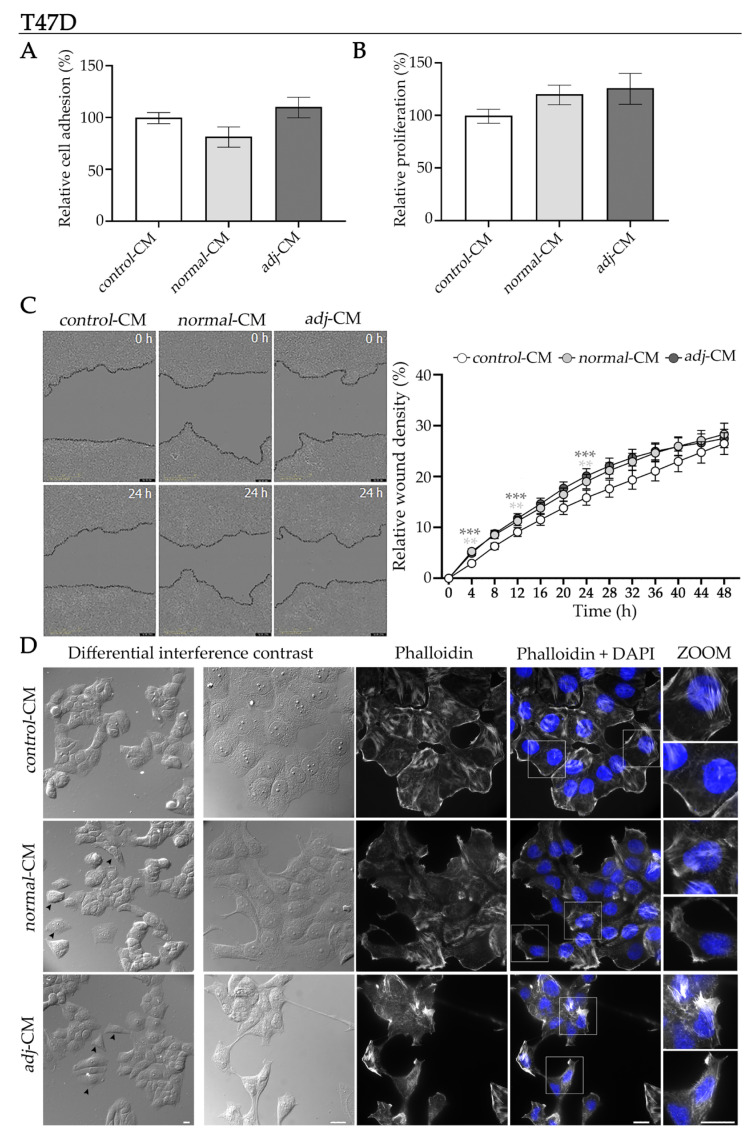
Effect of CM on proliferation, adhesion, migration, and morphology in HR+ BC molecular subtype models. (**A**) Analysis of cell adhesion, (**B**) proliferation, and (**C**) migration of the T47D cell line incubated with *control*-, *normal*-, and *adjacent*-CM. Adhesion and proliferation are expressed as percentages relative to *control*-CM. Data represent mean ± SEM (*N* = 2). CM used for adhesion assays: n*_normal_*_-CM_ = 9 and n*_adj_*_-CM_ = 10. CM used for the proliferation assay: n*_normal_*_-CM_ = 8 and n*_adj_*_-CM_ = 8. Migration is expressed as relative wound density (%)—representing the cell density within the wound area relative to the surrounding monolayer—and plotted as a function of time to assess migration kinetics. Representative images at 0 h and 24 h are shown. Data represent mean ± SEM (*N* = 2). CM used for the migration assay: n*_normal_*_-CM_ = 9 and n*_adjacent_*_-CM_ = 10. Statistical analysis was performed by one-way ANOVA followed by Tukey’s post hoc test; ** *p* < 0.01 and *** *p* < 0.001 compared to *control*-CM. (**D**) Representative differential interference contrast and confocal microscopy images of T47D cells stained with phalloidin (F-actin, white) and DAPI (nuclei, blue) after incubation with *control*-, *normal*-, or *adjacent*-CM for 24 h (*N* = 2). Boxed areas are shown as magnified images on the right (ZOOM). Scale bar = 10 μm. Arrows (➤) indicate cells exhibiting reduced cell–cell adhesion. *Adj*-CM, conditioned media from *adjacent* breast explants (AT < 2 cm from the tumor); *control*-CM, control conditioned media; *normal*-CM, conditioned media from healthy human breast adipose tissue explants.

To evaluate these phenomena, we measured the effects of CM on cell adhesion. No significant differences in adhesion were observed among *control*-, *normal*-, and *adjacent*-CM treatments (Figure 2A). We further investigated how the soluble mediators released by AT explants influenced tumor cell proliferation. We found that *adjacent*-CM showed a trend (*p* = 0.08) toward increased proliferation of T47D cells compared to *control*-CM (Figure 2B).

Migratory capacity is a distinctive characteristic of tumor cells, contributing to critical processes in tumorigenesis. To evaluate the effect of different CM on tumor cell migration, T47D cells were treated with *control*-, *normal*-, or *adjacent*-CM, and migration was monitored for 48 h using the IncuCyte^®^ system (Sartorius, Göttingen, Germany). Relative wound density was quantified over time as an indicator of wound closure (Figure 2C). T47D cells treated with *adjacent*-CM showed a significantly faster increase in relative wound density compared to *control*-CM, particularly between 4 to 24 h (Figure 2C). *Normal*-CM induced a similar, although less pronounced, increase in relative wound density. This behavior suggests that soluble factors secreted by AT—whether derived from a healthy or a tumor-associated context—can accelerate the migratory dynamics of T47D cells.

During the acquisition of an invasive phenotype and subsequent migration, malignant epithelial cells undergo a dramatic morphological shift from a static, polygonal configuration to an elongated, spindle-shaped morphology. This transformation is critically dependent on extensive actin filament reorganization. Specifically, the cortical actin associated with cell–cell adhesion is dismantled. Concurrently, F-actin stress fibers rapidly polymerize and align with the major axis of cellular elongation, driving the formation of dynamic protrusions (lamellipodia and filopodia) at the leading edge required for directional motility and tissue invasion. Simultaneously, the nucleus loses its characteristic stiffness and often acquires an elongated or irregular shape, a crucial mechanical adaptation that confers plasticity for efficient transit through the physical constraints of the dense tumor microenvironment [5].

Differential interference contrast and confocal microscopy analyses further revealed marked morphological differences induced by CM exposure. T47D cells treated with *adjacent*-CM adopted a more elongated shape and exhibited reduced cell–cell adhesion, compared to cells treated with *control*-CM (Figure 2D, differential interference contrast, bottom versus top panels), consistent with a transition toward a more motile phenotype. Concomitantly, *adjacent*-CM induced a clear reorganization of the actin cytoskeleton into stress fibers, accompanied by prominent membrane protrusions and filopodia (Figure 2D, Phalloidin + DAPI and ZOOM, bottom panels).

Reduced intercellular adhesion was also observed in T47D cells treated with *normal*-CM. However, these cells preserved polygonal morphology characteristic of epithelial cells (Figure 2D, differential interference contrast, middle). Exposure to *normal*-CM promoted reorganization of the actin cytoskeleton into stress fibers, though these fibers were less prominent than those observed in cells treated with *adjacent*-CM. (Figure 2D, Phalloidin and ZOOM, middle vs. bottom panels). In line with increased cellular plasticity and migratory capacity, nuclei of *adjacent*-CM–treated cells appeared more elongated and irregular compared to those of *control*-CM–treated cells (Figure 2D, ZOOM, bottom versus top panels). This nuclear alteration was less pronounced in *normal*-CM–treated cells (Figure 2D, ZOOM, middle panels). These observations are consistent with the functional assays described above.

### 2.3. Effect of CM from Normal and Adjacent AT Explants on Expression of Pluripotency Markers in HR+ BC Models

The expression of pluripotency markers is frequently analyzed in cancer research due to the recognized role of stem cells in tumor biology. Cancer stem cells constitute a minor tumor-initiating subpopulation that also influences processes such as disease recurrence and therapeutic resistance. We quantified protein levels of SOX2, Nanog, KLF4, and OCT4 in HR+ BC cell line models to determine whether CM modulate pluripotency marker expression in tumor cells.

**Figure 3:**
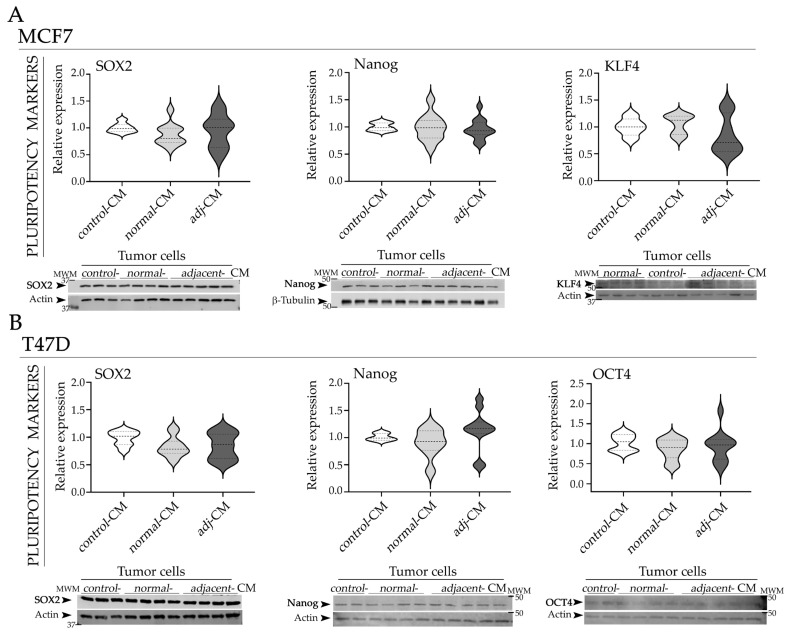
Effect of CM on the expression of pluripotency markers in HR+ BC models. (**A**) Representative WB images and quantification of SOX2, Nanog, and KLF4 or OCT4, in MCF7 and (**B**) T47D cells incubated with *control*-CM, *normal*-CM, and *adjacent*-CM. Images were analyzed by densitometry. Arrows (➤) indicate the specific protein bands quantified: SOX2 (~34 kDa), Nanog (~40 kDa), KLF4 (~53 kDa), OCT4 (~45 kDa), actin (~43 kDa), and β-Tubulin (55 kDa). Quantification values were normalized to the corresponding loading control and subsequently expressed relative to *control*-CM condition. Data are represented as violin plots, where the dashed (bold) line indicates the median and dotted lines represent the first and the third quartiles (*N* = 2–3). CM used in the assays: n_*normal*-__CM_ = 14 and n*_adacentj_*_-CM_ = 20 for MCF7 and n*_normal_*_-CM_ = 15 and n*_adjacent_*_-CM_ = 20 for T47D. *Adj*-CM, conditioned media from *adjacent* breast explants (AT < 2 cm from the tumor); *control*-CM, control conditioned media; *normal*-CM, conditioned media from human healthy breast adipose tissue explants; MWM, molecular weight markers.

In MCF7 cells, a decreasing trend in KLF4 expression was observed following treatment with *adjacent*-CM compared to *normal*-CM (Figure 3A), whereas SOX2 and Nanog levels remained unchanged with all treatments. OCT4 expression showed no significant variation (Supplementary Materials Figure S2).

In T47D cells, *adjacent*-CM induced an increasing trend in Nanog expression compared to *control*- and *normal*-CM (Figure 3B). Expression levels of SOX2 and OCT4 (Figure 3B) and KLF4 (Supplementary Materials Figure S2) remained unchanged with all treatments, showing no significant variation.

### 2.4. Effect of CM from Normal and Adjacent AT Explants on Expression of Prognostic Markers in HR+ BC Models

Given the observed morphological and functional alterations, we evaluated the expression of proteins that are implicated in these processes and are linked to cancer prognosis.

**Figure 4:**
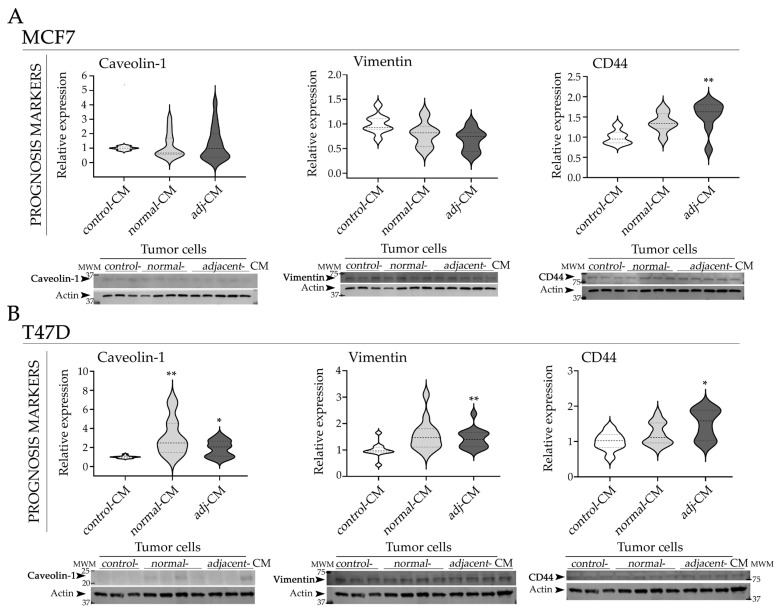
Effect of CM on the protein expression of prognostic markers in HR+ BC models. (**A**) Representative WB images and quantification of caveolin-1, vimentin, and CD44, in MCF7, and (**B**) T47D cells incubated with *control*-CM, *normal*-CM, and *adjacent*-CM. Images were analyzed by densitometry. Arrows (➤) indicate the specific protein bands quantified: caveolin-1 (~21–24 kDa), vimentin (~58 kDa), CD44 (~80 kDa), and actin (~43 kDa). Quantification values were normalized to the corresponding loading control and subsequently expressed relative to *control*-CM condition. Data are represented as violin plots, where the dashed line indicates the median and dotted lines represent the first and the third quartiles (*N* = 2–3). CM used in the assays: n_*normal*-__CM_ = 14 and n*_adacentj_*_-CM_ = 20 for MCF7 and n*_normal_*_-CM_ = 15 and n*_adjacent_*_-CM_ = 20 for T47D. Statistical analysis was performed by one-way ANOVA followed by Tukey’s post hoc test; * *p* < 0.05, and ** *p* < 0.01 compared to *control*-CM. *Adj*-CM, conditioned media from *adjacent* breast explants (AT < 2 cm from the tumor); *control*-CM, control conditioned media; *normal*-CM, conditioned media from human healthy breast adipose tissue explants; MWM, molecular weight markers.

In MCF7 cells, treatment with *adjacent-CM* induced a significant increase in CD44 compared to *control*-CM. In addition, both *normal*- and *adjacent*-CM showed a tendency toward increased caveolin-1 expression after 24 h of treatment (Figure 4A). MMP9 expression showed no significant variation among treatments (Supplementary Materials Figure S2).

In T47D cells, both *adjacent*- and *normal*-CM increased the expression of caveolin-1 compared with *control*-CM (Figure 4B). Notably, the effect of *normal*-CM on caveolin-1 expression was greater than that observed with *adjacent*-CM. The effect on CD44 expression was similar to that observed in MCF7 cells after 24 h of treatment with *adjacent*-CM. Vimentin levels were also increased in T47D cells after treatment with *adjacent*-CM, compared to *control*-CM. MMP9 expression showed no significant variation among treatments (Supplementary Materials Figure S2).

In addition, mRNA expression levels of *CAV1* and *VIM* were quantified by qPCR in both MCF7 and T47D cells. Both HR+ BC cell lines showed similar expression pattern; no significant differences were observed (Supplementary Materials Figure S2).

### 2.5. Effect of CM from Normal and Adjacent AT Explants on Proliferation, Adhesion, Migration and Morphology, in the TNBC Model

MDA-MB-231 cells displayed a markedly more aggressive phenotype compared to HR+ BC models, characterized by high basal motility, reduced cell–cell adhesion, and an elongated, spindle-like morphology consistent with a mesenchymal state. For this reason, we explored preliminarily the role on MDA-MB-231 cell behavior of soluble factors released by the adipose tumor microenvironment of patients with TNBC.

**Figure 5:**
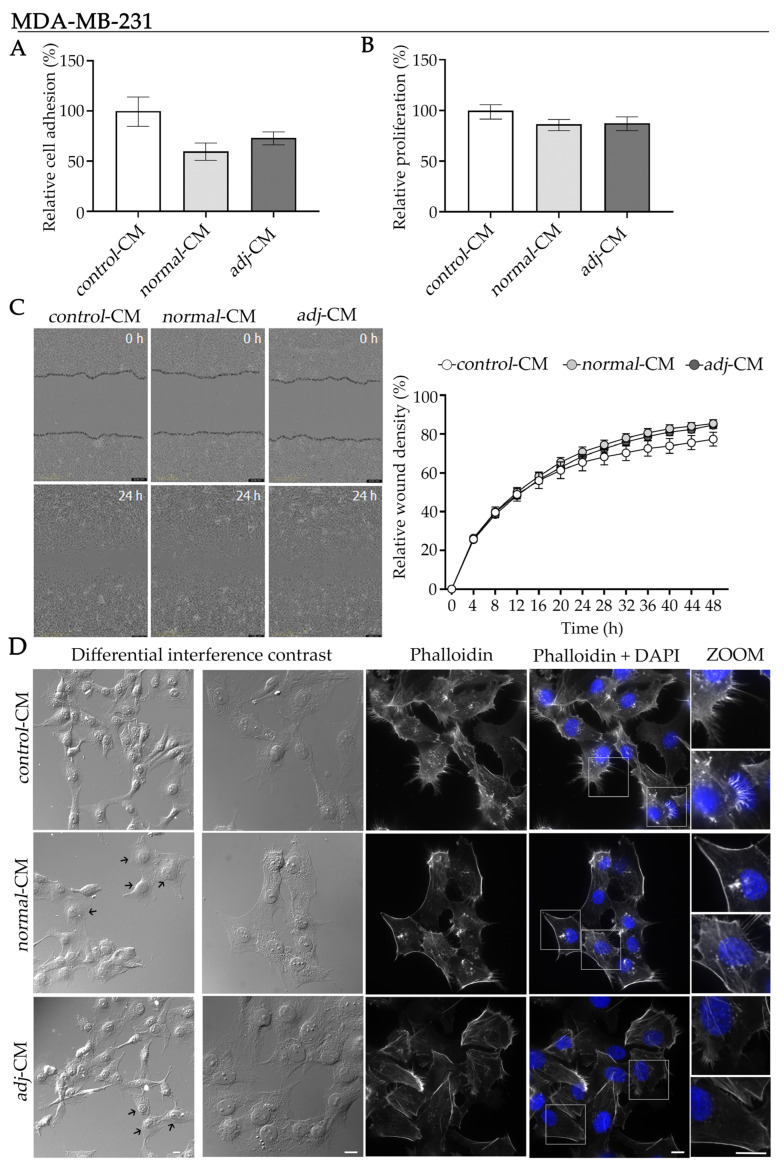
Effect of CM on proliferation, adhesion, migration, and morphology in the TNBC model. (**A**) Analysis of cell adhesion, (**B**) proliferation, and (**C**) migration of the MDA-MB-231 cell line incubated with *control*-, *normal*- and *adj*-CM. Adhesion and proliferation are expressed as percentages relative to *control*-CM. Data represent mean ± SEM (*N* = 2). CM used for adhesion assays: n*_normal_*_-CM_ = 8 and n*_adj_*_-CM_ = 4. CM used for the proliferation assay: n*_normal_*_-CM_ = 12 and n*_adj_*_-CM_ = 4. Migration is expressed as relative wound density (%)—representing the cell density within the wound area relative to the surrounding monolayer—and plotted as a function of time to assess migration kinetics. Representative images at 0 h and 24 h are shown. Data represent mean ± SEM (*N* = 2). CM used for the migration assay: n*_normal_*_-CM_ = 9 and n*_adj_*_-CM_ = 5. (**D**) Representative differential interference contrast and confocal microscopy images of MDA-MB-231 cells stained with phalloidin (F-actin, white) and DAPI (nuclei, blue) after incubation with *control*-, *normal*-, or *adjacent*-CM for 24 h (*N* = 2). Boxed areas are shown as magnified images on the right (ZOOM). Scale bar = 10 μm. Arrows (➜) indicate cells exhibiting polygonal and epithelial-like appearance. *Adj*-CM, conditioned media from *adjacent* breast explants (AT < 2 cm from the tumor); *control*-CM, control conditioned media; *normal*-CM, conditioned media from human healthy breast adipose tissue explants.

Incubation of MDA-MB-231 cells with *normal*-CM induced a tendency toward decreased cell adhesion, compared to *control*-CM (Figure 5A). In contrast, no significant differences were observed in proliferation under the tested conditions (Figure 5B). Wound-healing analysis revealed that during the initial phase (up to ∼12 h), the increase in relative wound density progressed at comparable rates across all experimental conditions (Figure 5C). However, at later time points (∼20–48 h), the relative wound density curves of all groups continued to rise but displayed a reduced slope. Under these conditions, cells treated with *normal*- and *adjacent*-CM exhibited similar kinetics, which diverged from the *control*-CM group (Figure 5C). Overall, no significant differences in migratory capacity were detected after 48 h of treatment with either *normal*- or *adjacent*-CM compared to *control*-CM.

Although functional assays did not reveal significant differences among different CM analyzed, morphological analysis can provide additional insight into early or subtle cellular responses. Therefore, we next examined whether CM exposure induced detectable structural or cytoskeletal changes. Both *normal*- and *adjacent*-CM showed a partial reversion of the mesenchymal phenotype characteristic of MDA-MB-231 cells, promoting a shift from the typical elongated, spindle-like morphology toward a more polygonal or epithelial-like appearance (Figure 5D, differential interference contrast, middle and bottom versus top panels). This phenotypic change was accompanied by the formation of cell clusters, which were not evident in the *control*-CM condition. Moreover, both *normal*- and *adjacent*-CM induced a marked reorganization of F-actin in MDA-MB-231 cells, resulting in reduced presence of filopodia- and lamellipodia-like membrane protrusions, compared to the *control*-CM group (Figure 5D, ZOOM, middle and bottom versus top panels). No nuclear alteration was observed (Figure 5D, ZOOM panels).

### 2.6. Effect of CM from Normal and Adjacent AT Explants on Expression of Pluripotency and Prognostic Markers in the TNBC Model

**Figure 6:**
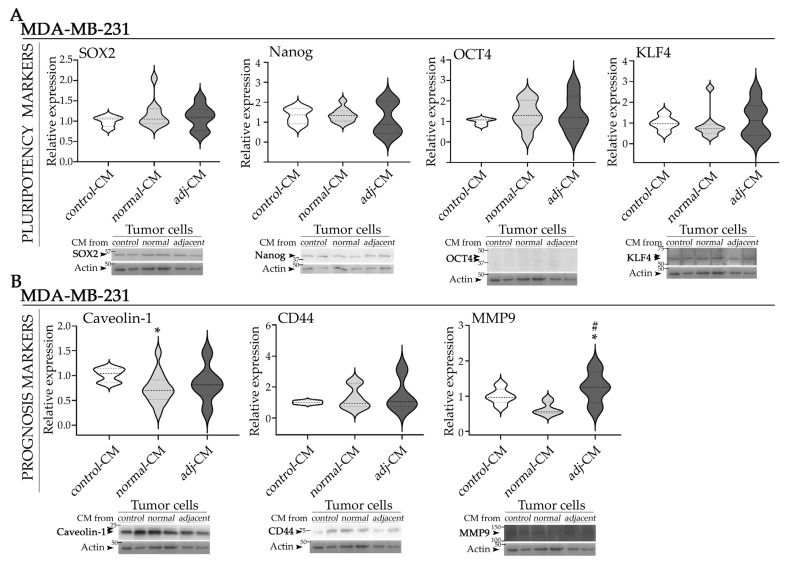
Effect of CM on the expression of pluripotency and prognostic markers in the TNBC model. (**A**) Representative WB images and quantification of SOX2, Nanog, OCT4, and KLF4 and (**B**) caveolin-1, CD44 and MMP9 in MDA-MB-231 cells incubated with *control*-CM, *normal*-CM and *adjacent*-CM. Images were analyzed by densitometry. Arrows (➤) indicate the specific protein bands quantified: SOX2 (~34 kDa), Nanog (~40 kDa), OCT4 (~45–39 kDa), KLF4 (~53 kDa), caveolin-1 (~21–24 kDa), CD44 (~80 kDa), MMP9 (~100–150 kDa) and actin (~43 kDa). Quantification values were normalized to the corresponding loading control and subsequently expressed relative to *control*-CM condition. Data are represented as violin plots, where the dashed line indicates the median and dotted lines represent the first and the third quartiles (*N* = 2). CM used in the assays: n_*normal*-__CM_ = 9 and n*_adacentj_*_-CM_ = 4 for pluripotency markers and n_*normal*-__CM_ = 9 and n*_adacentj_*_-CM_ = 3 for prognosis markers. Statistical analysis was performed by one-way ANOVA followed by Tukey’s post hoc test; * *p* < 0.05 compared to *control*-CM; # *p* < 0.05 compared to *normal*-CM. *Adj*-CM, conditioned media from *adjacent* breast explants (AT < 2 cm from the tumor); *control*-CM, control conditioned media; *normal*-CM, conditioned media from human healthy breast adipose tissue explants; MWM, molecular weight markers.

The protein expression levels of the pluripotency-associated transcription factors SOX2, Nanog, OCT4, and KLF4 remained unchanged following incubation with different CM (Figure 6A). In contrast, while *normal*-CM induced a significantly decreased expression of the prognostic markers caveolin-1, and MMP9 (trend), *adjacent*-CM induced increased MMP9 levels relative to *control*-CM (Figure 6B). CD44 expression showed no significant variation among treatments (Figure 6B). We could not evaluate the effect on vimentin expression due to the limited number of patients with TNBC.

### 2.7. Comparative Analysis of the Effect of Adipose-Derived CM Between Breast Cancer Molecular Subtype Models

We conducted a comparative analysis of the effect that CM have on different BC cell lines, assessing functional and molecular variables to investigate the tumorigenic potential of the adipose tumor microenvironment across heterogeneous models.

**Figure 7:**
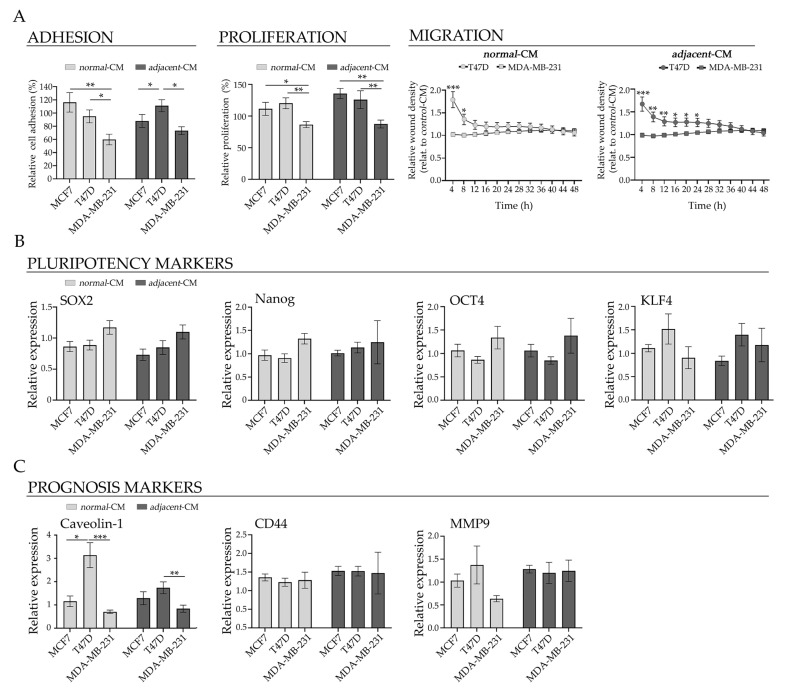
Comparative effect of CM in different breast cancer molecular subtype models. Comparative analysis of (**A**) functional assays and (**B**) pluripotency and (**C**) prognosis markers of the MCF7, T47D and MDA-MB-231 cell lines incubated with *normal*- and *adjacent*-CM. Adhesion and proliferation are expressed as a percentage relative to *control*-CM. Migration is expressed as relative wound density (%) over time at 4 h intervals relative to *control*-CM. Protein expression of SOX2, Nanog, OCT4, KLF4, caveolin-1, CD44 and MMP9 are expressed relative to *control*-CM. Data represent mean ± SEM. Statistical analysis was performed by one-way ANOVA followed by Tukey’s post hoc test; * *p* < 0.05, ** *p* < 0.01, and *** *p* < 0.001. *Adjacent*-CM, conditioned media from *adjacent* breast explants (AT < 2 cm from the tumor); *control*-CM, control conditioned media; *normal*-CM, conditioned media from human healthy breast adipose tissue explants.

Cell adhesion was decreased in the TNBC model after incubation with *normal*- and *adjacent*-CM, compared to the effect in the HR+ BC model (Figure 7A). A similar pattern was found for proliferative capacity, with significant differences between the TNBC and HR+ BC models under either *adjacent*- and *normal*-CM treatments (Figure 7A).

Furthermore, the CM generated a differential effect on migration between the T47D and MDA-MB-231 lines: both the *normal*- and *adjacent*-CM induced an increase in migration in T47D cells, compared to the MDA-MB-231 line, with incubation times of 4 to 12 h and 4 to 28 h, respectively (Figure 7A).

The expression of the pluripotency markers SOX2 and Nanog was differentially modulated across the study models. *Normal*-CM elicited a reduction in SOX2 protein levels in T47D and MCF7 cells relative to MDA-MB-231 cells, whereas *adjacent*-CM decreased SOX2 expression exclusively in MCF7 cells compared to MDA-MB-231 cells (Figure 7B). A decrease in Nanog expression was observed in both T47D and MCF7 cells incubated with *normal*-CM, compared to the MDA-MB-231 line (Figure 7B). However, no significant differences were detected between the TNBC and HR+ BC models with either *adjacent*- and *normal*-CM treatments (Figure 7B). Consistent with these observations on pluripotency markers, the CM also induced differential effects on the expression of additional prognostic markers across the studied models. *Normal*-CM increased caveolin-1 expression in T47D cells compared to MCF7 and MDA-MB-231 cells, both of which showed lower levels than T47D, consistent with the pattern observed after incubation with *adjacent*-CM in the HR+ BC model (Figure 7C). The decreased caveolin-1 expression in MDA-MB-231 cells reached statistical significance under *normal*- and *adjacent*-CM treatment when compared with T47D cells. Moreover, *normal*-CM reduced MMP9 expression in the MDA-MB-231 cells compared to the HR+ BC models, whereas no changes were observed under *adjacent*-CM (Figure 7C). We did not observe a significant differential effect on the expression of CD44 between both the TNBC and HR+ BC models (Figure 7C).

## 3. Discussion

The role of the tumor microenvironment is increasingly recognized as a major regulator of BC progression [16]. As the predominant stromal component, adipose tissue contributes to tumorigenesis by providing metabolic, inflammatory, and structural cues [21,22,28,29]. We characterized functional and prognostic markers in the adipose microenvironment and showed how its soluble factors modulate tumor-cell phenotypes. FABP4 and vimentin levels increased in *adjacent* AT, with vimentin also elevated in *distant* AT. Proteomic analysis of human AT-CM supports these findings, as both proteins were previously identified in tumor-associated secretomes [30]. Their intracellular upregulation and enhanced release are consistent with their roles in BC aggressiveness: FABP4 supports fatty acid transfer and activates PI3K-AKT and MAPK-ERK pathways [31,32], whereas soluble vimentin reflects EMT activity and poor prognosis, especially in TNBC. FABP4-driven metabolic signaling cooperates with vimentin-mediated plasticity, generating a tumor-promoting circuit [33,34,35]. Vimentin also participates in lipid-droplet dynamics and interacts with FABP4 in adipocytes [36,37]. The concurrent elevation of these proteins in *adjacent* AT suggests tumor-induced metabolic remodeling that favors fatty acid supply and tumor progression.

Previous immunohistochemical studies showed increased CD44 and caveolin-1 in *adjacent* and *distant* AT [29], whereas in our study CD44 showed only a slight increase and *CAV1* mRNA decreased. These differences likely reflect cellular resolution, supporting adipocyte heterogeneity. Similar discrepancies in lipolytic markers (manuscript under revision) further support distinct adipocyte subpopulations. Loss of stromal caveolin-1 is linked to autophagy, oxidative stress, and poor BC outcomes [24]. The presence of CD44 also suggests a less differentiated adipocyte subset [29,38].

MMP9 levels did not differ among AT groups, indicating that adipocytes may not store significant intracellular MMP9, and that regulation may occur mainly at the level of secretion, consistent with our previous findings showing increased MMP9 enzymatic activity in CM derived from tumor-associated AT explants [28]. Overall, *adjacent* AT displayed coordinated increases in FABP4 and vimentin, consistent with metabolically active, tumor-conditioned adipocytes, whereas *distant* AT showed no coherent remodeling.

We next examined how adipose-derived soluble factors influence HR+ BC and TNBC cells. Consistent with our previous work [28,29], CM modulated proliferation, adhesion, and migration in HR+ BC models. Bioinformatic analysis showed that T47D presents higher basal *CD44*, *MMP9*, and pluripotency markers than MCF7, suggesting a more plastic baseline state (Supplementary Materials Table S1). Accordingly, *adjacent*-CM more prominently affected proliferation and adhesion in MCF7, while in T47D it mainly increased migration. A small reduction in adhesion after *normal*-CM may represent a protective effect in T47D. Morphologically, T47D maintained epithelial traits after *normal*-CM.

Given the EMT-related proteins detected in tumor-associated CM, we found that soluble adipose cues altered pluripotency markers in HR+ BC cells. *Adjacent*-CM induced a modest increase in Nanog in T47D, whereas KLF4 showed a slight reduction in MCF7. These effects are consistent with the known prognostic roles of these factors [13,14] and with the proliferation trends observed in each model [39,40,41,42,43]. Higher basal *KLF4* and *SOX2* in T47D may attenuate CM-induced changes. These results support early phenotypic reprogramming of HR+ BC by adipose-derived factors.

*Adjacent*-CM also increased caveolin-1, vimentin, and CD44 in HR+ BC cells, markers associated with proliferation, invasion, and mesenchymal traits [44,45,46]. These molecular changes aligned with reduced cell–cell contacts, cytoskeletal remodeling, and increased mesenchymal morphology, consistent with enhanced proliferation and migration. Importantly, the use of two HR+ cell lines (MCF7 and T47D) provided complementary information and allowed us to partially capture the intrinsic heterogeneity of HR+ BC. This design strengthened our interpretation, showing that adipose-derived cues may differentially shape phenotypic programs even within the same molecular subtype.

TNBC displayed distinct responses. Bioinformatic analysis showed that MDA-MB-231 cells overexpress *SOX2* and *MMP9* but show lower *KLF4*, *CD44*, *VIM*, and *CAV1* relative to HR+ BC lines (Supplementary Materials Table S1). *Adjacent*-CM did not alter proliferation or migration, likely due to the intrinsically aggressive phenotype of this line. Adhesion decreased with both CM types, which may represent a protective response. Migration patterns confirmed their mesenchymal behavior. Morphologically, CM shifted MDA-MB-231 toward a more cohesive, epithelial-like appearance, contrasting with the mesenchymal shift induced in HR+ BC models. Although, *adjacent*-CM did not markedly alter pluripotency markers in TNBC, interpretation of EMT-related pathways remains partially limited by the absence of vimentin evaluation in TN samples, which would have contributed additional mechanistic insight into stromal–tumor interactions in this subtype.

*Normal*-CM reduced caveolin-1 and MMP9 in MDA-MB-231 cells, proteins linked to poor TNBC prognosis [47,48,49,50,51]. *Adjacent*-CM also increased MMP9 expression in MDA-MB-231 cells, a finding that requires verification at the level of enzymatic activity to determine its functional relevance. Overall, CM effects on TNBC were weaker than in HR+ BC, and in some cases appeared protective. Further proteomic profiling of TNBC-derived CM is needed to clarify these subtype-specific responses.

Although our TNBC sample size was limited, these observations highlight the value of exploring AT in BC pathobiology. Future studies will expand sample numbers, evaluate additional hallmarks, and investigate metabolic and angiogenic effects of AT-tumor communication.

**Figure 8:**
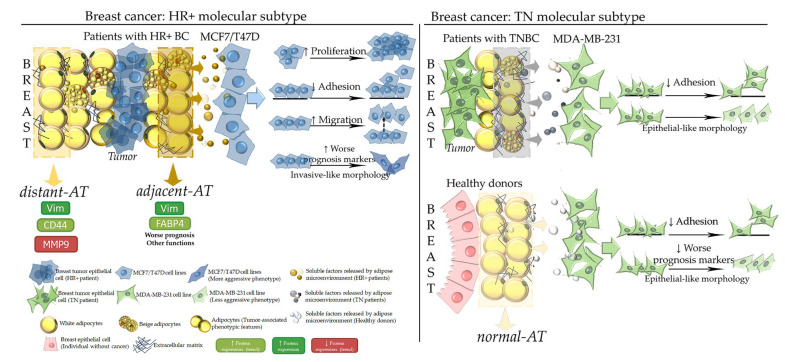
Scheme of the findings obtained in this research. Representation of the results distinguishing between the HR+ BC and TNBC models. *Adjacent* breast adipose tissue explants (AT < 2 cm from the tumor); *distant* breast adipose tissue explants (AT > 2 cm from the tumor); HR+ BC, hormone-receptor-positive breast cancer molecular subtype (estrogen- and progesterone- receptor-positive and HER2-negative); TNBC, triple-negative breast cancer molecular subtype; Vim, vimentin.

In summary, *adjacent* breast AT in HR+ BC undergoes remodeling that establishes a pro-tumorigenic niche, reflected in altered lipid-handling proteins, cytoskeletal components, and secretome composition. HR+ BC cells showed increased proliferation, migration, and mesenchymal features upon exposure to adipose-derived factors. TNBC cells, already highly aggressive, not only did not exhibit further pro-tumorigenic reinforcement: in some cases, they showed partial phenotypic reversion. A schematic summary is shown in Figure 8.

## 4. Materials and Methods

### 4.1. Reagents

General reagents were obtained from Sigma-Aldrich/Merck Chemical Co. (St. Louis, MO, USA), and molecular biology reagents were purchased from Invitrogen (Carlsbad, CA, USA). Primary and secondary antibodies were purchased from Abcam (Cambridge, MA, USA), Santa Cruz Biotechnology (Dallas, TX, USA), R&D Systems (Bio-Techne Co, Minneapolis, MN, USA), BD Biosciences (Becton, Dickinson and Company, San Jose, CA, USA), Invitrogen, and Sigma-Aldrich/Merck Chemical Co.

### 4.2. Sample Collection and Handling

This study was approved by the ethics committees of Complejo Médico Policial Churruca-Visca and Instituto de Biología y Medicina Experimental (IBYME; CE025). All individuals included in this study gave their informed consent and underwent mastectomy at Complejo Médico Policial Churruca-Visca using a standardized tissue acquisition protocol. Human AT samples were collected from patients with infiltrating ductal carcinoma who had not been previously treated with chemotherapy or radiotherapy. The study included HR+ BC (n = 51) and TNBC (n = 5) cases. Tissues were obtained from sites adjacent to (<2 cm; “*adjacent*”) and distant (>2 cm; “*distant*”) from the tumor. Control AT (*normal*) was collected from healthy women undergoing elective breast surgery for aesthetic reasons (n = 30), also with informed consent. Clinicopathological variables such as body mass index and menopausal status were not considered in this study.

Samples were processed as previously described [28]. In brief, tissues were transported in phosphate-buffered saline (PBS) containing 50 μg gentamicin/mL (Gentamicin sulfate, Richet laboratory) and processed in a laminar flow hood within 2 h of surgical excision. Each sample was divided for formalin-fixed paraffin embedding and snap-freezing. Frozen samples were stored at −80 °C until use (Section 4.6 and Section 4.8). For histological analysis, AT samples were fixed in 4% buffered formaldehyde, embedded in paraffin, sliced at 3 μm, and stained with hematoxylin and eosin (H&E). A pathologist confirmed the composition of the AT using optical microscopy.

### 4.3. Preparation of Conditioned Media

Conditioned media (CM) were prepared as previously described [28]. Briefly, breast AT collected from *adjacent* and *normal* explants was incubated with M199 medium (Invitrogen^TM^) at a ratio of 0.1 g of tissue per 1 mL of medium for 1 h at 37 °C in a 5% CO_2_ atmosphere. The medium was then replaced, and tissues were incubated for an additional 24 h. The resulting supernatants were collected and subjected to centrifugation (3 min at 400× *g*) to remove cells, filtered through 0.22 μm filters, and aliquoted (0.25–0.5 mL). The CM obtained from *adjacent* and *normal* explants were stored at −80 °C until required. Control CM (*control*-CM) was prepared by incubating serum-free M199 medium under the same conditions, in the absence of tissue. For functional assays, equal volumes of CM were used. All experiments were conducted using individual CM preparations derived from independent AT explants; pooled CM were not used in any assay.

### 4.4. Culture of Breast Tumor Epithelial Cell Lines

The immortalized human breast tumor epithelial cell lines MCF7, T47D (molecular subtype ER+/PR+/HER2−; luminal subtype, HR+ BC) and MDA-MB-231 (ER−/PR−/HER2−; claudin-low basal subtype, TNBC) were cultured in D-MEM/F-12 medium supplemented with 10% fetal bovine serum (FBS) and maintained at 37 °C in a humidified 5% CO_2_ atmosphere.

### 4.5. Treatment with Normal-CM and Adjacent-CM

To assess the effect of CM on the expression levels of pluripotency- and prognosis-related markers, MCF7, T47D and MDA-MB-231 cells were seeded into 24- or 12-well plates and cultured in complete medium until reaching 75–80% confluence. The medium was then aspired and cells were washed twice with PBS. Subsequently, cells were incubated at 37 °C for 24 h with *normal*-, *adjacent*-, or *control*-CM, each diluted 1:1 in D-MEM/F-12 containing 2% BSA (final concentration 1% BSA). After treatment, cells grown in 24- and 12-well plates were used for total RNA extraction and protein lysate preparation, respectively.

### 4.6. Total Protein Lysates

Protein lysates were prepared from frozen *normal* (n = 9), *adjacent* (n = 20), and *distant* (n = 17) AT explants. Tissues were homogenized in RIPA buffer [50 mM Tris-HCl (pH 7.5), 150 mM NaCl, 1% Igepal CA-630 (NP- 40), 0.5% sodium deoxycholate, 0.1% sodium dodecyl sulfate (SDS)] supplemented with protease and phosphatase inhibitors (1 mL per 200 mg of tissue) using an Ultra-Turrax homogenizer (IKA, Staufen, Germany) on ice. Homogenates were centrifuged at 13,400× *g* for 15 min at 4 °C; supernatants were recovered, and a second centrifugation was performed for 5 min under the same conditions. For tumor cells lines, total protein lysates were obtained after 24 h of CM treatment (*adjacent*-, *normal*- or *control*-CM) using a lysis buffer containing 60 mM Tris-HCl (pH 6.8), and 1% SDS. Protein concentrations were determined using the DC Protein Assay kit (Bio-Rad Laboratories, Inc.), and lysates were stored at −80 °C until further analysis.

### 4.7. Western Blot Assay

Proteins (15–50 μg) were separated by denaturing electrophoresis on 12% SDS-PAGE (acrylamide #15512-023; N,N′-methylenebisacrylamide #15516-024, Invitrogen, Carlsbad, CA, USA). Gels were prepared with 15 well combs, and molecular weight markers (Precision Plus Protein™ All Blue Standards #161-0373, Bio-Rad, laboratories, Hercules, CA, USA) were loaded at both ends of each gel. To minimize potential loading biases and experimental artifacts during detection, the samples were randomly assigned to lanes. Electrophoresis was conducted at a constant voltage of 100 V for approximately 2 h. Proteins were then electrotransferred onto PVDF membranes for 90 min at a constant voltage of 80 V. Membranes were blocked with 1% bovine serum albumin (Sigma-Aldrich, St. Louis, MO, USA; REF10735086001) in TBS with Tween 20 (0.05% TBS-T) for 1 h at room temperature and subsequently incubated overnight at 4 °C with specific primary antibodies. Following the washing steps, the membranes were incubated with horseradish peroxidase-conjugated secondary antibodies for 90 min at RT. Protein detection was performed using a chemiluminescence reaction with an ECL solution containing luminol (Sigma-Aldrich, St. Louis, MO, USA; 123072), p-coumaric acid (Sigma-Aldrich, St. Louis, MO, USA; C9008), and H_2_O_2_. Band density was quantified using ImageJ software (version 1.52p; NIH, Bethesda, MD, USA). Only bands fulfilling predefined quality criteria were included in the analysis. Blots or bands were excluded when exhibiting: (i) heterogeneous or noisy membrane background; (ii) nonspecific signal overlapping the band of interest; or (iii) poorly defined, distorted, or fragmented bands. Actin or β-tubulin was used as a loading control, selected according to the molecular weight of the target protein and the distribution of bands within each gel to ensure accurate normalization. Detailed information on the primary and secondary antibodies, including sources and working dilutions, is provided in Supplementary Materials Table S2. The original, unprocessed Western blot membranes corresponding to all experiments are included in Supplementary Materials Figures S3–S6.

### 4.8. Gene Expression by RT-qPCR Analysis

Total RNA was extracted using Tri Reagent (Molecular Research Center, Cincinnati, OH, USA; cat. no. TR118) according to the manufacturer’s instructions. Briefly, adipose tissue explants (0.1–0.2 g) were mixed with Tri Reagent and lysed using an Ultraturrax homogenizer. Samples were centrifuged at 12,000× *g* for 10 min at 4 °C, and the non-fatty supernatant was collected. After incubation at RT for 5 min, 0.1 mL of chloroform was added, followed by vigorous vortexing and centrifugation at 12,000× *g* for 15 min at 4 °C. The aqueous phase was transferred to a new tube, mixed with 0.25 mL of isopropanol, and incubated overnight at 4 °C. After 10 min at room temperature, samples were centrifuged again (12,000× *g*, 8 min, 4 °C). The resulting RNA pellet was washed twice with 0.5 mL of 75% ethanol by vortexing and centrifuging at 7500× *g* for 5 min at 4 °C. Ethanol was removed, and samples were air-dried and incubated at 55 °C. Finally, the pellet was resuspended in RNase-free water and incubated at 55 °C for 15 min.

For tumor cell lines, mRNA was obtained after 24 h of treatment with conditioned media (*normal*-, *adjacent*- or *control*-CM). Total RNA was extracted using Tri Reagent following the manufacturer’s instructions. RNA concentration and purity were determined spectrophotometrically using a NanoDrop™ 2000 (Thermo Fisher Scientific, Waltham, MA, USA), considering samples with A260/A280 ratios between 1.8 and 2.0 as acceptable. RNA integrity was verified by electrophoresis on 1% agarose gels. Aliquots of purified RNA were stored at −80 °C until reverse transcription.

Complementary DNA (cDNA) was synthesized from 800 ng of total RNA using the iScript™ cDNA Synthesis Kit (Bio-Rad, Hercules, CA, USA; cat. no. 1708891), according to the manufacturer’s protocol. A no-reverse transcription control was included in each run by omitting the reverse transcriptase.

Real-time quantitative PCR was performed using the CFX96™ Real-Time PCR Detection System (Bio-Rad, Hercules, CA, USA) and the FastStart Essential DNA Probes Master (Roche Diagnostics, Mannheim, Germany; cat. no. 06402682001), following the manufacturer’s instructions. Each reaction was carried out in duplicate using 2 μL of cDNA in a final volume of 10 μL. A melting curve analysis was performed at the end of each run to verify amplification specificity. No-reverse transcription controls were included to ensure that amplifications originated from mRNA and not from genomic DNA. Amplicons were characterized according to their melting temperature and expected size.

Relative mRNA expression levels of each target gene were calculated using the comparative cycle threshold (2^−ΔΔCq^) method and normalized against *CYC* (Cyclophilin) for tissue samples or *HPRT* (Hypoxanthine-guanine phosphoribosyltransferase) for cell line experiments [18,52]. Primer sequences for each target gene are listed in Supplementary Materials Table S3.

### 4.9. Indirect Immunofluorescence Assay

For morphological analysis, tumor cells (T47D and MDA-MB-231) were seeded on glass coverslips placed in 24-well plates and cultured until 70–80% confluence. After 24 h of treatment with *normal*-, *adjacent*-, or *control*-CM (D-MEM/F-12:M199, 1:1, 1% BSA final), cells were washed twice with PBS and fixed with 4% paraformaldehyde for 15 min at room temperature. Fixed cells were permeabilized with 0.1% Triton X-100 in PBS for 15 min and incubated with Alexa Fluor™ 488-conjugated phalloidin (Thermo Fisher Scientific, Waltham, MA, USA; cat. no. A12379) for 40 min at room temperature to visualize filamentous actin (F-actin). Nuclei were counterstained with Hoechst 33,342 (Thermo Fisher Scientific, Waltham, MA, USA; cat. no. H3570) for 10 min. Coverslips were mounted with VECTASHIELD^®^ Antifade Mounting Medium (Vector Laboratories, Burlingame, CA, USA; cat. no. H-1000). Images were acquired with a confocal fluorescence microscope (Olympus DSU IX83, Tokyo, Japan) using 20× and 60× oil immersion objectives.

### 4.10. Cell Proliferation Assay

To assess the effect of CM on cell proliferation, T47D and MDA-MB-231 cells (5000 cells/well) were seeded into 96-well plates and allowed to adhere overnight. The following day, cells were incubated for 24 h with *normal*-, *adjacent*, *control*-CM, each diluted 1:1 in D-MEM/F-12 containing 2% BSA (final concentration 1% BSA). Cell viability was determined using the MTS assay (CellTiter 96^®^ AQueous One Solution Cell Proliferation Assay, Promega, Madison, WI, USA) according to the manufacturer’s instructions. Absorbance at 490 nm was recorded after 1, 2, and 3 h of reagent incubation, and the corresponding blank value was subtracted from each reading. Since cell morphology was altered after 4 h, only data from the 3 h time point were analyzed for T47D cells, whereas the 1 h incubation time was used for MDA-MB-231 cells. Results are expressed as percentages relative to cells treated with *control*-CM (D-MEM/F-12:M199, 1:1, 1% BSA final).

### 4.11. Adhesion Assay

The adhesive capacity of tumor cells (T47D and MDA-MB-231) was assessed using 96-well plates pre-treated with undiluted CM (*normal*- or *adjacent*-CM) for 24 h. Wells were then washed with PBS and blocked for 1 h with PBS containing 1% BSA. After additional PBS washes, 50,000 cells/well were seeded in serum-free D-MEM/F-12. After 1 h of incubation, non-adherent cells were removed by aspiration, and wells were gently washed with PBS to eliminate residual loosely bound cells. Adherent cells were quantified using the MTS assay (CellTiter 96^®^ AQueous One Solution Cell Proliferation Assay, Promega, Madison, WI, USA), following the manufacturer’s instructions. Absorbance at 490 nm was measured after 1, 2, and 3 h of reagent incubation, and blank values were subtracted from each measurement. Because MTS signal intensity is proportional to the number of metabolically active adherent cells, the absorbance value at the selected time point was as a quantitative proxy of cell adhesion. For each experiment, raw absorbance values from CM-treated wells were normalized to the absorbance of control-CM (M199-treated) wells, which were processed in parallel and subjected to identical washing and incubation steps. To ensure that the absorbance signal reflected only adherent cells and not cell proliferation, the time point used for analysis was selected based on morphology and absorbance kinetics: 3 h for T47D and 1 h for MDA-MB-231 cells, as these conditions provided a linear signal range without evidence of morphological alterations or overgrowth. Results were expressed as percentages relative to wells treated with *control*-CM.

### 4.12. Migration Assay (Wound Healing)

Cell migration was evaluated using the IncuCyte^®^ live-cell imaging system (IncuCyte S3, Sartorius), and the Scratch Wound Analysis Module. T47D and MDA-MB-231 cells were seeded into 96-well ImageLock plates (Sartorius), which are specifically designed to ensure consistent wound geometry. T47D cells were plated at 60,000 cells/well and reached confluence after 24 h. MDA-MB-231 cells were plated at 30,000 cells/well and serum-starved overnight in D-MEM/F-12 containing 1% BSA to reduce proliferation-related contribution to wound closure. A standardized scratch wound was generated using the IncuCyte^®^ WoundMaker tool, which simultaneously produces uniform and reproducible wound widths across the entire plate. After scratching, wells were washed three times with PBS to remove cell debris and unattached cells. Subsequently, 100 μL of *normal*-, *adjacent*-, or *control*-CM (each diluted 1:1 in D-MEM/F-12 supplemented with 2% BSA; final 1% BSA) were added to each well. Plates were transferred to the IncuCyte^®^ system, and phase-contrast images were automatically acquired every 2 h for 48 h using a 10× objective. Acquisition settings (focus, exposure, contrast) were kept constant across all wells and experiments. Image segmentation was performed automatically using the Scratch Wound Module. The software first defined the wound region at time 0 through a predefined contrast-based mask, and migration was quantified as relative wound density (RWD) (%)—representing the cell density within the wound region relative to the surrounding monolayer. RWD is calculated as:

$$\%\;RWD\left(t\right)=\;100\cdot \frac{\left(w\left(t\right)-w(0)\right)}{\left(c\left(t\right)-w(0)\right)}$$

*w*(*t*) = Density of wound region at time, (*t*)

*c*(*t*) = Density of cell-covered region at time, (*t*)

By definition, RWD is 0% at the initial time point, as the metric accounts for the background density of the wound area at t = 0 and for dynamic changes in both wound and non-wound regions. This parameter is particularly suitable for breast cancer models, where treatments can alter cell morphology during migration. RWD values were exported from IncuCyte^®^ and plotted at 4 h intervals. Wells with irregular or poorly-formed wounds were excluded prior to analysis.

### 4.13. Comparative Bioinformatics Approach to Gene Expression in Breast Cancer Cell Line Models

A comparative bioinformatics analysis was performed using the GSE139670 dataset from the NCBI Gene Expression Omnibus (GEO) database (https://www.ncbi.nlm.nih.gov/geo/) (accessed on 8 August 2023). This dataset includes gene-expression profiles from BC cell lines of different molecular subtypes (MCF7, T47D and MDA-MB-231). Differential expression analysis was conducted using the GEO2R tool, which applies the limma package-based moderated t-statistics to compute gene-expression differences across predefined groups. We specifically analyzed the expression of pluripotency and prognosis-related genes, which were interrogated through pairwise comparisons between the selected cell lines. Expression homogeneity across biological replicates was evaluated by inspecting sample clustering, variance distribution, and replicate concordance metrics provided within the GEO2R interface. Samples failing quality-control criteria—such as outlier clustering or anomalous signal distribution—were excluded prior to analysis. Genes on the microarray platform were annotated with RefSeq identifiers. To accurately match the proteins examined experimentally in our study with their corresponding gene entries in the dataset, we mapped RefSeq and protein identifiers using the UniProt database (https://www.uniprot.org/) (accessed on 8 August 2023). This ensured precise alignment between the experimental targets and the transcriptomic data extracted from GEO2R.

### 4.14. Statistical Analysis

All experiments were performed at least twice with consistent results. Data distribution and homogeneity of variances were assessed by the Shapiro-Wilk and Levene tests, respectively. When assumptions of normality and homoscedasticity were met, comparisons among groups were conducted using one-way ANOVA followed by Tukey’s post hoc test. Non-compliance with the homogeneity assumption was addressed by modeling the variance structure using *varIdent* or *vasPower* functions, selecting the optimal structure based on the lowest AIC among models that did not reject the homogeneity assumption. A *p*-value < 0.05 was considered statistically significant. All statistical analyses were performed using R software (version 4.3.2; R Foundation for Statistical Computing, Vienna, Austria, https://www.r-project.org/) (accessed on 1 September 2025) and RStudio (version 2023.09.1; Posit Software, PBC, Boston, MA, USA, https://www.posit.co/) (accessed on 1 September 2025), while graphical representations were generated with GraphPad Prism (version 9.0; GraphPad Software Inc, San Diego, CA, USA).

## 5. Conclusions

In conclusion, our findings support the notion that the influence of peritumoral AT is shaped by the intrinsic molecular programming of each BC subtype, rather than acting as a uniform driver of aggressiveness. The use of two hormone receptor-positive cell lines (MCF7 and T47D) allowed us to partially capture the heterogeneity within HR+ disease, revealing that adipose-derived cues can differentially modulate phenotypic programs even within the same molecular class. The early stromal alterations identified here—particularly those involving lipid metabolic remodeling and cytoskeletal dynamics (e.g., FABP4 and vimentin)—underscore the clinical relevance of adipose-derived signals as indicators of tumor–stroma interplay and as potential subtype-specific therapeutic targets.

Moreover, our preliminary observations suggesting a protective or antitumorigenic role of soluble factors derived from patients with TNBC are noteworthy. These results raise the possibility that specific components of the adipose secretome may attenuate pro-tumorigenic programs in this clinically challenging subtype. Identifying these mediators could uncover signaling cues capable of restraining disease progression or complementing current therapeutic strategies, which remain limited for TNBC.

This study also presents limitations. A key limitation lies in the TNBC model: because vimentin could not be evaluated in this experimental setting, our ability to fully assess EMT-related remodeling and its relationship with adipose-derived cues in TNBC was restricted. This gap limits the completeness of our subtype comparison and highlights the need for complementary TN models and additional functional validation in future studies.

Finally, although our functional assays provide insight into early cellular responses to adipose-derived cues, they were conducted exclusively in vitro. Thus, the mechanistic pathways underlying AT-tumor crosstalk remain to be fully elucidated. Future studies employing in vivo validation and targeted mechanistic analyses will be essential to define the signaling and metabolic circuits through which peritumoral AT shapes subtype-specific tumor behavior.

## Data Availability

The datasets underlying the results of this study are included in the article and its Supplementary Files and are available from the corresponding author upon reasonable request.

## Supplementary Materials

The following supporting information can be downloaded at: https://www.mdpi.com/article/10.3390/ijms27021129/s1.

## Abbreviations

The following abbreviations are used in this manuscript:

*adjacent*	adipose tissue adjacent to the tumor (<2 cm away from the tumor)
AT	adipose tissue
BC	breast cancer
CM	conditioned media
*control*-CM	control conditioned media
ECM	extracellular matrix
EMT	epithelial–mesenchymal transition
ER	estrogen receptor
*distant*	adipose tissue distant from the tumor (>2 cm away from the tumor)
FABP4	fatty acid binding protein 4
FBS	fetal bovine serum
HPRT	Hypoxanthine-guanine phosphoribosyltransferase
HR+	estrogen and progesterone receptor positive
*normal*-CM	conditioned media obtained from breast AT explants from women without cancer
PBS	phosphate-buffered saline
PR	progesterone receptor
qPCR	quantitative real-time PCR
TBS	tris-buffered saline
TNBC	triple-negative breast cancer
WAT	white adipose tissue
WB	western blot
