# Supplementary material for: Microenvironment Modulates Tumorigenicity of Breast Cancer Cells Depending on Hormone Receptor Status
Source: Int J Mol Sci. 2026 Jan 22;27(2):1129. doi: 10.3390/ijms27021129 (PMC12842586; doi:10.3390/ijms27021129)

Supplementary Figure S1. Expression levels of CD44 and MMP9 in the breast adipose microenvironment. Quantification of CD44, and MMP9 in *normal*, *adjacent* and *distant* breast AT explants. The values were normalized to the corresponding loading control and represented as violin plots, where the dashed line indicates the median and dotted lines represent the first and the third quartiles. AT explants used in WB assay: $n_{normal\text{-}AT} = 6$, $n_{adacentj\text{-}AT} = 9$ and $n_{distant\text{-}AT} = 8$ for CD44, and $n_{normal\text{-}AT} = 5$, $n_{adacentj\text{-}AT} = 6$ and $n_{distant\text{-}AT} = 6$ for MMP9. *Adjacent* breast explants (AT <2 cm from the tumor); *distant* breast explants (AT >2 cm from the tumor).

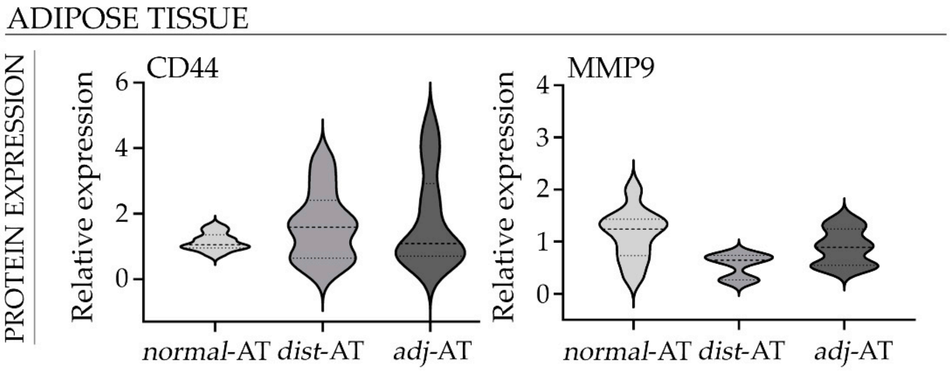

Supplement: Supplementary file 1 [file ijms-27-01129-s001.zip › Supplementary Figure S1.pdf]
