# Supplementary material for: Microenvironment Modulates Tumorigenicity of Breast Cancer Cells Depending on Hormone Receptor Status
Source: Int J Mol Sci. 2026 Jan 22;27(2):1129. doi: 10.3390/ijms27021129 (PMC12842586; doi:10.3390/ijms27021129)

**Supplementary Figure S2.** Effect of CM on the expression of pluripotency and/or prognosis markers in HR+ BC models. (**A**) Quantification of OCT4, and MMP9 in MCF7, and KLF4, and MMP9 in T47D cells incubated with *control*-CM, *normal*-CM and *adjacent*-CM. Quantification values were normalized to the corresponding loading control and represented as violin plots, where the dashed line indicates the median and dotted lines represent the first and the third quartiles. (**B**) The mRNA levels of *CAV1*, and *VIM* from MCF7 and T47D cells treated with the different CM were analyzed by RT-qPCR. Values were normalized to reference genes and represented as violin plots. CM used in the WB assays: $n_{\textit{normal}\text{-CM}} = 8$ and $n_{\textit{adacentj}\text{-CM}} = 10$ for MCF7 and $n_{\textit{normal}\text{-CM}} = 7$ and $n_{\textit{adjacent}\text{-CM}} = 10$ for T47D; CM used in the qPCR assays: $n_{\textit{normal}\text{-CM}} = 12$ and $n_{\textit{adacentj}\text{-CM}} = 13$ for MCF7 and $n_{\textit{normal}\text{-CM}} = 14$ and $n_{\textit{adjacent}\text{-CM}} = 13$ for T47D. *Adj*-CM, conditioned media from *adjacent* breast explants (AT <2 cm from the tumor); *control*-CM, control conditioned media; *normal*-CM, conditioned media from human healthy breast adipose tissue explants.

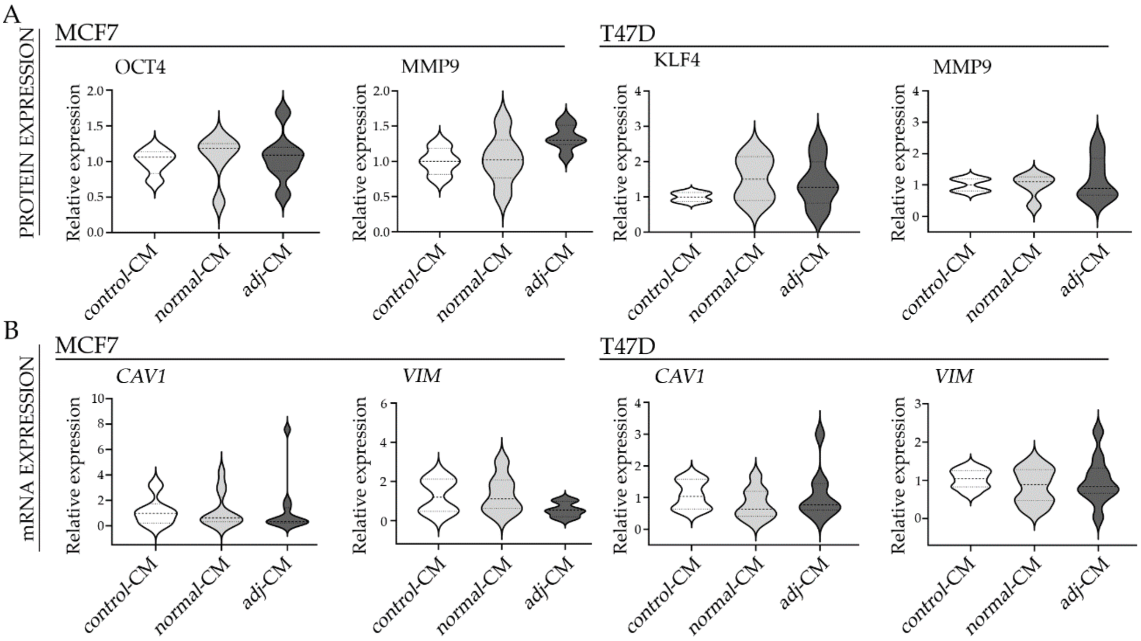

Supplement: Supplementary file 1 [file ijms-27-01129-s001.zip › Supplementary Figure S2.pdf]
