# Supplementary material for: Microenvironment Modulates Tumorigenicity of Breast Cancer Cells Depending on Hormone Receptor Status
Source: Int J Mol Sci. 2026 Jan 22;27(2):1129. doi: 10.3390/ijms27021129 (PMC12842586; doi:10.3390/ijms27021129)
Supplement: Supplementary file 1 [file ijms-27-01129-s001.zip › Supplementary Figure S3.pdf]

Supplementary Figure S3. Original Western blot images showing protein expression in human breast adipose tissue explants. Actin was used as a loading control. Each condition was loaded in duplicate.

Arrows indicate the specific protein bands that were quantified: caveolin-1 doublet (~21–24 kDa), FABP4 band (~15 kDa), adiponectin band (~30 kDa), vimentin doublet (~58 kDa), CD44 band (~80 kDa), MMP9 bands (~100–150 kDa), and actin band (~43 kDa). Lanes labeled with a number followed by "A" (*adjacent*) or "D" (*distant*) correspond to protein lysates from adipose tissue samples collected from sites adjacent to (<2 cm) or distant (>2 cm) from the tumor, respectively. Lanes labeled with bold letters represent protein lysates from adipose tissue collected from healthy individuals (*normal*). Lanes marked with an asterisk (*) indicate samples excluded from quantitative analysis because they did not meet the predefined quality criteria (appropriate molecular weight, specific signal, and acceptable membrane integrity). Caveolin-1 and FABP4 detection was performed on eight independent blots (blots 1–8), adiponectin and vimentin on eight independent blots (blots 21–28), MMP9 on four independent blots (blots 17–20), and CD44 on four independent blots (blots 33–36). Actin detection was performed on all blots (1–8, 17–20, 21–28, and 33–36) and used as a loading control. Cav-1, caveolin-1; MWM, molecular weight markers.

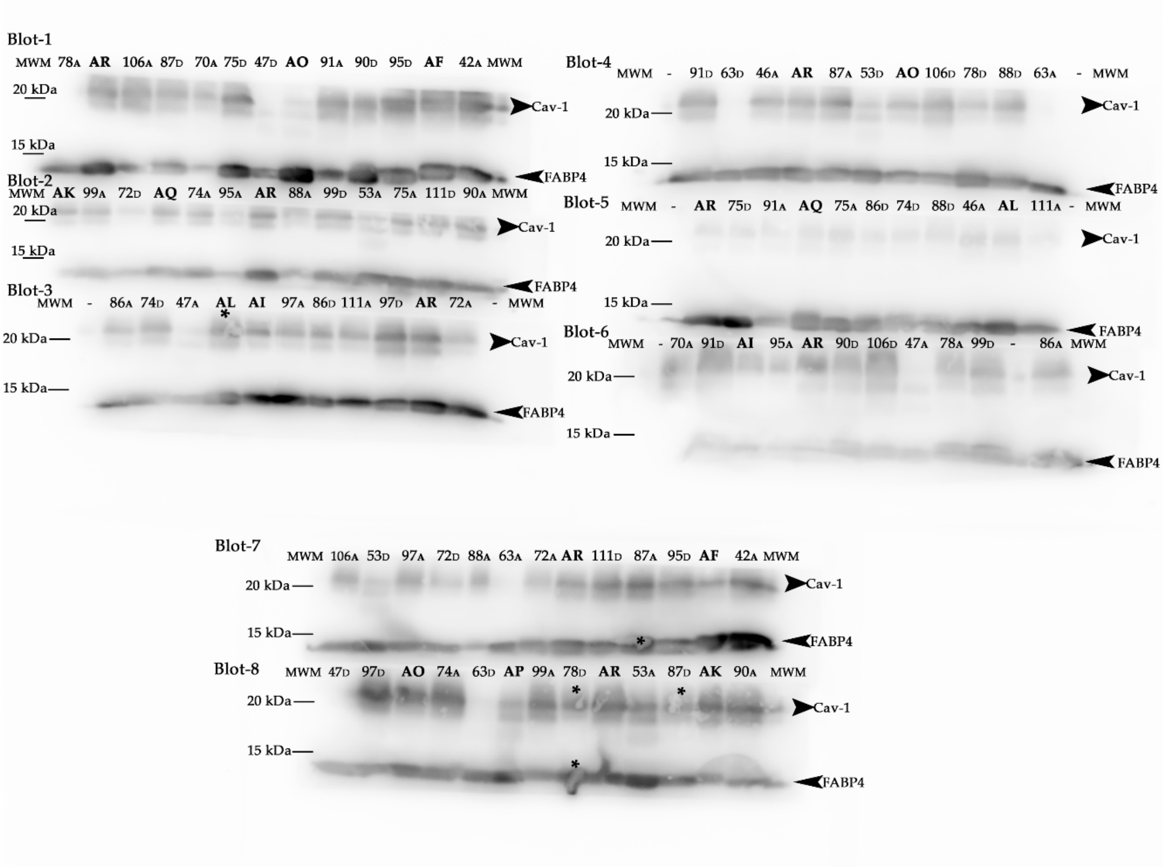

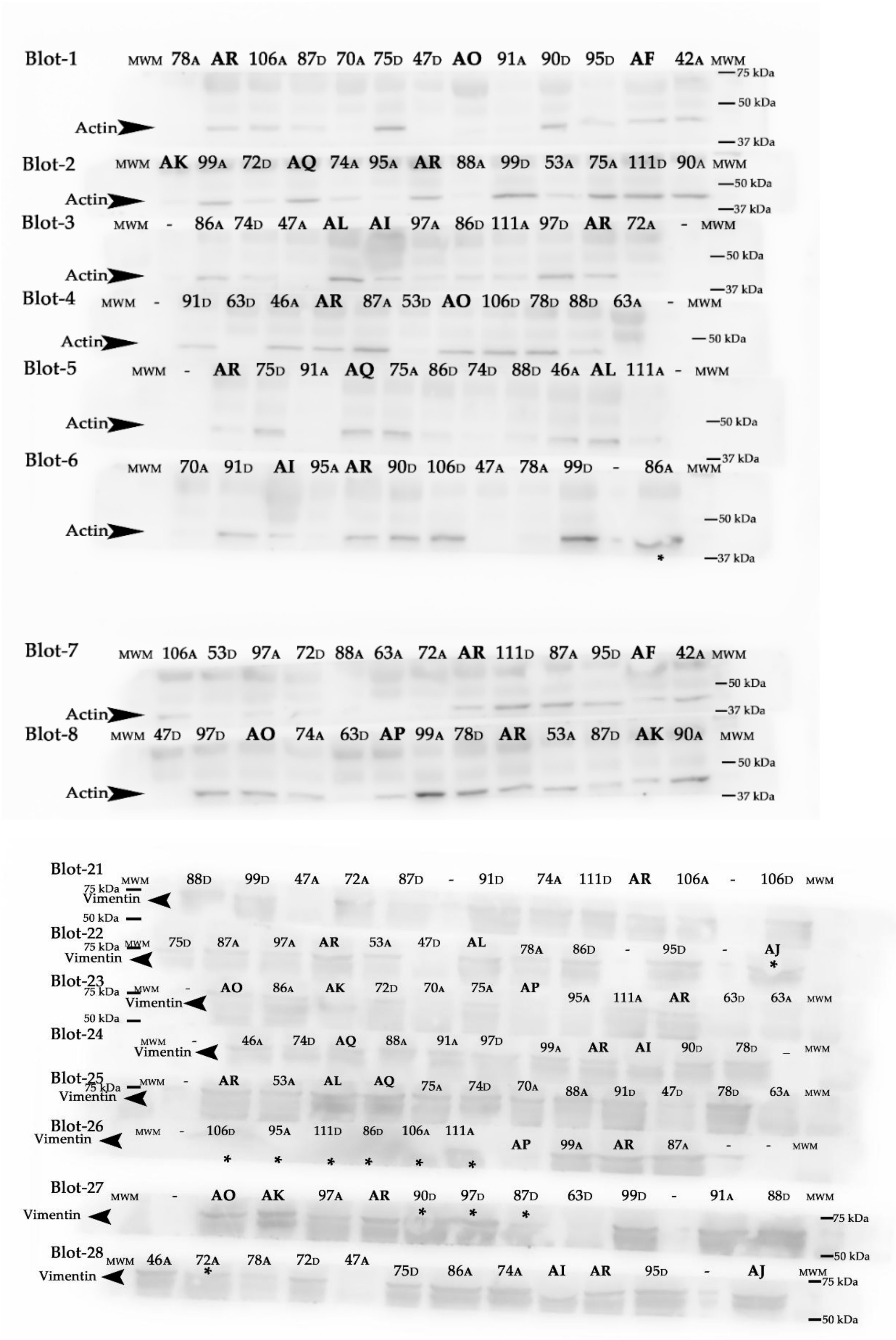

Blot-1 MWM 78A AR 106A 87D 70A 75D 47D AO 91A 90D 95D AF 42A MWM
75 kDa
50 kDa
Actin
37 kDa
Blot-2 MWM AK 99A 72D AQ 74A 95A AR 88A 99D 53A 75A 111D 90A MWM
50 kDa
Actin
37 kDa
Blot-3 MWM - 86A 74D 47A AL AI 97A 86D 111A 97D AR 72A - MWM
50 kDa
Actin
37 kDa
Blot-4 MWM - 91D 63D 46A AR 87A 53D AO 106D 78D 88D 63A - MWM
Actin
50 kDa
Blot-5 MWM - AR 75D 91A AQ 75A 86D 74D 88D 46A AL 111A - MWM
Actin
50 kDa
37 kDa
Blot-6 MWM 70A 91D AI 95A AR 90D 106D 47A 78A 99D - 86A MWM
50 kDa
Actin
*
37 kDa
Blot-7 MWM 106A 53D 97A 72D 88A 63A 72A AR 111D 87A 95D AF 42A MWM
50 kDa
Actin
37 kDa
Blot-8 MWM 47D 97D AO 74A 63D AP 99A 78D AR 53A 87D AK 90A MWM
50 kDa
Actin
37 kDa
Blot-21 MWM 88D 99D 47A 72A 87D - 91D 74A 111D AR 106A - 106D MWM
75 kDa
Vimentin
50 kDa
Blot-22 75 kDa MWM 75D 87A 97A AR 53A 47D AL 78A 86D - 95D - AJ
Vimentin
*
Blot-23 75 kDa MWM - AO 86A AK 72D 70A 75A AP 95A 111A AR 63D 63A MWM
Vimentin
50 kDa
Blot-24 MWM - 46A 74D AQ 88A 91A 97D 99A AR AI 90D 78D _ MWM
Vimentin
Blot-25 75 kDa MWM - AR 53A AL AQ 75A 74D 70A 88A 91D 47D 78D 63A MWM
Vimentin
Blot-26 MWM - 106D 95A 111D 86D 106A 111A AP 99A AR 87A - - MWM
Vimentin
* * * * * *
Blot-27 MWM - AO AK 97A AR 90D 97D 87D 63D 99D - 91A 88D MWM
Vimentin
* * *
75 kDa
50 kDa
Blot-28 MWM 46A 72A 78A 72D 47A 75D 86A 74A AI AR 95D - AJ MWM
Vimentin
*
75 kDa
50 kDa

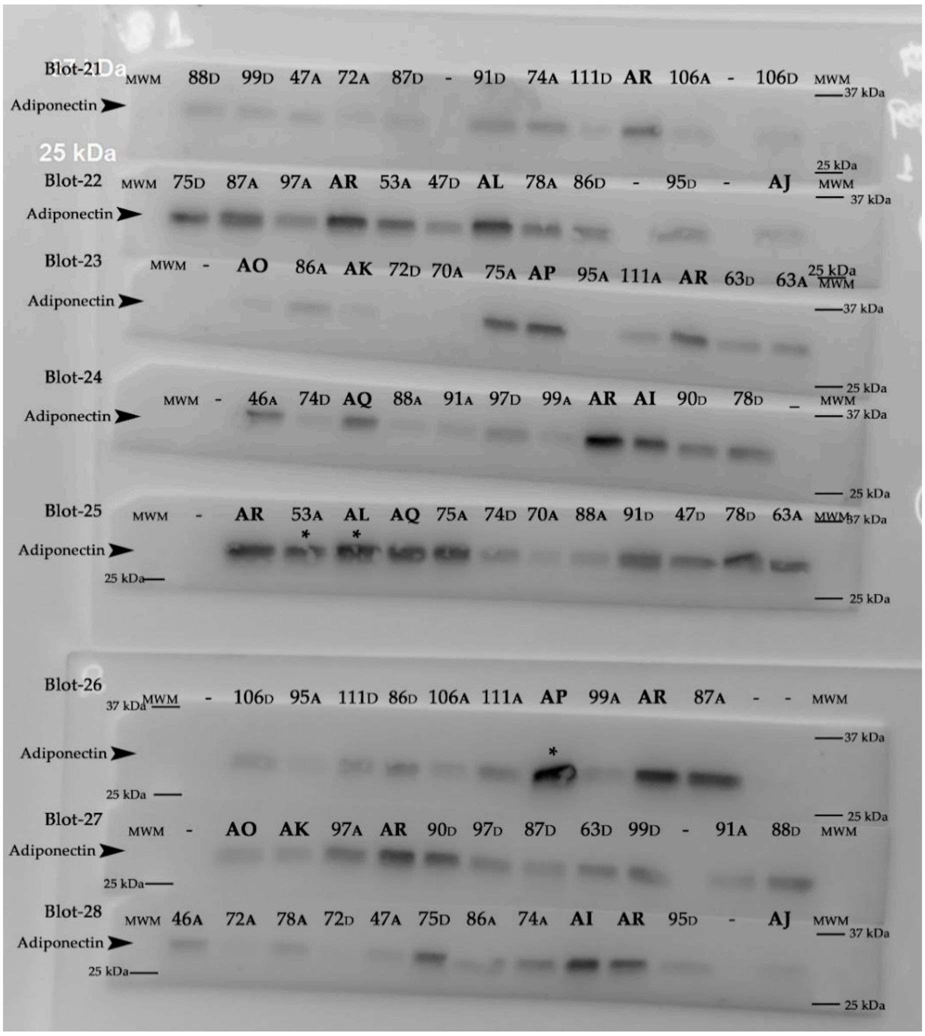
Blot-21 MWM 88D 99D 47A 72A 87D - 91D 74A 111D AR 106A - 106D MWM
Adiponectin
Blot-22 MWM 75D 87A 97A AR 53A 47D AL 78A 86D - 95D - AJ MWM
Blot-23 MWM - AO 86A AK 72D 70A 75A AP 95A 111A AR 63D 63A MWM
Blot-24 MWM - 46A 74D AQ 88A 91A 97D 99A AR AI 90D 78D - MWM
Blot-25 MWM - AR 53A AL AQ 75A 74D 70A 88A 91D 47D 78D 63A MWM
Blot-26 MWM - 106D 95A 111D 86D 106A 111A AP 99A AR 87A - - MWM
Blot-27 MWM - AO AK 97A AR 90D 97D 87D 63D 99D - 91A 88D MWM
Blot-28 MWM 46A 72A 78A 72D 47A 75D 86A 74A AI AR 95D - AJ MWM
37 kDa
25 kDa

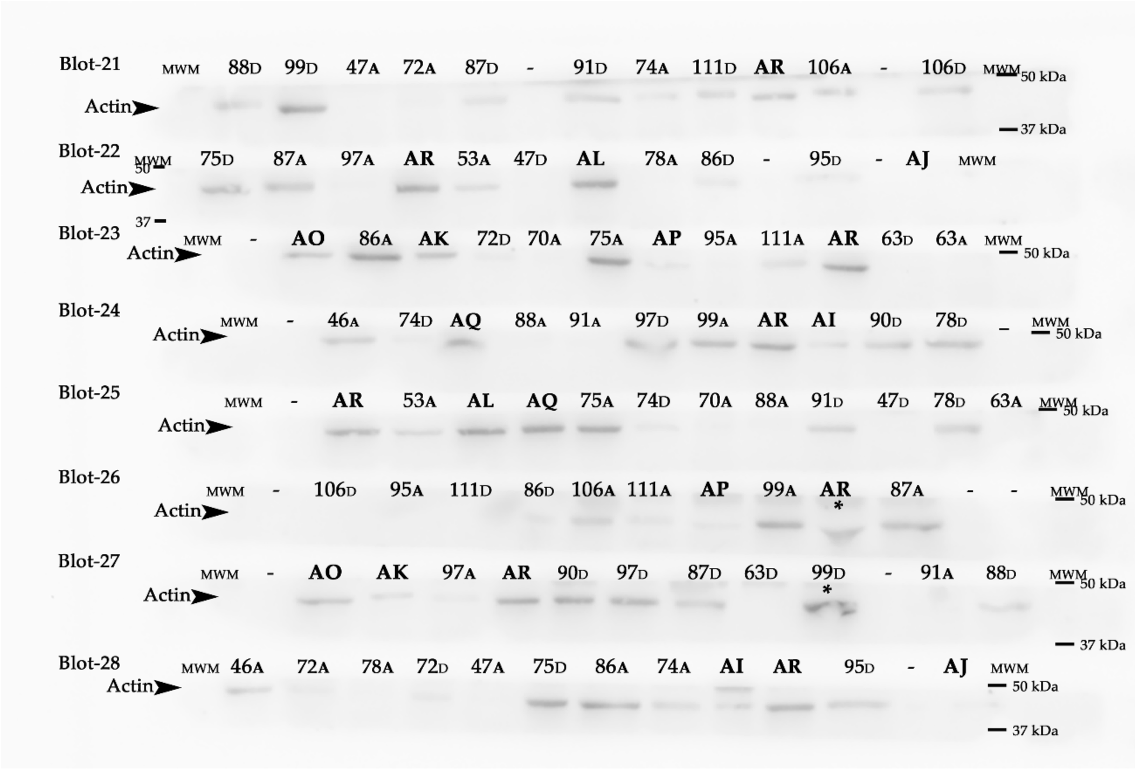
Blot-21 MWM 88D 99D 47A 72A 87D - 91D 74A 111D AR 106A - 106D MWM
Actin
Blot-22 MWM 75D 87A 97A AR 53A 47D AL 78A 86D - 95D - AJ MWM
Blot-23 MWM - AO 86A AK 72D 70A 75A AP 95A 111A AR 63D 63A MWM
Blot-24 MWM - 46A 74D AQ 88A 91A 97D 99A AR AI 90D 78D - MWM
Blot-25 MWM - AR 53A AL AQ 75A 74D 70A 88A 91D 47D 78D 63A MWM
Blot-26 MWM - 106D 95A 111D 86D 106A 111A AP 99A AR 87A - - MWM
Blot-27 MWM - AO AK 97A AR 90D 97D 87D 63D 99D - 91A 88D MWM
Blot-28 MWM 46A 72A 78A 72D 47A 75D 86A 74A AI AR 95D - AJ MWM
50 kDa
37 kDa

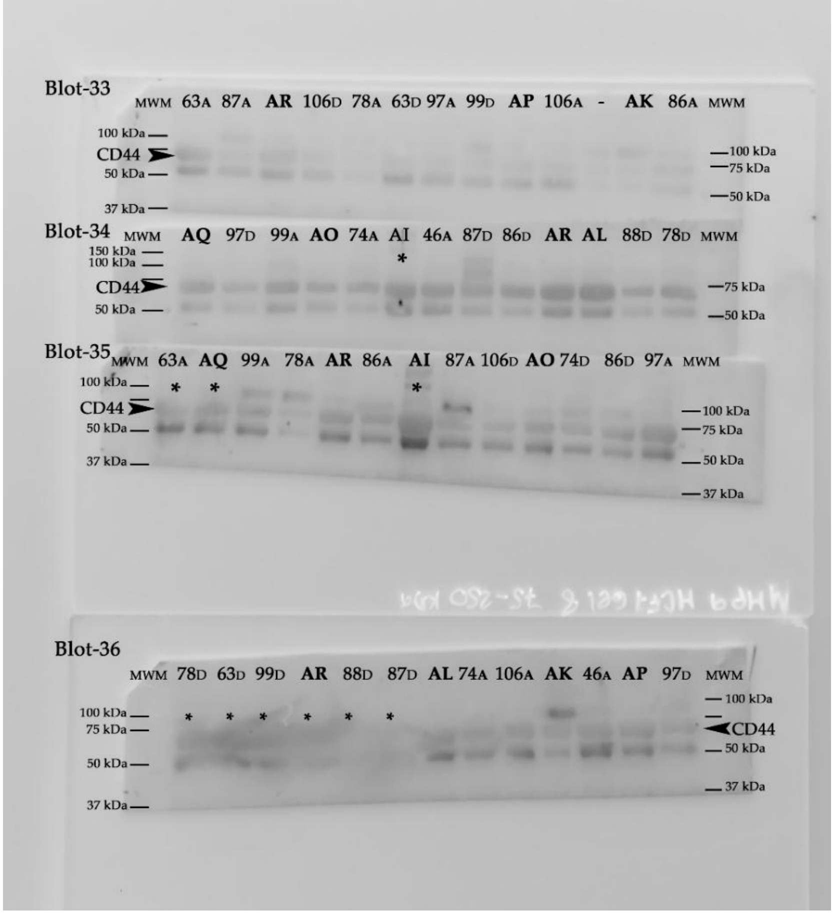
Blot-33
MWM 63A 87A AR 106D 78A 63D 97A 99D AP 106A - AK 86A MWM
100 kDa
CD44
50 kDa
37 kDa
100 kDa
75 kDa
50 kDa
Blot-34
MWM AQ 97D 99A AO 74A AI 46A 87D 86D AR AL 88D 78D MWM
150 kDa
100 kDa
CD44
50 kDa
75 kDa
50 kDa
Blot-35
MWM 63A AQ 99A 78A AR 86A AI 87A 106D AO 74D 86D 97A MWM
100 kDa
CD44
50 kDa
37 kDa
100 kDa
75 kDa
50 kDa
37 kDa
Blot-36
MWM 78D 63D 99D AR 88D 87D AL 74A 106A AK 46A AP 97D MWM
100 kDa
75 kDa
50 kDa
37 kDa
100 kDa
CD44
50 kDa
37 kDa

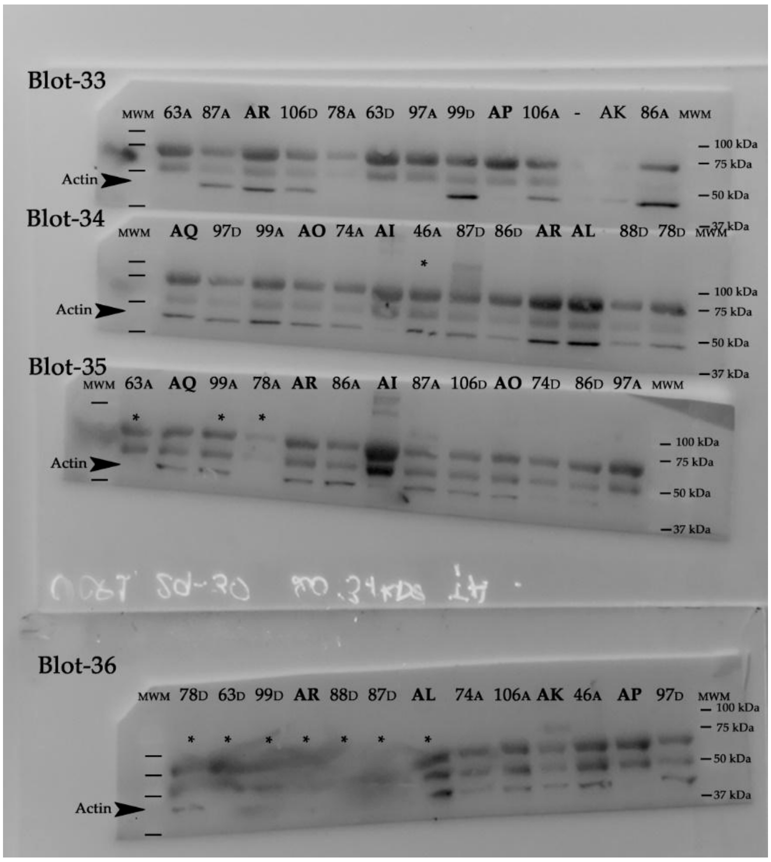
Blot-33
MWM 63A 87A AR 106D 78A 63D 97A 99D AP 106A - AK 86A MWM
Actin
100 kDa
75 kDa
50 kDa
37 kDa
Blot-34
MWM AQ 97D 99A AO 74A AI 46A 87D 86D AR AL 88D 78D MWM
Actin
100 kDa
75 kDa
50 kDa
37 kDa
Blot-35
MWM 63A AQ 99A 78A AR 86A AI 87A 106D AO 74D 86D 97A MWM
Actin
100 kDa
75 kDa
50 kDa
37 kDa
Blot-36
MWM 78D 63D 99D AR 88D 87D AL 74A 106A AK 46A AP 97D MWM
Actin
100 kDa
75 kDa
50 kDa
37 kDa

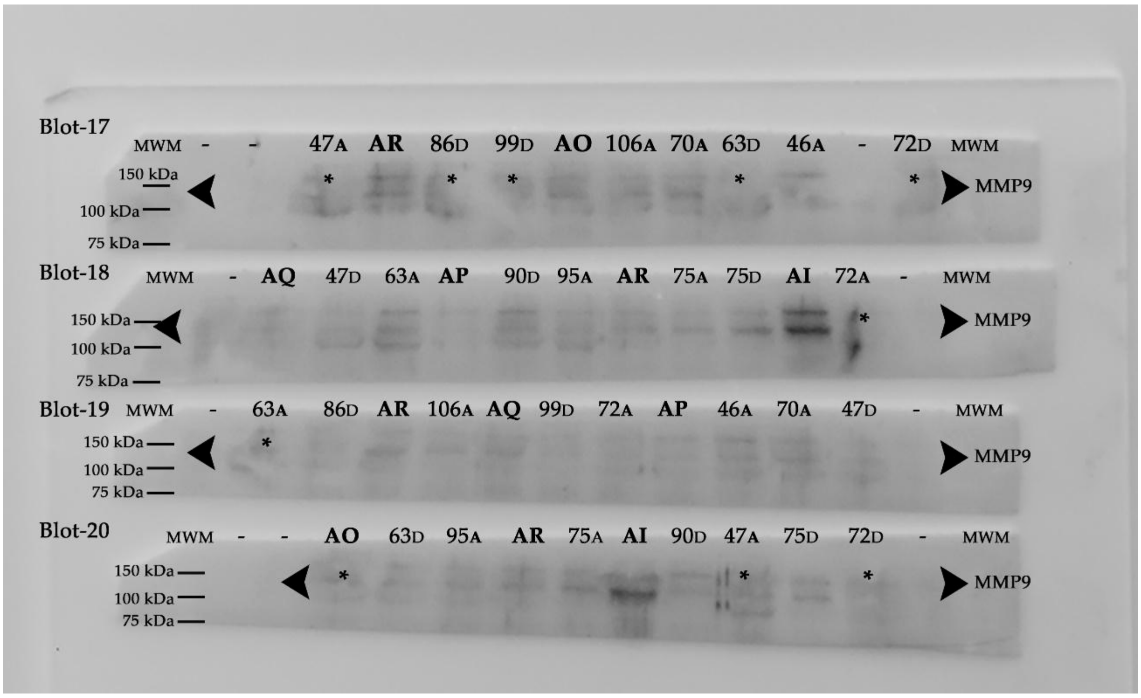
Blot-17
MWM - - 47A AR 86D 99D AO 106A 70A 63D 46A - 72D MWM
150 kDa
100 kDa
75 kDa
MMP9
Blot-18
MWM - AQ 47D 63A AP 90D 95A AR 75A 75D AI 72A - MWM
150 kDa
100 kDa
75 kDa
MMP9
Blot-19
MWM - 63A 86D AR 106A AQ 99D 72A AP 46A 70A 47D - MWM
150 kDa
100 kDa
75 kDa
MMP9
Blot-20
MWM - - AO 63D 95A AR 75A AI 90D 47A 75D 72D - MWM
150 kDa
100 kDa
75 kDa
MMP9

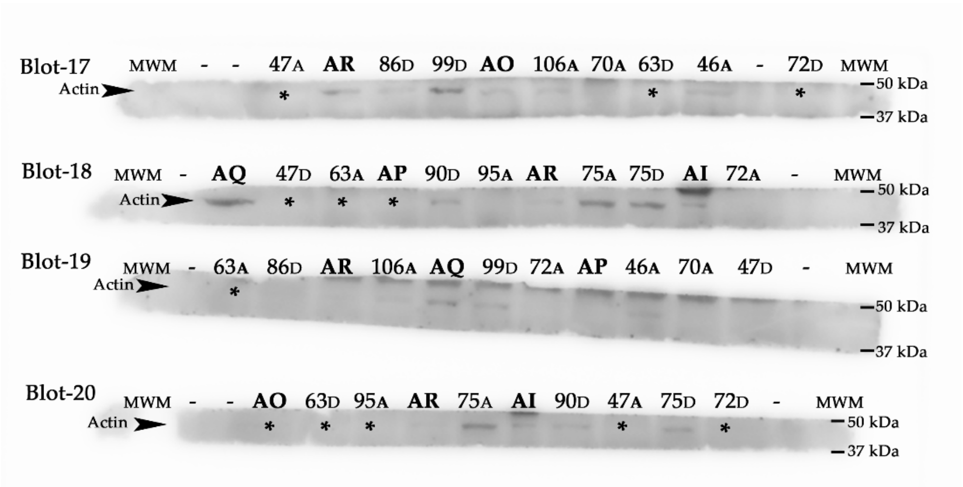
Blot-17
MWM - - 47A AR 86D 99D AO 106A 70A 63D 46A - 72D MWM
Actin
50 kDa
37 kDa
Blot-18
MWM - AQ 47D 63A AP 90D 95A AR 75A 75D AI 72A - MWM
Actin
50 kDa
37 kDa
Blot-19
MWM - 63A 86D AR 106A AQ 99D 72A AP 46A 70A 47D - MWM
Actin
50 kDa
37 kDa
Blot-20
MWM - - AO 63D 95A AR 75A AI 90D 47A 75D 72D - MWM
Actin
50 kDa
37 kDa
