# Supplementary material for: Microenvironment Modulates Tumorigenicity of Breast Cancer Cells Depending on Hormone Receptor Status
Source: Int J Mol Sci. 2026 Jan 22;27(2):1129. doi: 10.3390/ijms27021129 (PMC12842586; doi:10.3390/ijms27021129)
Supplement: Supplementary file 1 [file ijms-27-01129-s001.zip › Supplementary Figure S4.pdf]

Supplementary Figure S4. Original Western blot images showing protein expression of pluripotency and prognosis-related markers (SOX2, Nanog, OCT4, KLF4, caveolin-1, CD44, vimentin, and MMP9) in MCF7 tumor cells treated with different conditioned media (*control*-, *normal*-, and *adj*-CM). Actin or β-tubulin served as loading controls.

Arrows indicate the specific protein bands that were quantified: caveolin-1 doublet (~21–24 kDa), KLF4 band (~53 kDa), SOX2 band (~34 kDa), Nanog band (~40 kDa), OCT4 band (~45 kDa), vimentin doublet (~58 kDa), CD44 band (~80 kDa), MMP9 bands (~100-150 kDa), actin band (~43 kDa), and β-Tubulin (~55 kDa). Caveolin-1 detection was performed on four independent blots (blots 5, 6, 9, and 10); KLF4 detection, on two independent blots (blots 6 and 9); SOX2 detection, on three independent blots (blots 6, 9, and 11); Nanog detection, on three independent blots (blots 7, 10, and 11); OCT4 detection, on two independent blots (blots 6 and 8); vimentin detection, on two independent blots (blots 6 and 11); CD44 detection, on three independent blots (blots 7, 10, and 11); MMP9 detection, on two independent blots (blots 5 and 8); actin detection, on the following blots: 5, 6, 8, 9, and 11; and β-Tubulin detection, on the following blots: 7, 10, and 11. MWM, molecular weight markers.

Lanes labeled with a number correspond to protein lysates from MCF7 treated with *adjacent*-CM; Lanes labeled with different letters represent protein lysates from MCF7 treated with *normal*-CM; Lanes labeled with C1-3 represent protein lysates from MCF7 treated with *control*-CM.

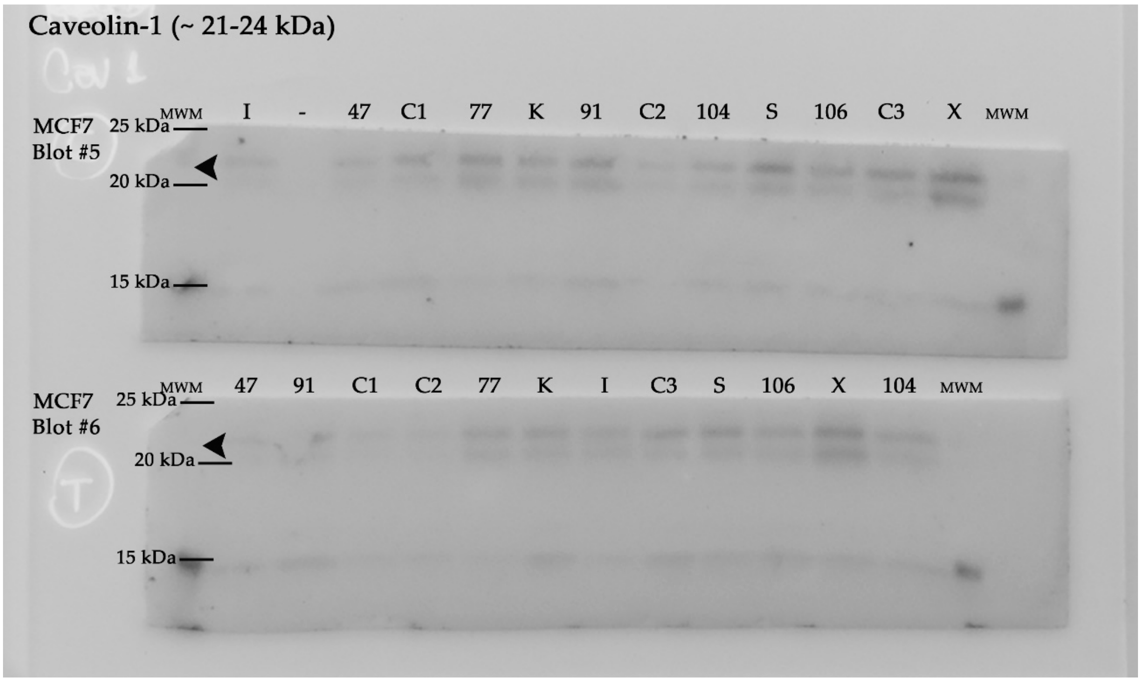

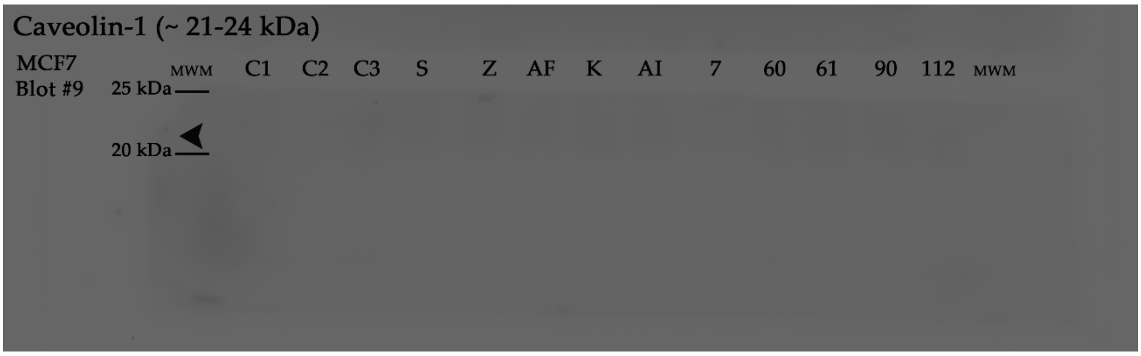

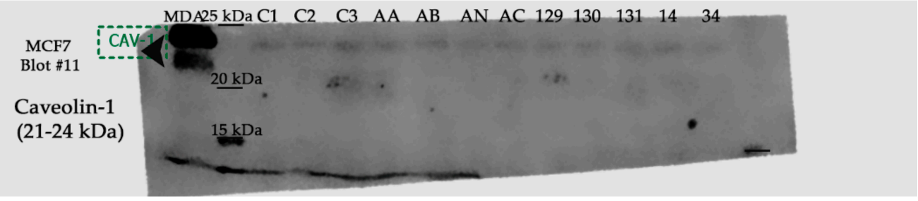

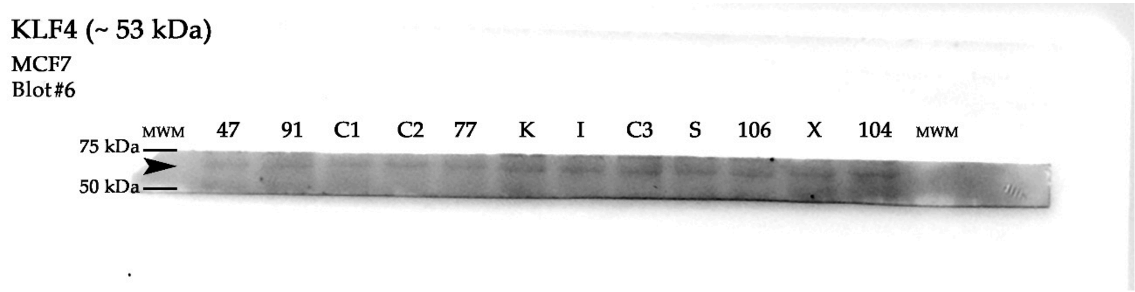
KLF4 (~ 53 kDa)
MCF7
Blot #6
MWM 47 91 C1 C2 77 K I C3 S 106 X 104 MWM
75 kDa
50 kDa

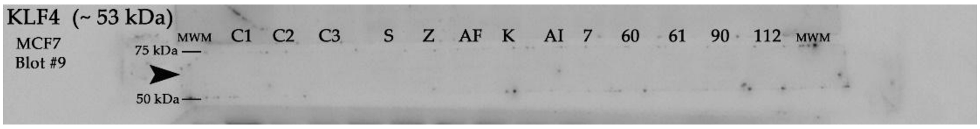
KLF4 (~ 53 kDa)
MCF7
Blot #9
MWM C1 C2 C3 S Z AF K AI 7 60 61 90 112 MWM
75 kDa
50 kDa

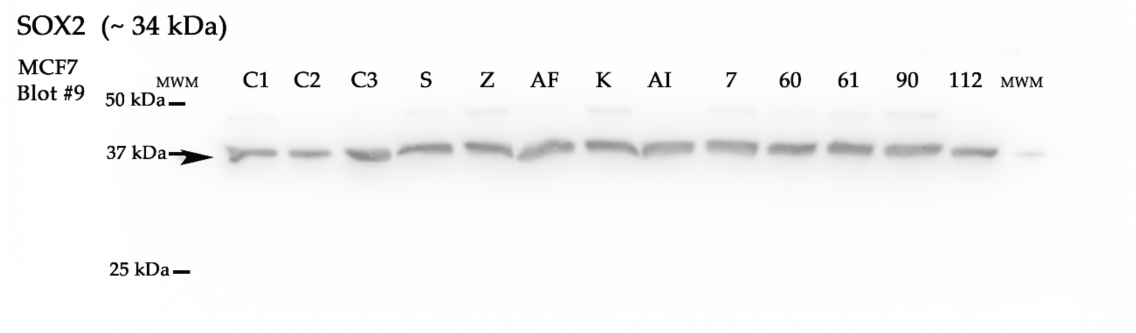
SOX2 (~ 34 kDa)
MCF7
Blot #9
MWM C1 C2 C3 S Z AF K AI 7 60 61 90 112 MWM
50 kDa
37 kDa
25 kDa

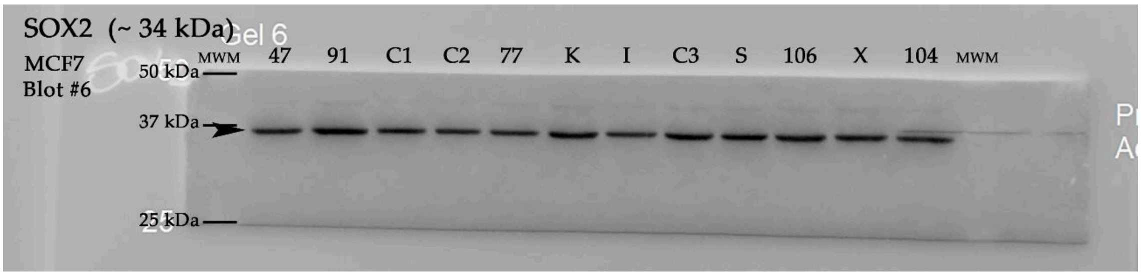
SOX2 (~ 34 kDa)
MCF7
Blot #6
MWM 47 91 C1 C2 77 K I C3 S 106 X 104 MWM
50 kDa
37 kDa
25 kDa

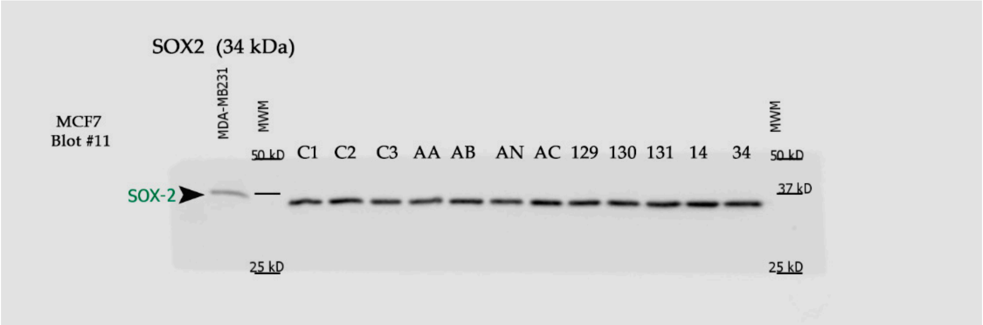
SOX2 (34 kDa)
MCF7
Blot #11
MDA-MB231
MWM
50 kD C1 C2 C3 AA AB AN AC 129 130 131 14 34
MWM
50 kD
37 kD
SOX-2
25 kD
25 kD

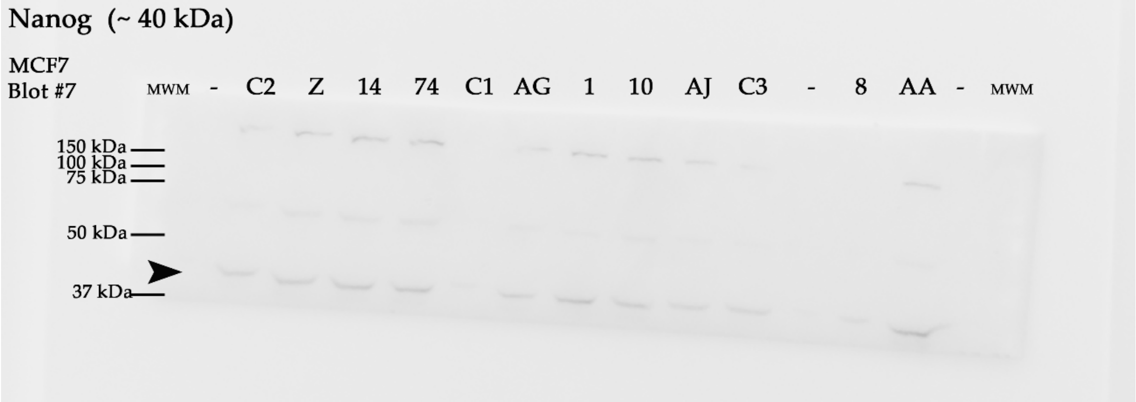
Nanog (~ 40 kDa)
MCF7
Blot #7
MWM - C2 Z 14 74 C1 AG 1 10 AJ C3 - 8 AA - MWM
150 kDa
100 kDa
75 kDa
50 kDa
37 kDa

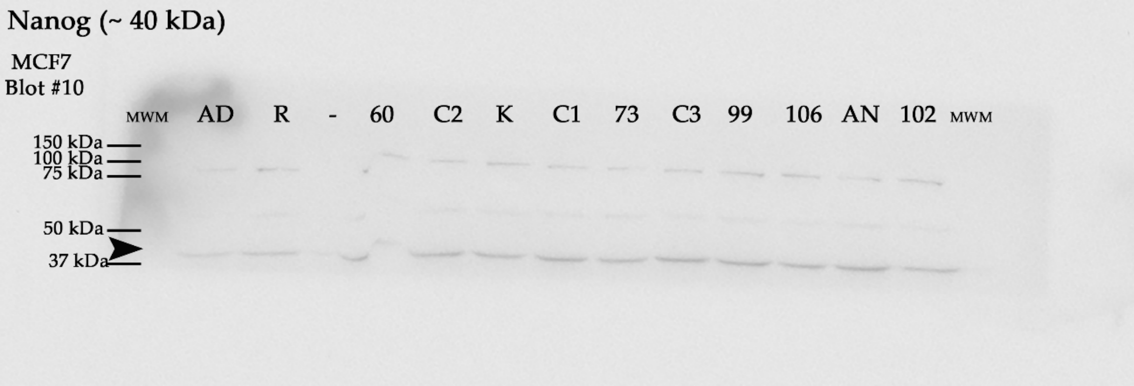
Nanog (~ 40 kDa)
MCF7
Blot #10
MWM AD R - 60 C2 K C1 73 C3 99 106 AN 102 MWM
150 kDa
100 kDa
75 kDa
50 kDa
37 kDa

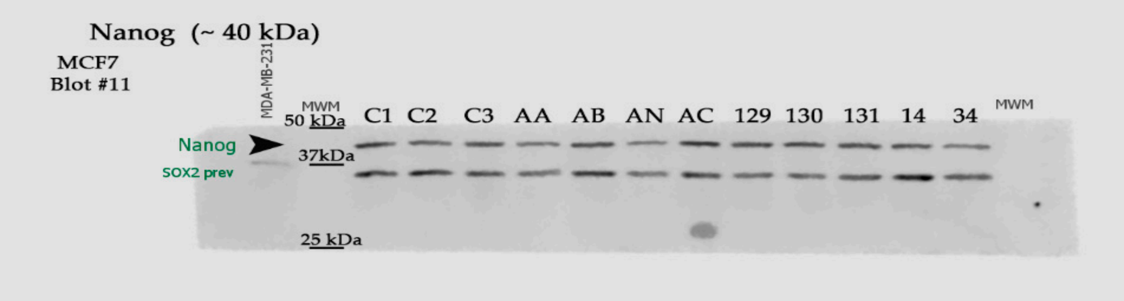
Nanog (~ 40 kDa)
MCF7
Blot #11
MDA-MB-231
MWM
50 kDa
C1 C2 C3 AA AB AN AC 129 130 131 14 34
MWM
Nanog
SOX2 prev
37kDa
25 kDa

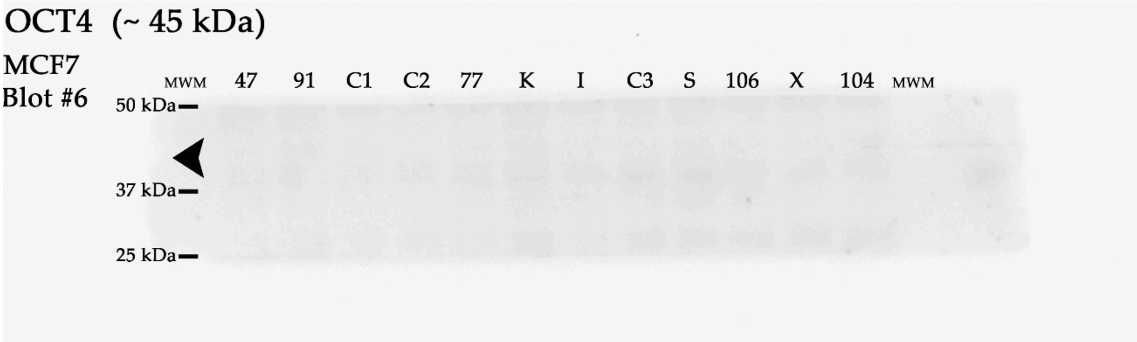
OCT4 (~ 45 kDa)
MCF7
Blot #6
MWM 47 91 C1 C2 77 K I C3 S 106 X 104 MWM
50 kDa
37 kDa
25 kDa

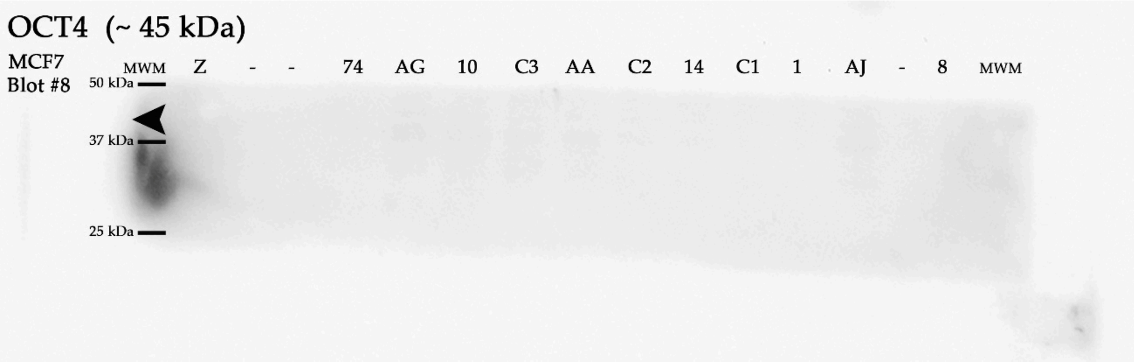
OCT4 (~ 45 kDa)
MCF7
Blot #8
MWM Z - - 74 AG 10 C3 AA C2 14 C1 1 AJ - 8 MWM
50 kDa
37 kDa
25 kDa

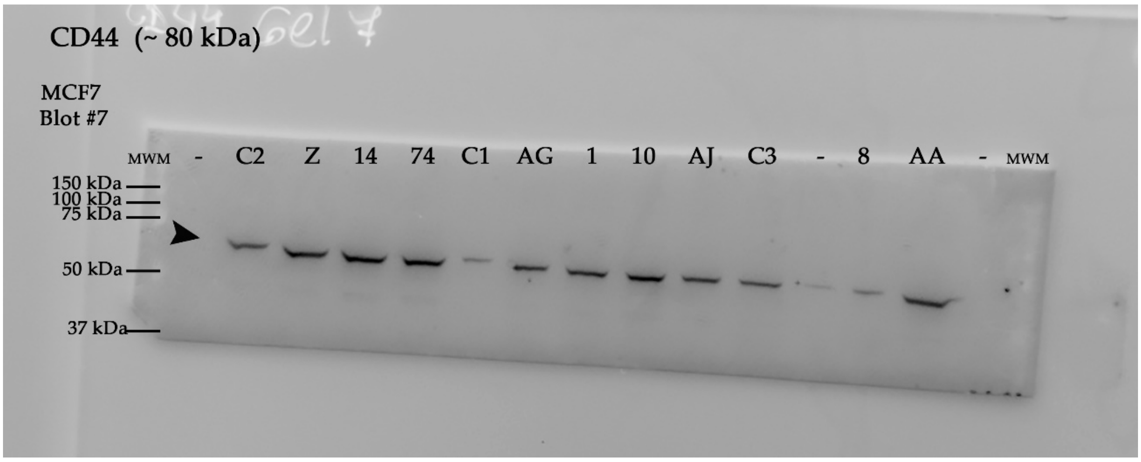
CD44 (~ 80 kDa)
MCF7
Blot #7
MWM - C2 Z 14 74 C1 AG 1 10 AJ C3 - 8 AA - MWM
150 kDa
100 kDa
75 kDa
50 kDa
37 kDa

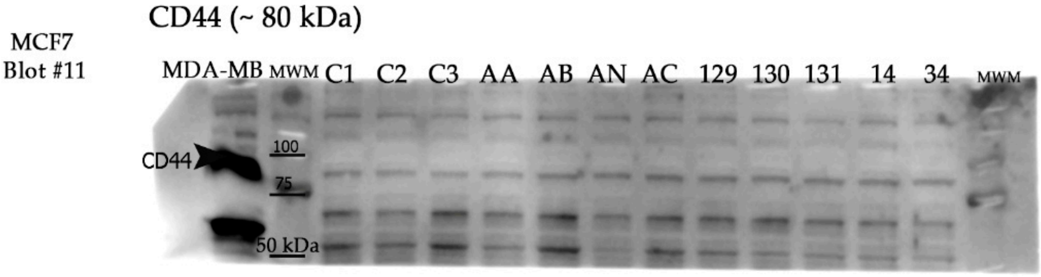
CD44 (~ 80 kDa)
MCF7
Blot #11
MDA-MB MWM C1 C2 C3 AA AB AN AC 129 130 131 14 34 MWM
CD44
100
75
50 kDa

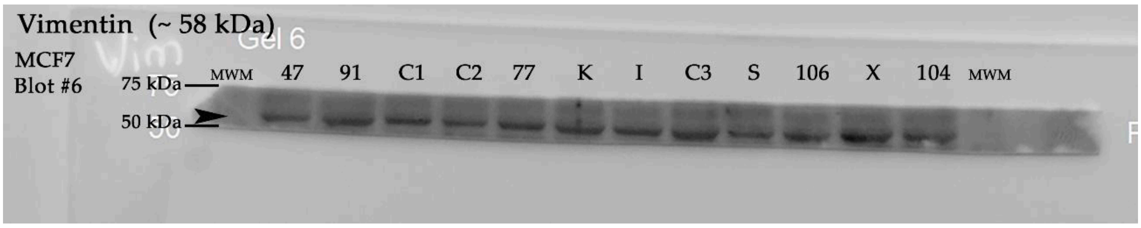
Vimentin (~ 58 kDa)
MCF7
Blot #6
75 kDa
50 kDa
MWM 47 91 C1 C2 77 K I C3 S 106 X 104 MWM

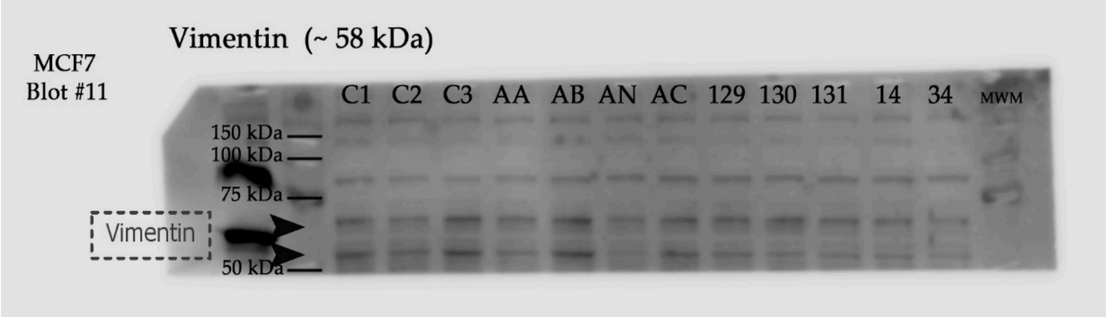
Vimentin (~ 58 kDa)
MCF7
Blot #11
C1 C2 C3 AA AB AN AC 129 130 131 14 34 MWM
150 kDa
100 kDa
75 kDa
Vimentin
50 kDa

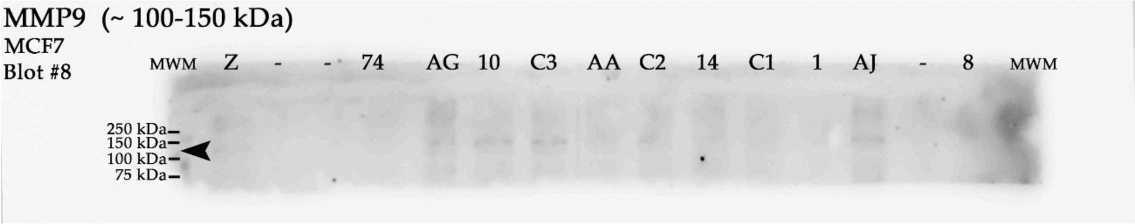
MMP9 (~ 100-150 kDa)
MCF7
Blot #8
MWM Z - - 74 AG 10 C3 AA C2 14 C1 1 AJ - 8 MWM
250 kDa
150 kDa
100 kDa
75 kDa

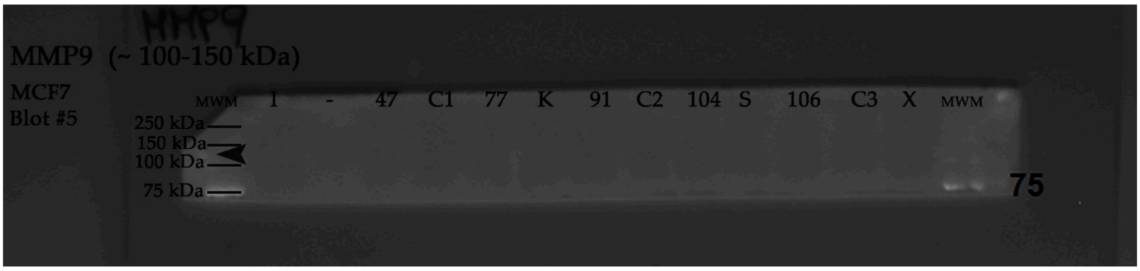
MMP9 (~ 100-150 kDa)
MCF7
Blot #5
MWM I - 47 C1 77 K 91 C2 104 S 106 C3 X MWM
250 kDa
150 kDa
100 kDa
75 kDa
75

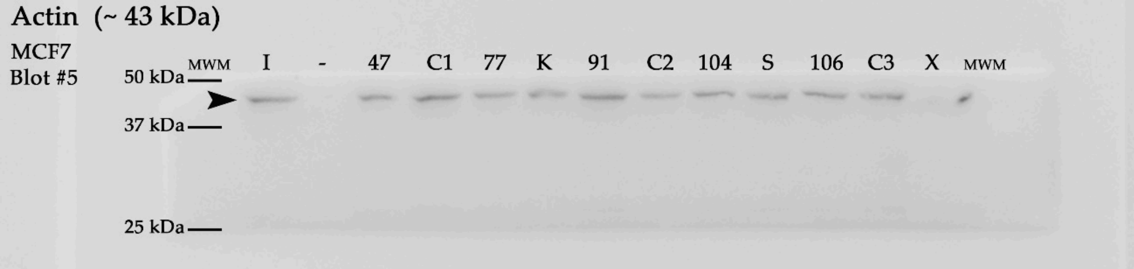
Actin (~ 43 kDa)
MCF7
Blot #5
MWM I - 47 C1 77 K 91 C2 104 S 106 C3 X MWM
50 kDa
37 kDa
25 kDa

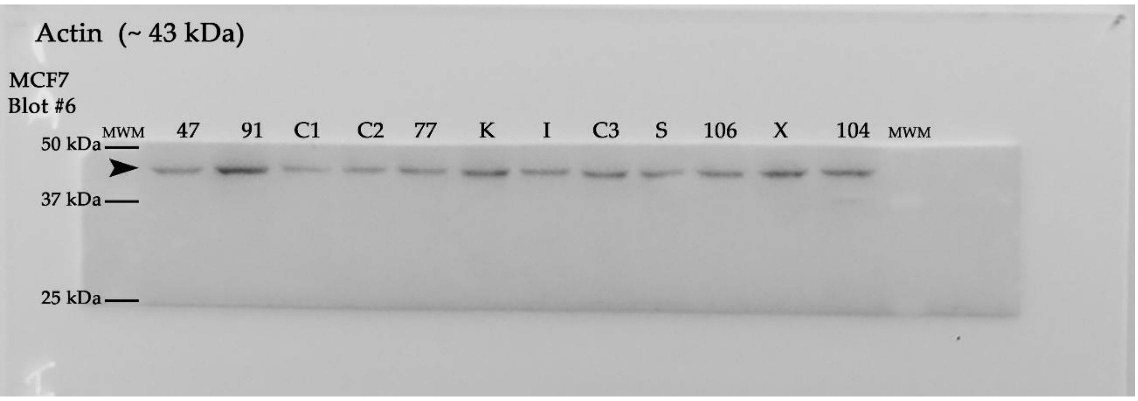
Actin (~ 43 kDa)
MCF7
Blot #6
MWM 47 91 C1 C2 77 K I C3 S 106 X 104 MWM
50 kDa
37 kDa
25 kDa

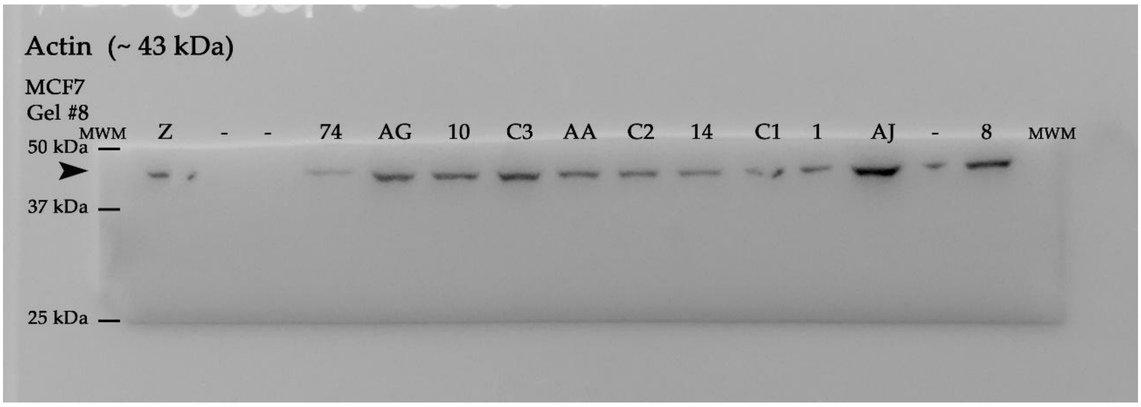
Actin (~ 43 kDa)
MCF7
Gel #8
MWM Z - - 74 AG 10 C3 AA C2 14 C1 1 AJ - 8 MWM
50 kDa
37 kDa
25 kDa

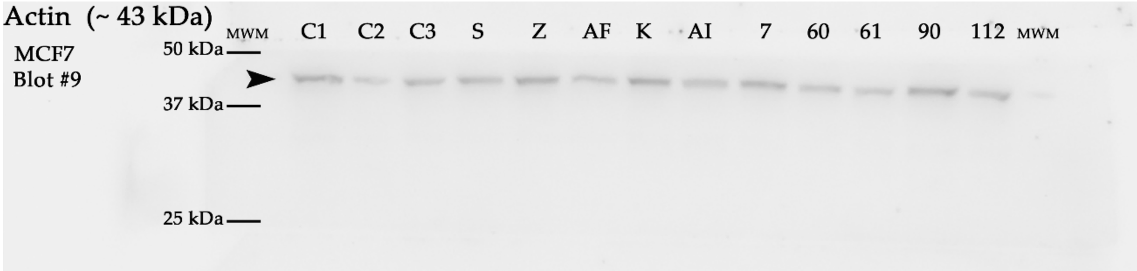
Actin (~ 43 kDa)
MCF7
Blot #9
MWM C1 C2 C3 S Z AF K AI 7 60 61 90 112 MWM
50 kDa
37 kDa
25 kDa

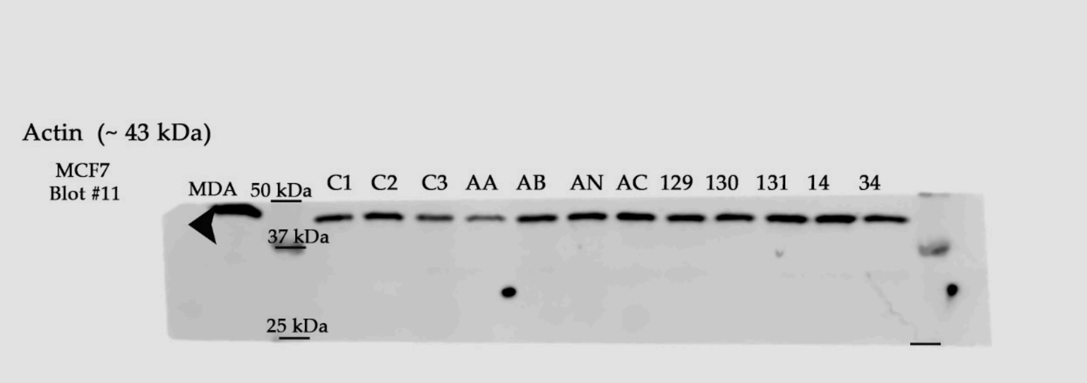
Actin (~ 43 kDa)
MCF7
Blot #11
MDA
50 kDa
37 kDa
25 kDa
C1 C2 C3 AA AB AN AC 129 130 131 14 34

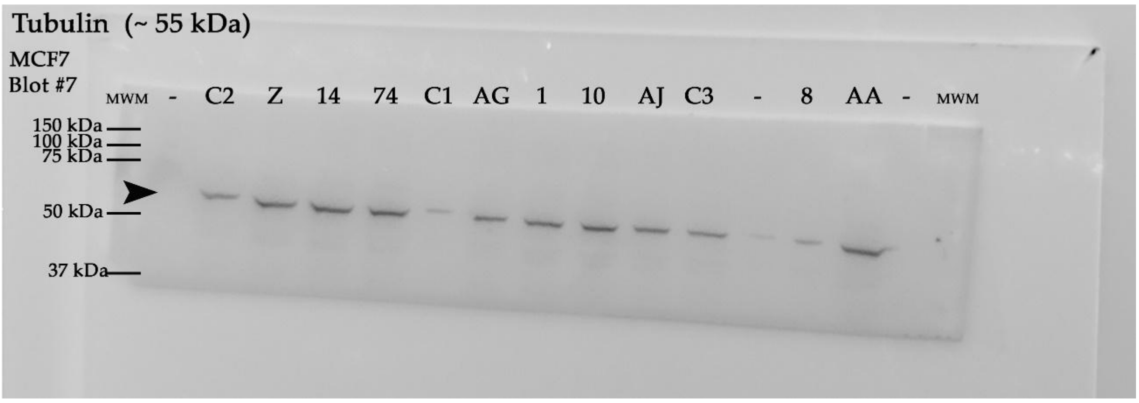
Tubulin (~ 55 kDa)
MCF7
Blot #7
MWM - C2 Z 14 74 C1 AG 1 10 AJ C3 - 8 AA - MWM
150 kDa
100 kDa
75 kDa
50 kDa
37 kDa

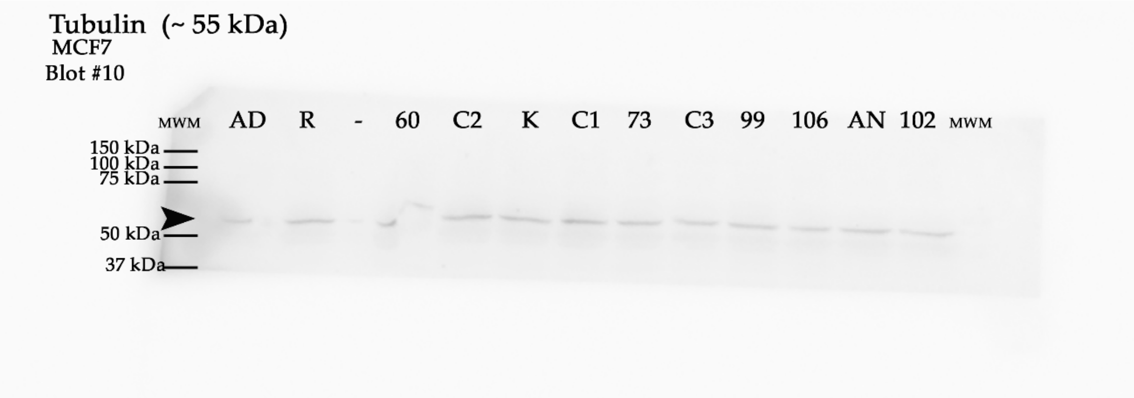
Tubulin (~ 55 kDa)
MCF7
Blot #10
MWM AD R - 60 C2 K C1 73 C3 99 106 AN 102 MWM
150 kDa
100 kDa
75 kDa
50 kDa
37 kDa

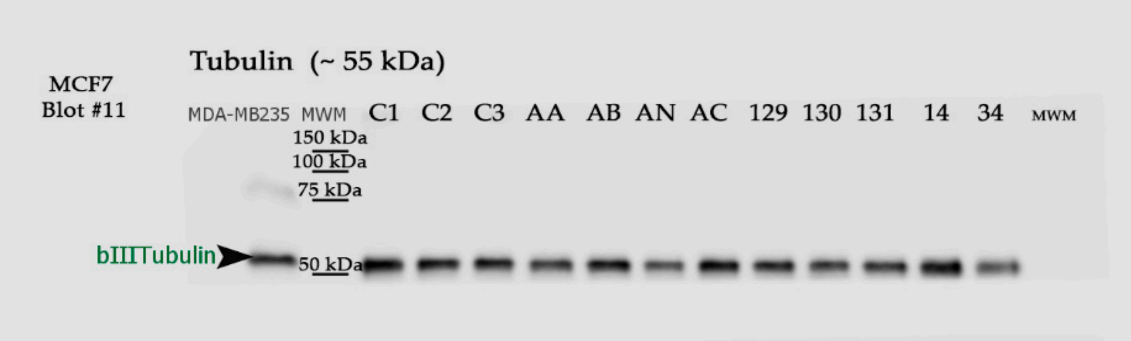
Tubulin (~ 55 kDa)
MCF7
Blot #11
MDA-MB235 MWM C1 C2 C3 AA AB AN AC 129 130 131 14 34 MWM
150 kDa
100 kDa
75 kDa
50 kDa
bIIITubulin
