# Supplementary material for: Microenvironment Modulates Tumorigenicity of Breast Cancer Cells Depending on Hormone Receptor Status
Source: Int J Mol Sci. 2026 Jan 22;27(2):1129. doi: 10.3390/ijms27021129 (PMC12842586; doi:10.3390/ijms27021129)
Supplement: Supplementary file 1 [file ijms-27-01129-s001.zip › Supplementary Figure S5.pdf]

Supplementary Figure S5. Original Western blot images showing protein expression of pluripotency and prognosis-related markers (SOX2, Nanog, OCT4, KLF4, caveolin-1, CD44, vimentin, and MMP9) in T47D tumor cells treated with different conditioned media (*control-*, *normal-*, and *adj*-CM). Actin or β-tubulin served as loading controls.

Arrows indicate the specific protein bands that were quantified: caveolin-1 doublet (~21–24 kDa), KLF4 doublet (~53 kDa), SOX2 band (~34 kDa), Nanog band (~40 kDa), OCT4 band (~45 kDa), vimentin doublet (~58 kDa), CD44 band (~80 kDa), MMP9 bands (~100–150 kDa), actin band (~43 kDa), and β-Tubulin (~55 kDa). Caveolin-1 detection was performed on four independent blots (blots 2, C, E, and H-2); KLF4 detection, on two independent blots (blots E and IV); SOX2 detection, on three independent blots (blots 2, C, and H-2); Nanog detection, on two independent blots (blots F, and H-1); OCT4 detection, on three independent blots (blots III, IV and H-1); vimentin detection, on four independent blots (blots C, E, H-2, and 2); CD44 detection, on three independent blots (blots C, B, and H-2); MMP9 detection, on two independent blots (blots E and D); actin detection, on the following blots: B, C, D, E, H-1, H-2, I, III, IV, and 2; β-Tubulin detection, on two independent blots (blots F and II). MWM, molecular weight markers.

Lanes labeled with a number correspond to protein lysates from T47D treated with *adjacent*-CM; Lanes labeled with different letters represent protein lysates from T47D treated with *normal*-CM; Lanes labeled with C1-3 represent protein lysates from T47D treated with *control*-CM; Lanes marked with an asterisk (*) indicate samples excluded from quantitative analysis because they did not meet the predefined quality criteria.

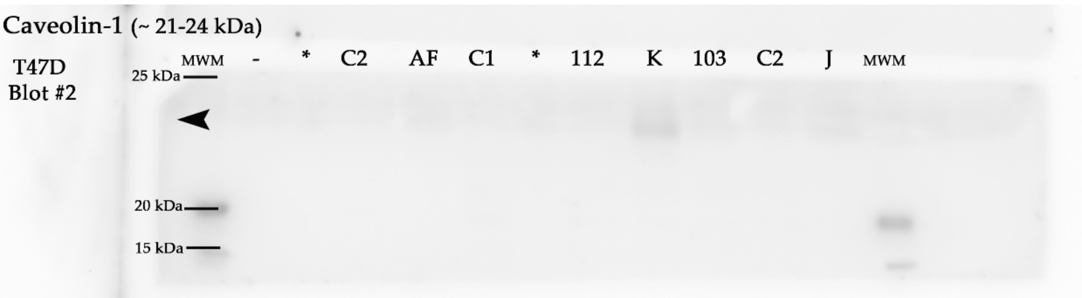

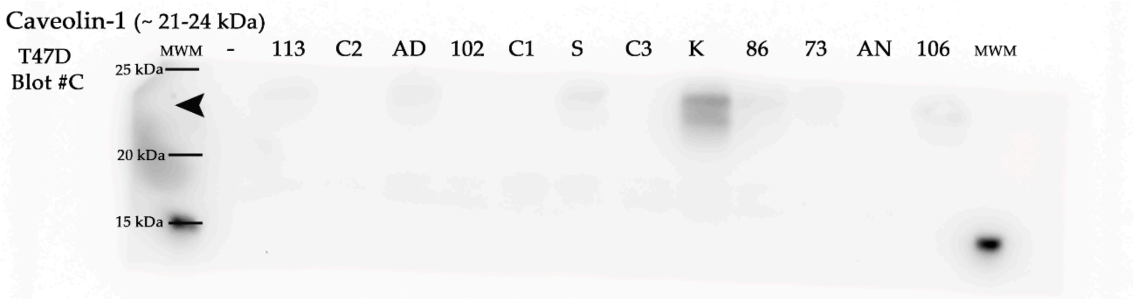

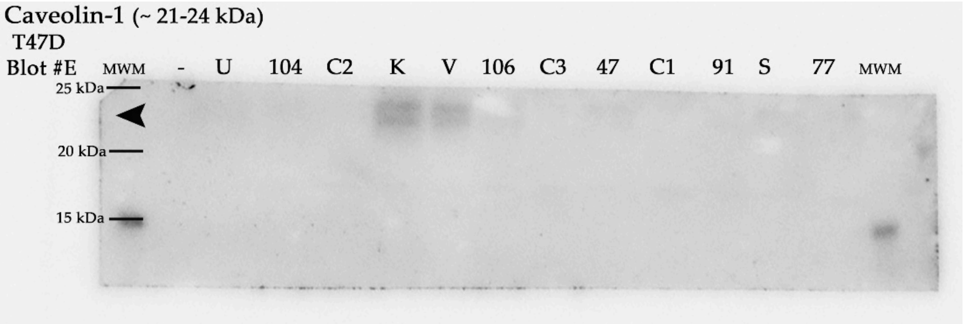

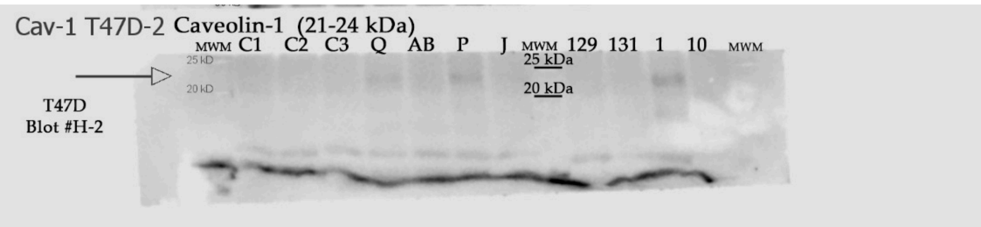

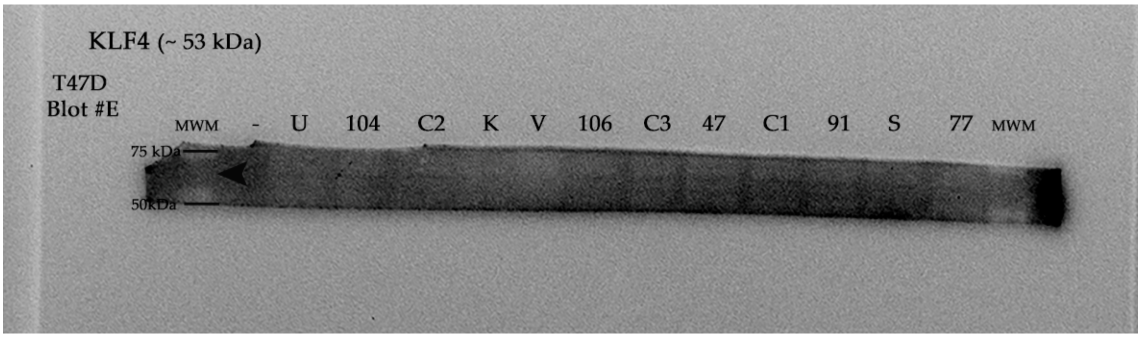
KLF4 (~ 53 kDa)
T47D
Blot #E
MWM - U 104 C2 K V 106 C3 47 C1 91 S 77 MWM
75 kDa
50kDa

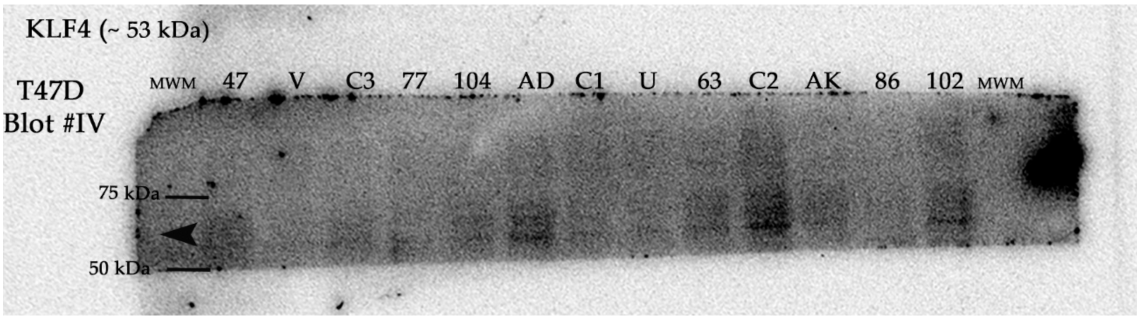
KLF4 (~ 53 kDa)
T47D
Blot #IV
MWM 47 V C3 77 104 AD C1 U 63 C2 AK 86 102 MWM
75 kDa
50 kDa

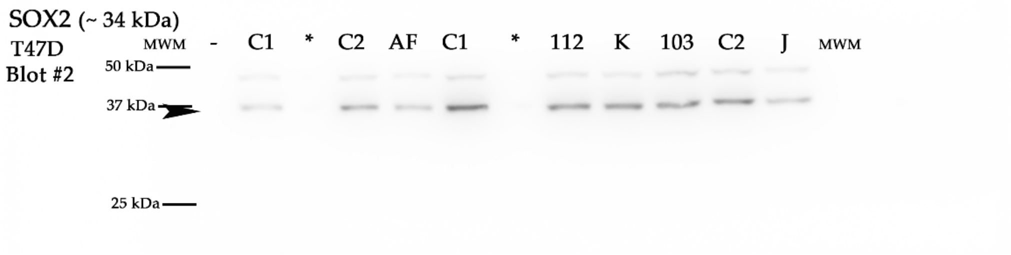
SOX2 (~ 34 kDa)
T47D
Blot #2
MWM - C1 * C2 AF C1 * 112 K 103 C2 J MWM
50 kDa
37 kDa
25 kDa

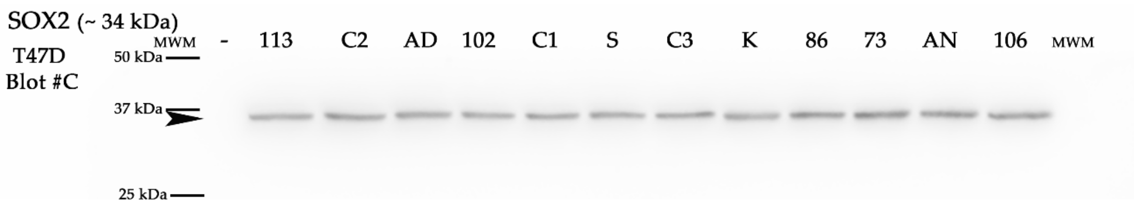
SOX2 (~ 34 kDa)
T47D
Blot #C
MWM - 113 C2 AD 102 C1 S C3 K 86 73 AN 106 MWM
50 kDa
37 kDa
25 kDa

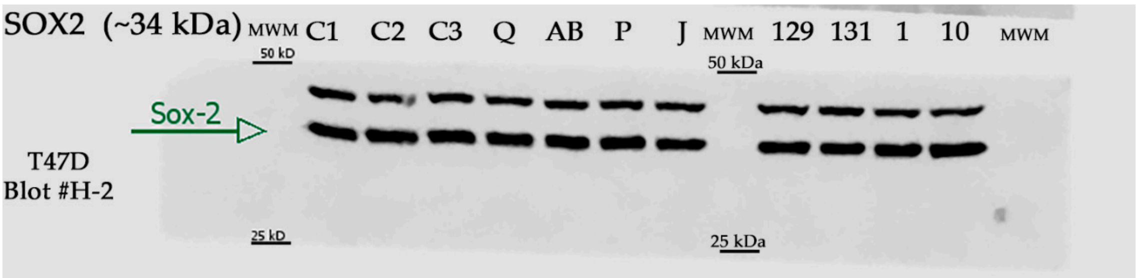
SOX2 (~34 kDa)
MWM C1 C2 C3 Q AB P J MWM 129 131 1 10 MWM
50 kD
50 kDa
Sox-2
T47D
Blot #H-2
25 kD
25 kDa

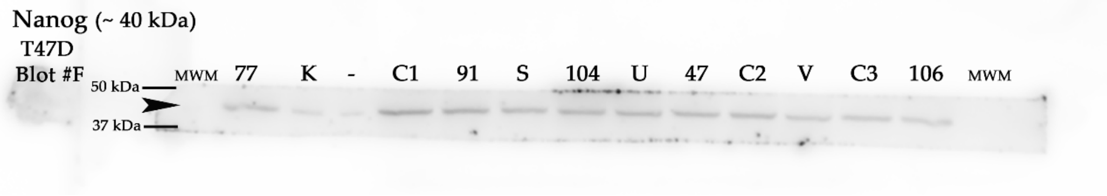
Nanog (~ 40 kDa)
T47D
Blot #F
MWM 77 K - C1 91 S 104 U 47 C2 V C3 106 MWM
50 kDa
37 kDa

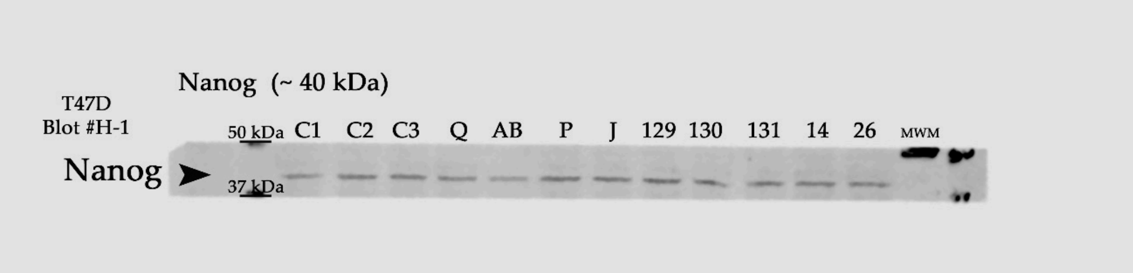
Nanog (~ 40 kDa)
T47D
Blot #H-1
Nanog
50 kDa
37 kDa
C1 C2 C3 Q AB P J 129 130 131 14 26 MWM

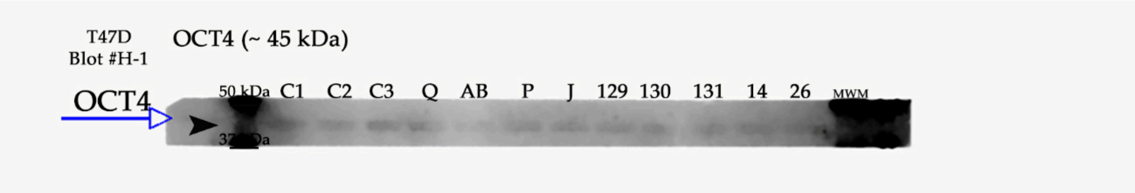
T47D
Blot #H-1
OCT4 (~ 45 kDa)
OCT4
50 kDa
C1 C2 C3 Q AB P J 129 130 131 14 26 MWM

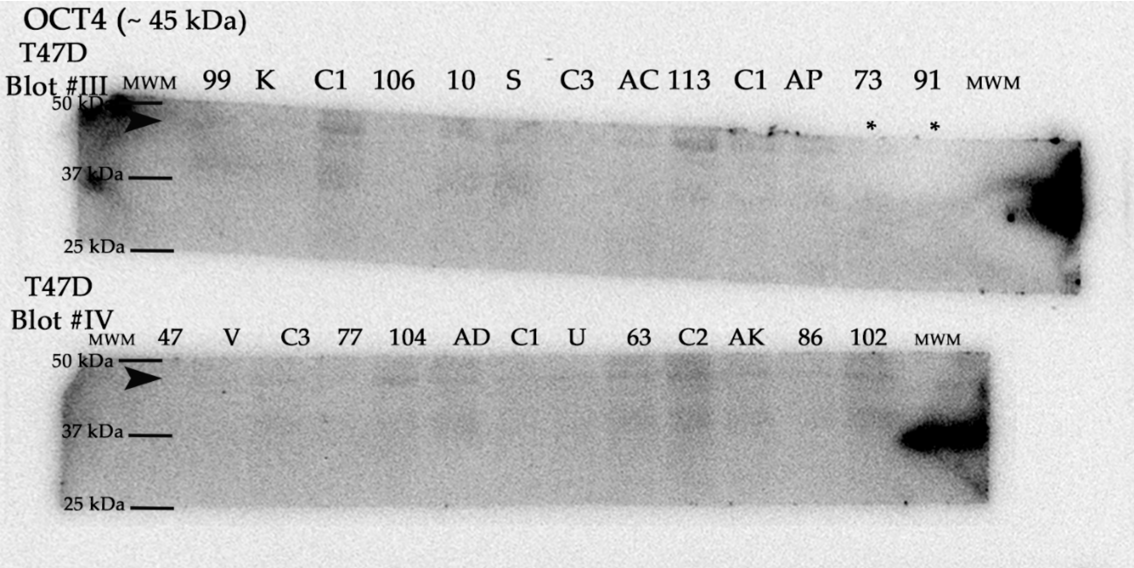
OCT4 (~ 45 kDa)
T47D
Blot #III
MWM 99 K C1 106 10 S C3 AC 113 C1 AP 73 91 MWM
37 kDa
25 kDa
*
*
T47D
Blot #IV
MWM 47 V C3 77 104 AD C1 U 63 C2 AK 86 102 MWM
50 kDa
37 kDa
25 kDa

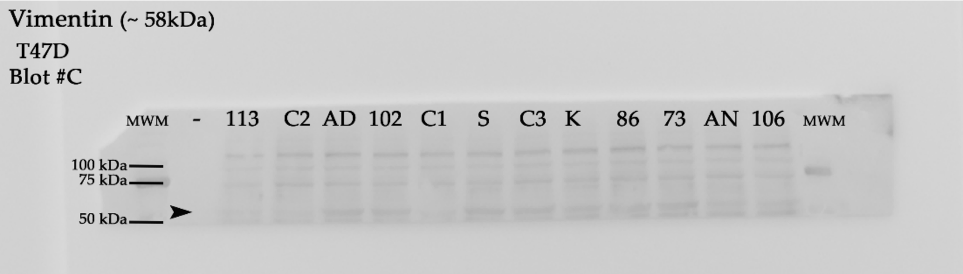
Vimentin (~ 58kDa)
T47D
Blot #C
MWM - 113 C2 AD 102 C1 S C3 K 86 73 AN 106 MWM
100 kDa
75 kDa
50 kDa

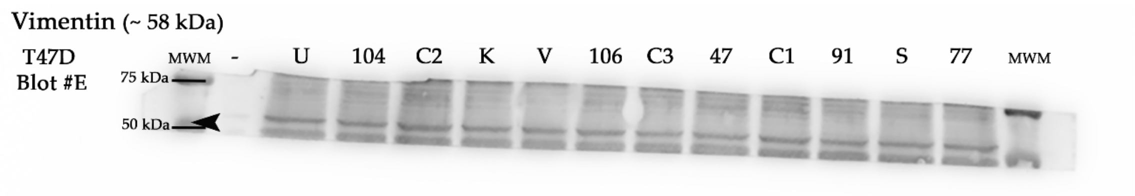
Vimentin (~ 58 kDa)
T47D
Blot #E
MWM - U 104 C2 K V 106 C3 47 C1 91 S 77 MWM
75 kDa
50 kDa

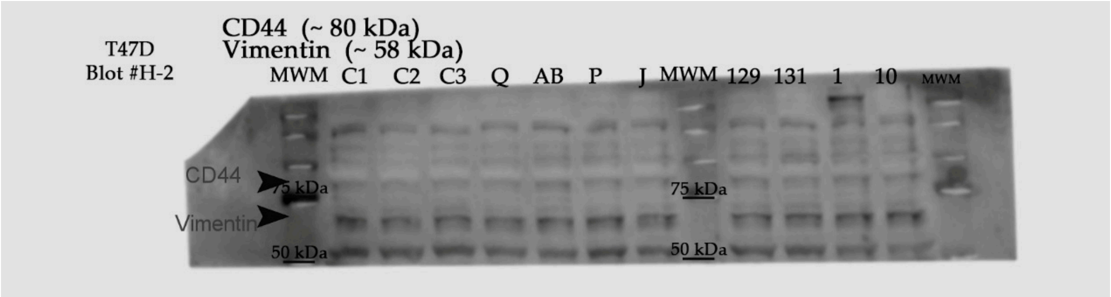
T47D
Blot #H-2
CD44 (~ 80 kDa)
Vimentin (~ 58 kDa)
MWM C1 C2 C3 Q AB P J MWM 129 131 1 10 MWM
CD44
Vimentin
75 kDa
50 kDa
75 kDa
50 kDa

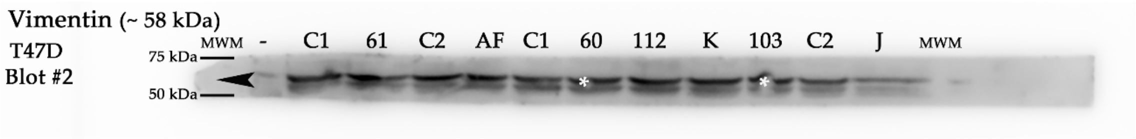
Vimentin (~ 58 kDa)
T47D
Blot #2
MWM - C1 61 C2 AF C1 60 112 K 103 C2 J MWM
75 kDa
50 kDa

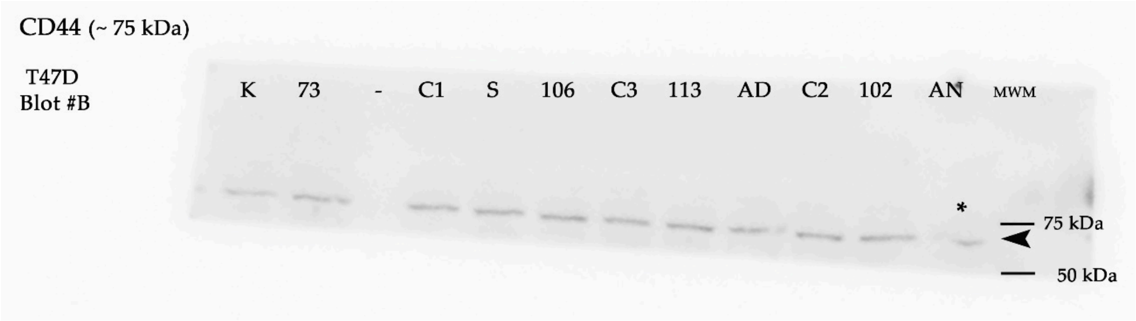
CD44 (~ 75 kDa)
T47D
Blot #B
K 73 - C1 S 106 C3 113 AD C2 102 AN MWM
75 kDa
50 kDa

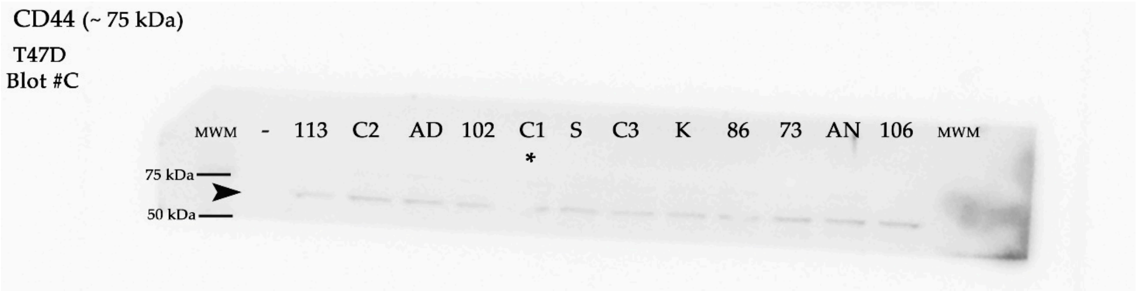
CD44 (~ 75 kDa)
T47D
Blot #C
MWM - 113 C2 AD 102 C1 S C3 K 86 73 AN 106 MWM
75 kDa
50 kDa

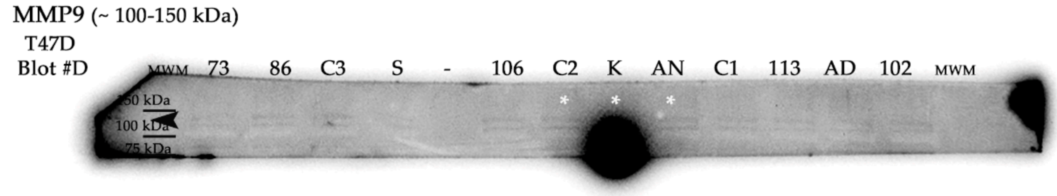
MMP9 (~ 100-150 kDa)
T47D
Blot #D
MWM 73 86 C3 S - 106 C2 K AN C1 113 AD 102 MWM
150 kDa
100 kDa
75 kDa

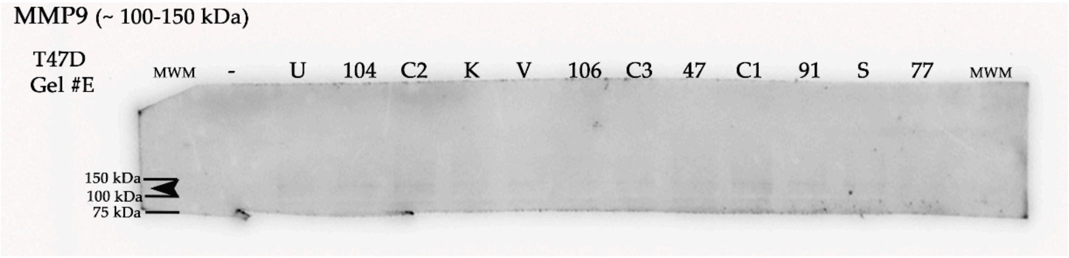
MMP9 (~ 100-150 kDa)
T47D
Gel #E
MWM - U 104 C2 K V 106 C3 47 C1 91 S 77 MWM
150 kDa
100 kDa
75 kDa

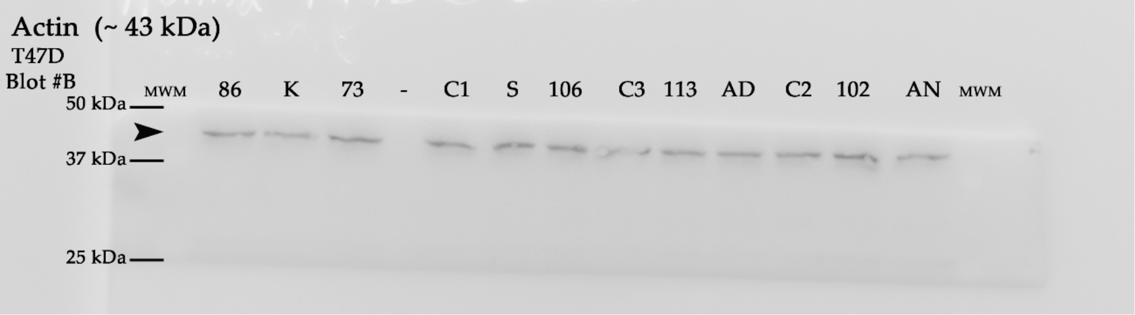
Actin (~ 43 kDa)
T47D
Blot #B
MWM 86 K 73 - C1 S 106 C3 113 AD C2 102 AN MWM
50 kDa
37 kDa
25 kDa

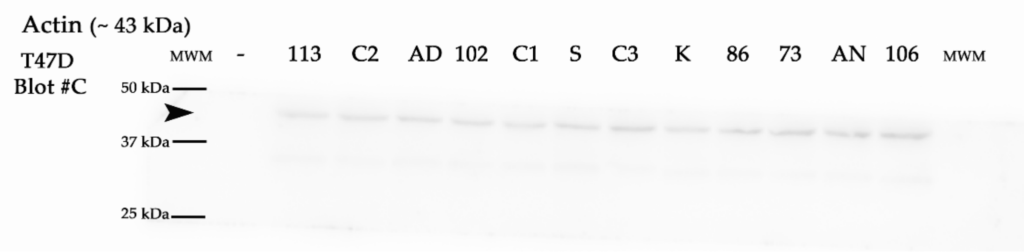
Actin (~ 43 kDa)
T47D
Blot #C
MWM - 113 C2 AD 102 C1 S C3 K 86 73 AN 106 MWM
50 kDa
37 kDa
25 kDa

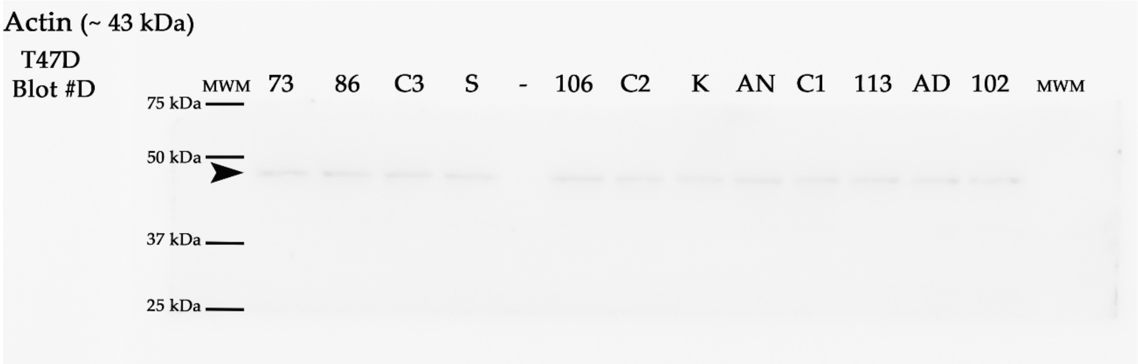
Actin (~ 43 kDa)
T47D
Blot #D
MWM 73 86 C3 S - 106 C2 K AN C1 113 AD 102 MWM
75 kDa
50 kDa
37 kDa
25 kDa

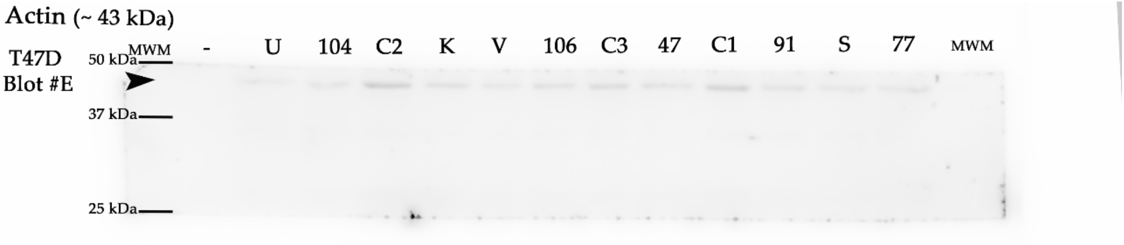
Actin (~ 43 kDa)
T47D
Blot #E
MWM - U 104 C2 K V 106 C3 47 C1 91 S 77 MWM
50 kDa
37 kDa
25 kDa

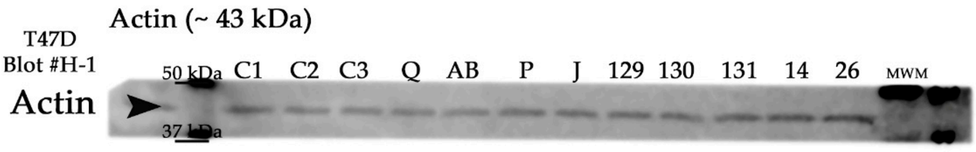
Actin (~ 43 kDa)
T47D
Blot #H-1
Actin
50 kDa C1 C2 C3 Q AB P J 129 130 131 14 26 MWM
37 kDa

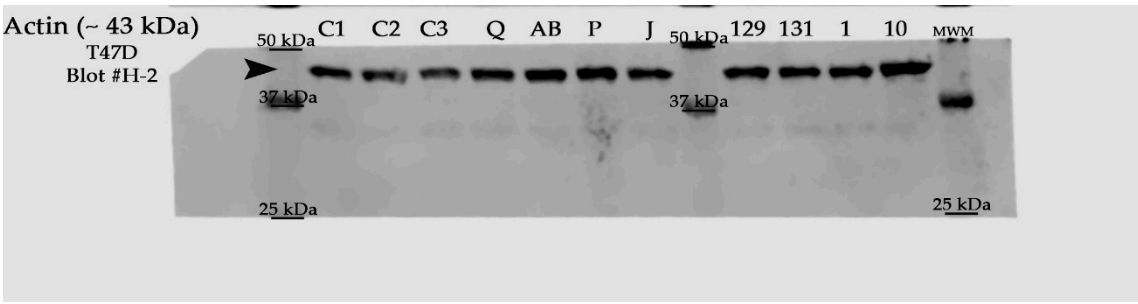
Actin (~ 43 kDa)
T47D
Blot #H-2
50 kDa C1 C2 C3 Q AB P J 50 kDa 129 131 1 10 MWM
37 kDa
37 kDa
25 kDa
25 kDa

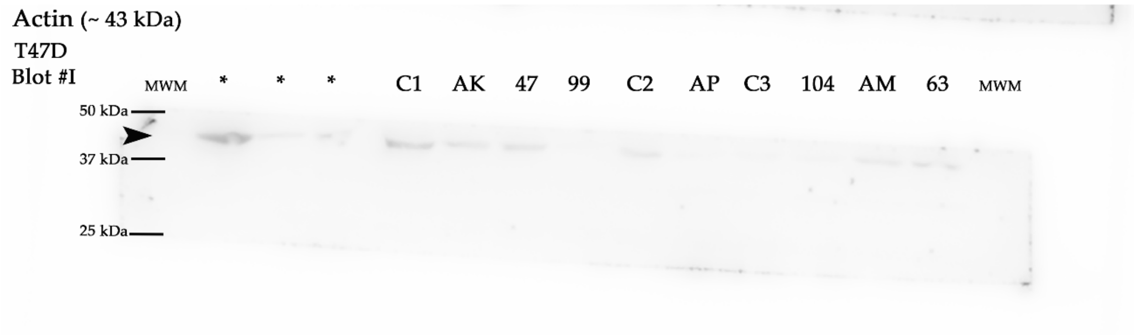
Actin (~ 43 kDa)
T47D
Blot #I
MWM * * * C1 AK 47 99 C2 AP C3 104 AM 63 MWM
50 kDa
37 kDa
25 kDa

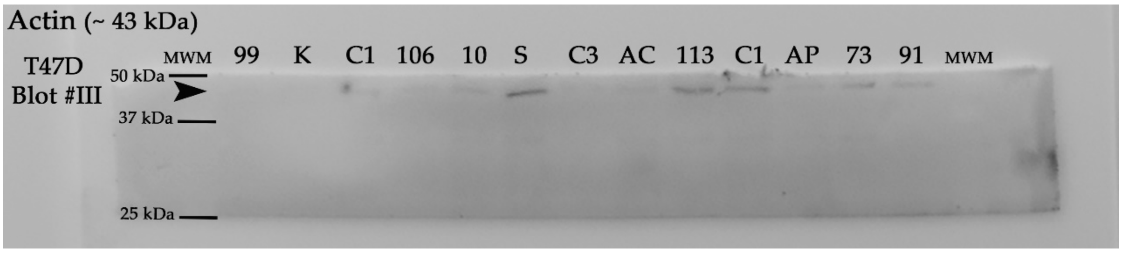
Actin (~ 43 kDa)
T47D
Blot #III
MWM 99 K C1 106 10 S C3 AC 113 C1 AP 73 91 MWM
50 kDa
37 kDa
25 kDa

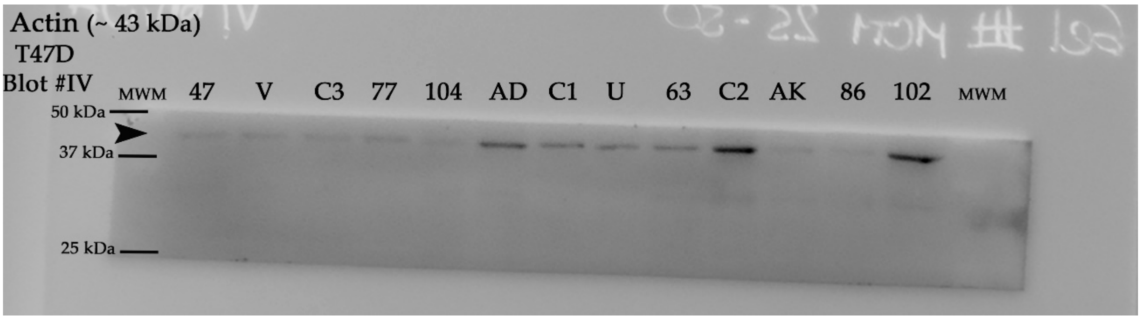
Actin (~ 43 kDa)
T47D
Blot #IV
MWM 47 V C3 77 104 AD C1 U 63 C2 AK 86 102 MWM
50 kDa
37 kDa
25 kDa

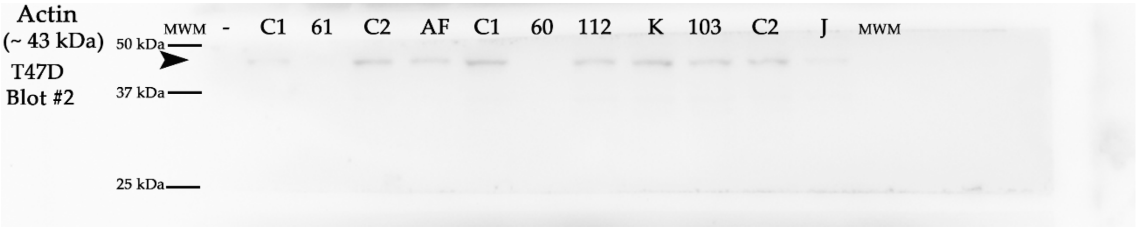
Actin
(~ 43 kDa)
T47D
Blot #2
MWM - C1 61 C2 AF C1 60 112 K 103 C2 J MWM
50 kDa
37 kDa
25 kDa

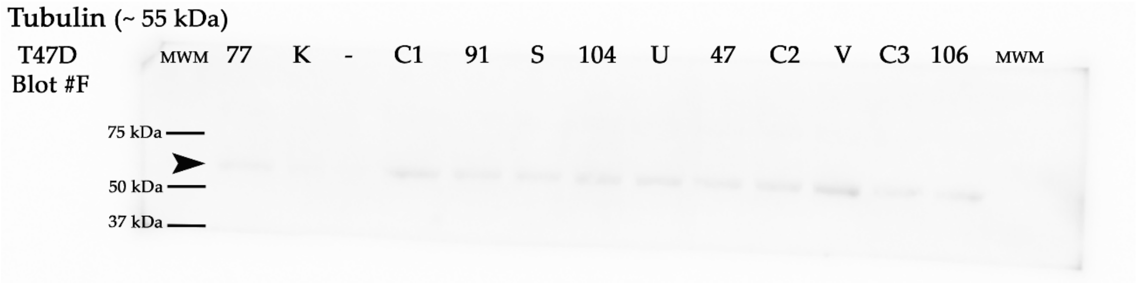
Tubulin (~ 55 kDa)
T47D
Blot #F
MWM 77 K - C1 91 S 104 U 47 C2 V C3 106 MWM
75 kDa
50 kDa
37 kDa

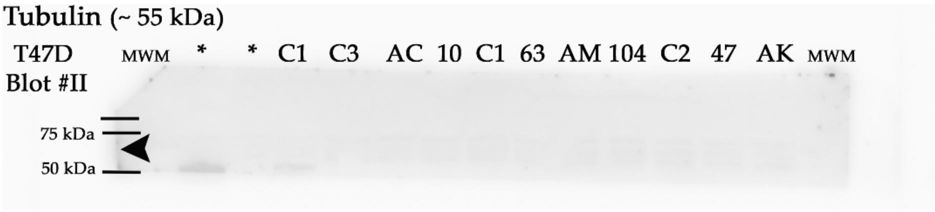
Tubulin (~ 55 kDa)
T47D
Blot #II
MWM * * C1 C3 AC 10 C1 63 AM 104 C2 47 AK MWM
75 kDa
50 kDa
