# Supplementary material for: Microenvironment Modulates Tumorigenicity of Breast Cancer Cells Depending on Hormone Receptor Status
Source: Int J Mol Sci. 2026 Jan 22;27(2):1129. doi: 10.3390/ijms27021129 (PMC12842586; doi:10.3390/ijms27021129)
Supplement: Supplementary file 1 [file ijms-27-01129-s001.zip › Supplementary Figure S6.pdf]

Supplementary Figure S6. Original Western blot images showing protein expression of pluripotency and prognosis-related markers (SOX2, Nanog, OCT4, KLF4, caveolin-1, CD44, and MMP9) in MDA-MB-231 tumor cells treated with different conditioned media (*control-*, *normal-*, and *adj*-CM). Actin served as loading controls.

Arrows indicate the specific protein bands that were quantified: caveolin-1 doublet (~21–24 kDa), KLF4 doublet (~53 kDa), SOX2 band (~34 kDa), Nanog band (~40 kDa), OCT4 doublet (~39-45 kDa), CD44 band (~80 kDa), MMP9 bands (~100–150 kDa), and actin band (~43 kDa). Caveolin-1 detection was performed on two independent blots (blots 2, and I); KLF4 detection, on two independent blots (blots I and II); SOX2 detection, on two independent blots (blots 1 and I); Nanog detection, on two independent blots (blots 1 and III); OCT4 detection, on two independent blots (blots I and II); CD44 detection, on two independent blots (blots I and III); MMP9 detection, on two independent blots (blots I and II); actin detection, on the following blots: 1, 2, I, II, and III. MWM, molecular weight markers.

Lanes labeled with a number correspond to protein lysates from MDA-MB-231 treated with *adjacent*-CM; Lanes labeled with different letters represent protein lysates from MDA-MB-231 treated with *normal*-CM; Lanes labeled with C1-3 represent protein lysates from MDA-MB-231 treated with *control*-CM; Lanes marked with an asterisk (*) indicate samples excluded from quantitative analysis because they did not meet the predefined quality criteria.

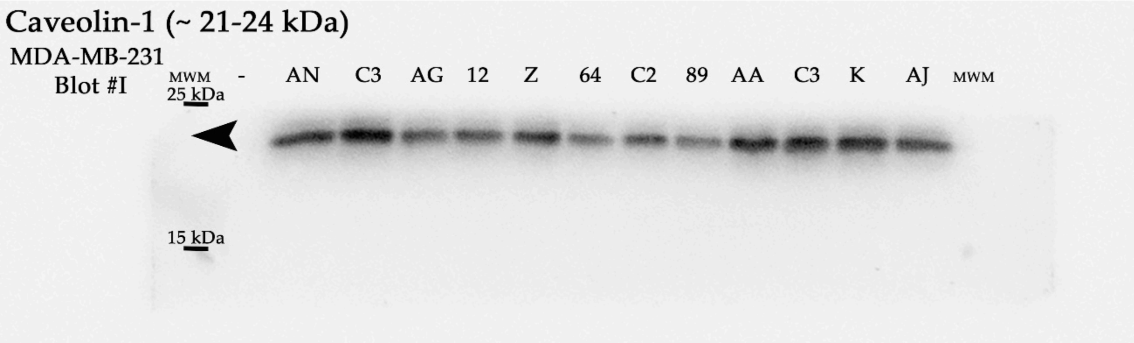

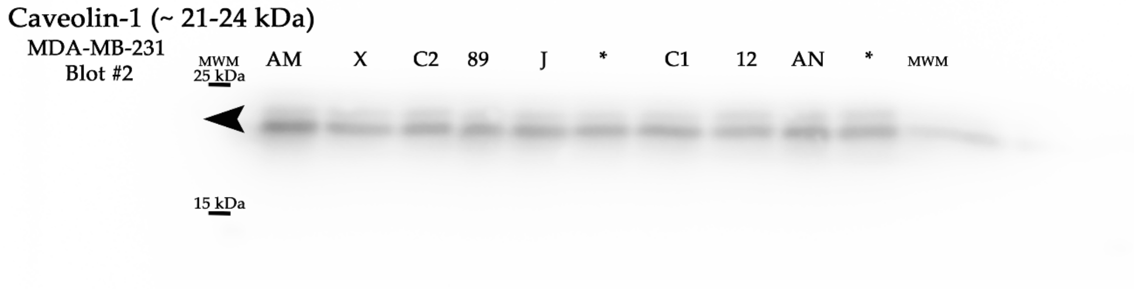

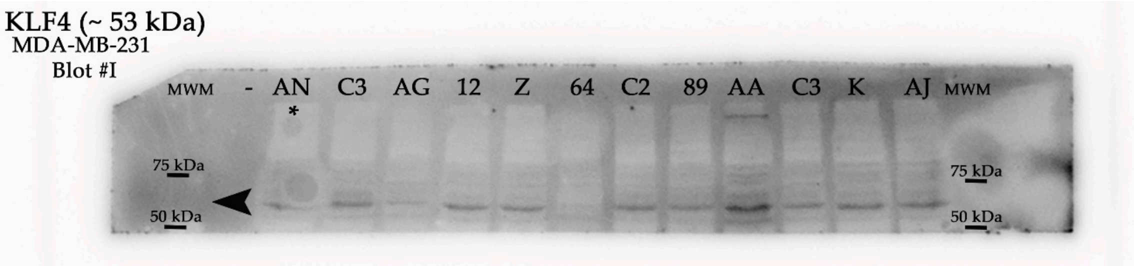

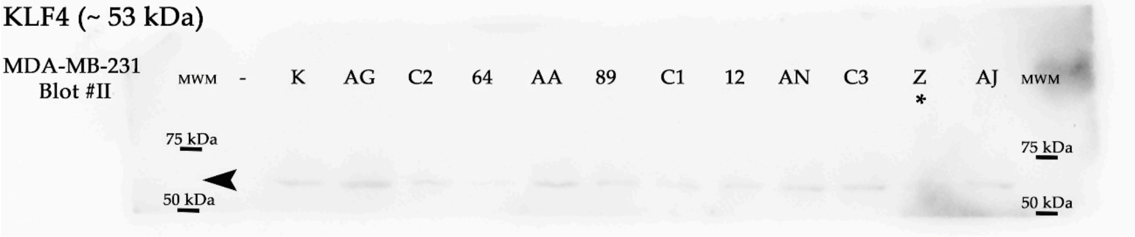

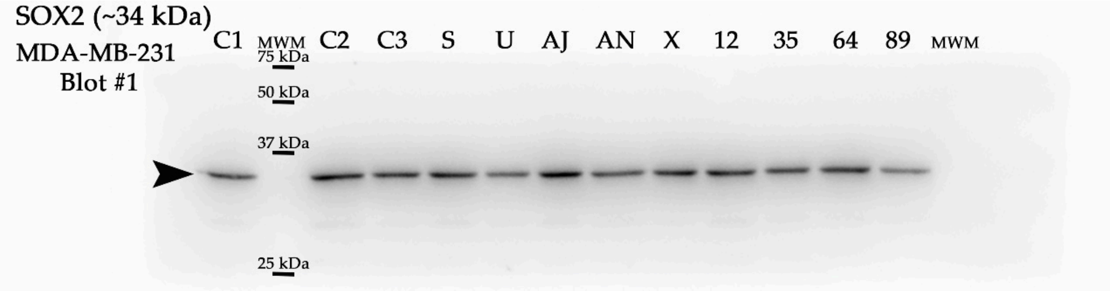
SOX2 (~34 kDa)
MDA-MB-231
Blot #1
C1 MWM C2 C3 S U AJ AN X 12 35 64 89 MWM
75 kDa
50 kDa
37 kDa
25 kDa

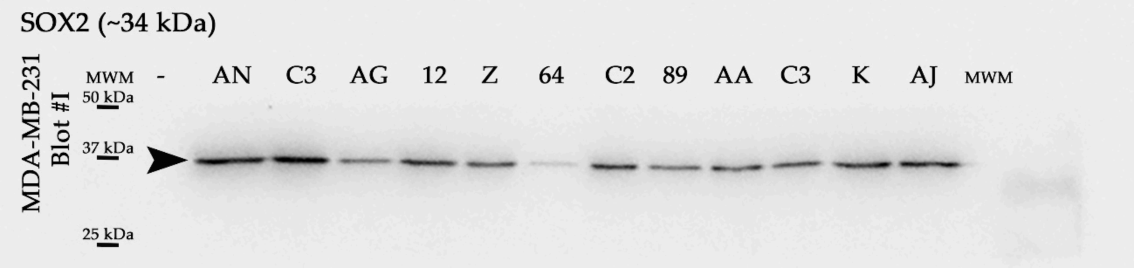
SOX2 (~34 kDa)
MDA-MB-231
Blot #I
MWM - AN C3 AG 12 Z 64 C2 89 AA C3 K AJ MWM
50 kDa
37 kDa
25 kDa

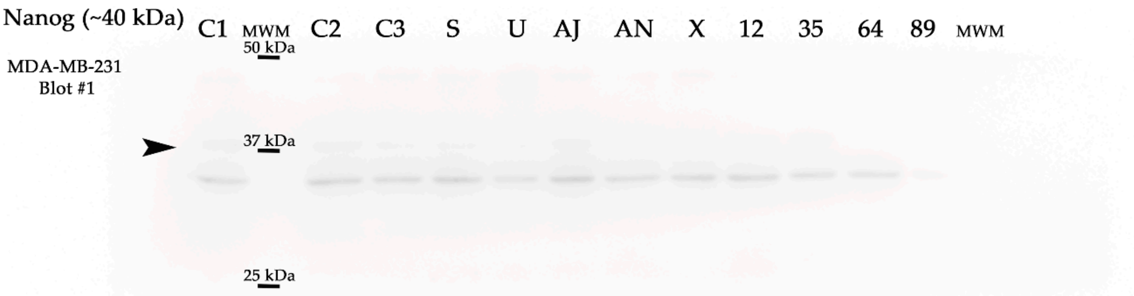
Nanog (~40 kDa)
MDA-MB-231
Blot #1
C1 MWM C2 C3 S U AJ AN X 12 35 64 89 MWM
50 kDa
37 kDa
25 kDa

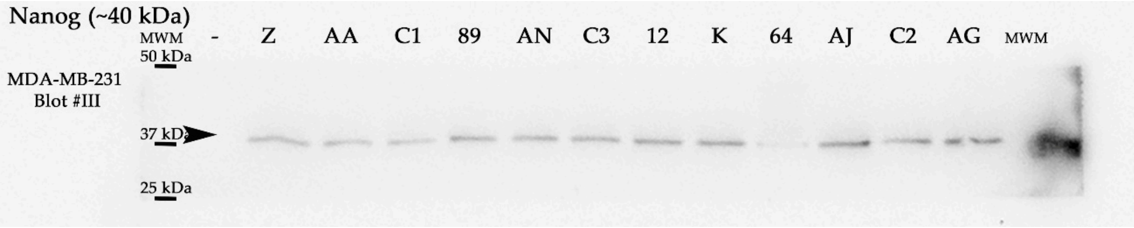
Nanog (~40 kDa)
MDA-MB-231
Blot #III
MWM - Z AA C1 89 AN C3 12 K 64 AJ C2 AG MWM
50 kDa
37 kDa
25 kDa

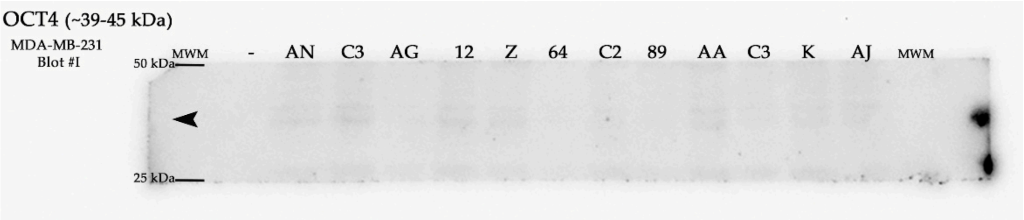
OCT4 (~39-45 kDa)
MDA-MB-231
Blot #I
MWM - AN C3 AG 12 Z 64 C2 89 AA C3 K AJ MWM
50 kDa
25 kDa

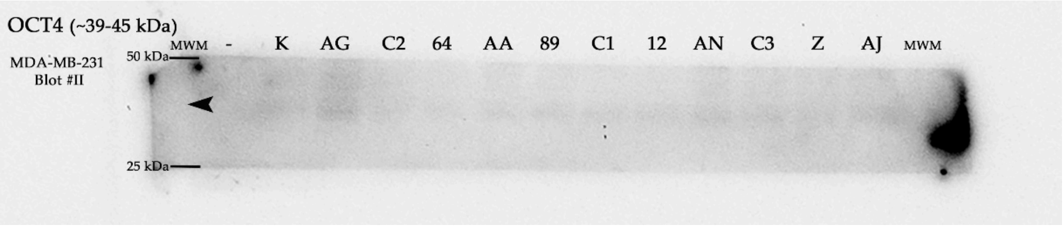
OCT4 (~39-45 kDa)
MDA-MB-231
Blot #II
MWM - K AG C2 64 AA 89 C1 12 AN C3 Z AJ MWM
50 kDa
25 kDa

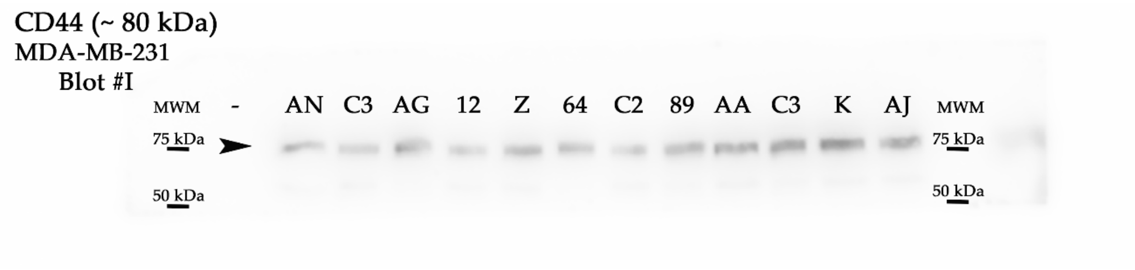
CD44 (~ 80 kDa)
MDA-MB-231
Blot #I
MWM - AN C3 AG 12 Z 64 C2 89 AA C3 K AJ MWM
75 kDa
50 kDa
75 kDa
50 kDa

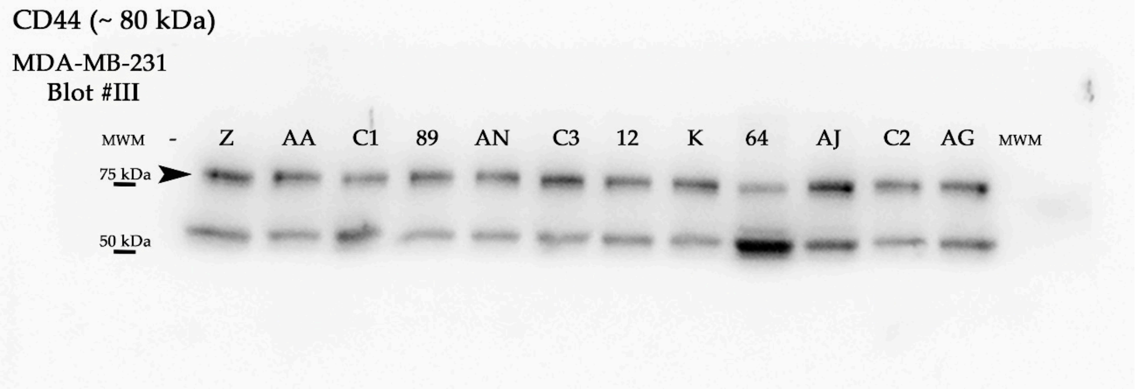
CD44 (~ 80 kDa)
MDA-MB-231
Blot #III
MWM - Z AA C1 89 AN C3 12 K 64 AJ C2 AG MWM
75 kDa
50 kDa

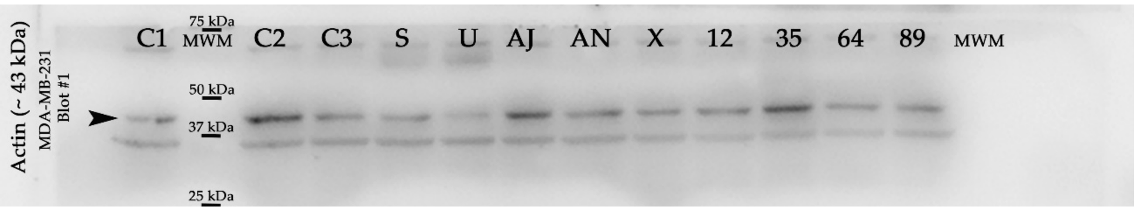
Actin (~ 43 kDa)
MDA-MB-231
Blot #1
C1 MWM C2 C3 S U AJ AN X 12 35 64 89 MWM
75 kDa
50 kDa
37 kDa
25 kDa

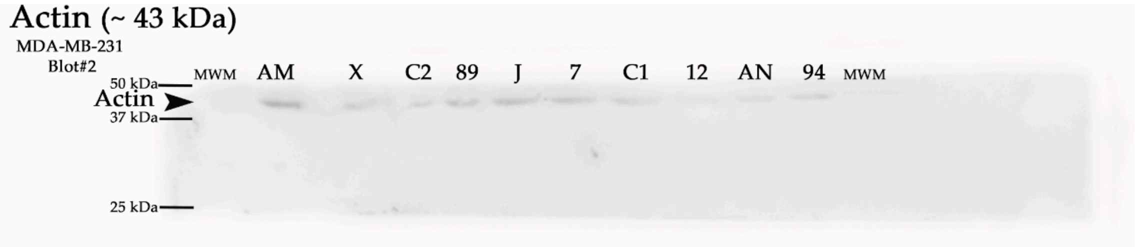
Actin (~ 43 kDa)
MDA-MB-231
Blot#2
MWM AM X C2 89 J 7 C1 12 AN 94 MWM
50 kDa
Actin
37 kDa
25 kDa

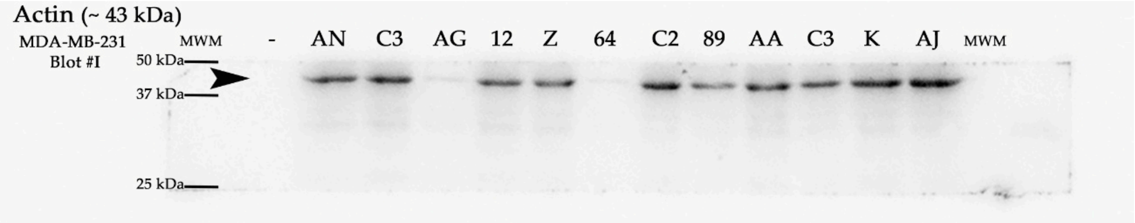
Actin (~ 43 kDa)
MDA-MB-231
Blot #I
MWM - AN C3 AG 12 Z 64 C2 89 AA C3 K AJ MWM
50 kDa
37 kDa
25 kDa

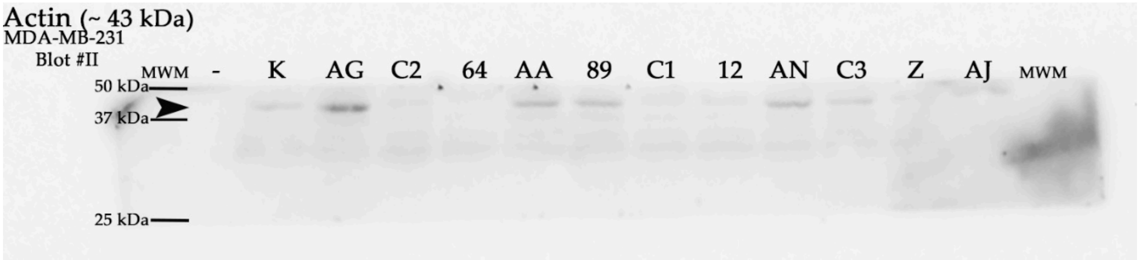
Actin (~ 43 kDa)
MDA-MB-231
Blot #II
MWM - K AG C2 64 AA 89 C1 12 AN C3 Z AJ MWM
50 kDa
37 kDa
25 kDa

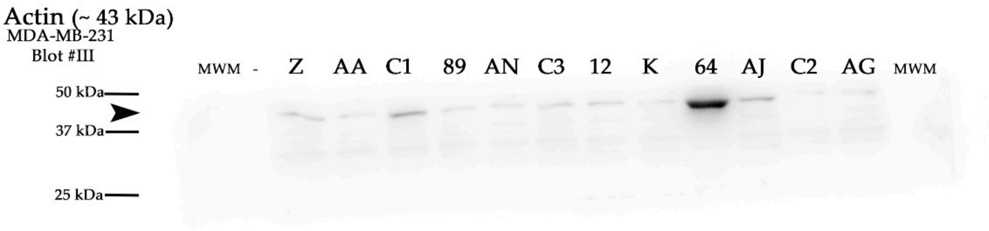
Actin (~ 43 kDa)
MDA-MB-231
Blot #III
MWM
-
Z
AA
C1
89
AN
C3
12
K
64
AJ
C2
AG
MWM
50 kDa
37 kDa
25 kDa
