# Supplementary material for: Microenvironment Modulates Tumorigenicity of Breast Cancer Cells Depending on Hormone Receptor Status
Source: Int J Mol Sci. 2026 Jan 22;27(2):1129. doi: 10.3390/ijms27021129 (PMC12842586; doi:10.3390/ijms27021129)
Supplement: Supplementary file 1 [file ijms-27-01129-s001.zip › Supplementary Table S1.pdf]

Supplementary Table S1. Differential genes expression in BC models. Pairwise comparison between HR+ BC (MCF7 and T47D) and TNBC (MDA-MB-231) models for genes expression of pluripotency and prognostic markers. Overexpression is represented in bold, and underexpression is highlighted in gray.

	**Genes**	**NM**	**MDA-MB-231 Respect to**				**T47D Respect to**	
			**MCF7**		**T47D**		**MCF7**	
			**Adj. p Value**	**log FC**	**Adj. p Value**	**log FC**	**Adj. p Value**	**log FC**
**Pluripotency markers**	***SOX2***	NM_003106	3.33×10^{-4}	**7.30**	4.11×10^{-4}	**6.92**	0.03	**0.38**
	POU5F1	NM_002701	0.06	0.35	0.55	−0.09	0.04	**0.44**
	NANOG	NM_024865	0.01	**2.24**	0.02	−2.16	2.11×10^{-3}	**4.40**
	KLF4	NM_004235	9.36×10^{-3}	−1.23	3.65×10^{-3}	−2.79	0.01	**1.57**
**Prognosis markers**	***CAV-1***	NM_001753	3.07×10^{-4}	−6.68	3.64×10^{-4}	−12.59	3.07×10^{-4}	**6.13**
	MMP9	NM_004994	7.20×10^{-4}	**3.07**	2.13×10^{-3}	**1.86**	1.89×10^{-3}	**1.21**
	CD44	NM_000610	5.00×10^{-4}	−3.31	3.97×10^{-4}	−5.31	1.23×10^{-3}	**2.00**
	VIM	NM_003380	3.07×10^{-4}	−10.72	3.64×10^{-4}	−9.74	8.37×10^{-3}	−0.99

Adj. p value, adjusted p value; FC, fold change; NM_, RefSeq accession codes.
