# Supplementary material for: Microenvironment Modulates Tumorigenicity of Breast Cancer Cells Depending on Hormone Receptor Status
Source: Int J Mol Sci. 2026 Jan 22;27(2):1129. doi: 10.3390/ijms27021129 (PMC12842586; doi:10.3390/ijms27021129)
Supplement: Supplementary file 1 [file ijms-27-01129-s001.zip › Supplementary Table S2.pdf]

Supplementary Table S2. Antibodies used for Western blot and indirect immunofluorescence assays. Antibodies, catalog numbers, and dilutions used for protein analysis of AT and breast cancer cell lines.

	List of Antibodies	Catalog	Supplier	Dilution		Host	Clonality
				Adipose Tissue	Tumor celL LINES		
Primary antibody	Actin	I19 sc-1616R	Santa Cruz	1:1000		Rabbit	Polyclonal
	Adiponectina	ab22554	abcam	1:1000	-	Mouse	Monoclonal
	CD44	ab157107	abcam	1:2000		Rabbit	Polyclonal
	Caveolin-1	610060 BD	BD Biosciences	1:2000	1:1000	Rabbit	Polyclonal
	FABP4	ab219595	abcam	1:2000	-	Rabbit	Monoclonal
	KLF4	sc-166238	Santa Cruz	-	1:1000	Mouse	Monoclonal
	MMP9	AF909	R&D Systems	1:1000		Goat	Polyclonal
	Nanog	sc-293121	Santa Cruz	-	1:1000	Mouse	Monoclonal
	Oct3/4	sc-5279	Santa Cruz	-	1:1000	Mouse	Monoclonal
	SOX2	MA5-31455	Invitrogen	-	1:1000	Mouse	Monoclonal
	β-Tubulin	(G-8): sc-55529	Santa Cruz	-	1:1000	Mouse	Monoclonal
	β III Tubulin	ab52623	abcam	-	1:1000	Rabbit	Monoclonal
	Vimentin	V4630	Sigma-Aldrich	1:2000		Goat	Polyclonal
Secondary antibody	Anti-goat IgG-HRP	PA1-28664	Invitrogen	1:20000			
	Anti-mouse IgG-HRP	sc-2005	Santa Cruz	1:4000			
	Anti-mouse IgG kappa light chain-HRP	sc-516102	Santa Cruz	1:4000			
	Anti-rabbit IgG-HRP	A0545	Sigma-Aldrich	1:6000			
Fluorescent probes	Alexa Fluor™ 488-conjugated phalloidin	A12379	Thermo Fisher Scientific	1:50			
	Hoechst 33342	H3570	Thermo Fisher Scientific	1:1000			
