# Supplementary material for: Microenvironment Modulates Tumorigenicity of Breast Cancer Cells Depending on Hormone Receptor Status
Source: Int J Mol Sci. 2026 Jan 22;27(2):1129. doi: 10.3390/ijms27021129 (PMC12842586; doi:10.3390/ijms27021129)
Supplement: Supplementary file 1 [file ijms-27-01129-s001.zip › Supplementary Table S3.pdf]

Supplementary Table S3. List of human-specific primer sequences used for qPCR amplification of target genes.

Gen	Forward (5´→3´)	Reverse (5´→3´)
ADIPOQ	GGAGATCCAGGTCTTATTGG	TGGGCATGTTGGGGATAGTA
CAV-1	GCGACCCTAAACACCTCAAC	ATGCCGTCAAAACTGTGTGTC
FABP4	GGCCAGGAATTTGACGAAGT	ATCCCACAGAATGTTGTAGAGT
VIM	CAGGAGGCAGAAGAATGGTACAAA	GGCGTTCCAGGGACTCATTG
CYC	CTTCCCCGATGAGAACTTCA	TCTTGGTGCTCTCCACCTTC
HPRT	GCTGAGGATTTGGAAAGGGTG	AATCCAGCAGGTCAGCAAAG
